# Mechanical, Material and Morphological Adaptations of Healthy Lower Limb Tendons to Mechanical Loading: A Systematic Review and Meta-Analysis

**DOI:** 10.1007/s40279-022-01695-y

**Published:** 2022-06-03

**Authors:** Stephanie L. Lazarczuk, Nirav Maniar, David A. Opar, Steven J. Duhig, Anthony Shield, Rod S. Barrett, Matthew N. Bourne

**Affiliations:** 1 School of Health Sciences and Social Work, Griffith University, Gold Coast, QLD Australia; 2 Griffith Centre of Biomedical and Rehabilitation Engineering (GCORE), Menzies Health Institute Queensland, Griffith University, Gold Coast, QLD Australia; 3 School of Behavioural and Health Sciences, Australian Catholic University, Melbourne, VIC Australia; 4 Sports Performance, Recovery, Injury and New Technologies (SPRINT) Research Centre, Australian Catholic University, Melbourne, VIC Australia; 5 School of Exercise and Nutrition Sciences and Institute of Health and Biomedical Innovation, Queensland University of Technology, Brisbane, QLD Australia

## Abstract

**Background:**

Exposure to increased mechanical loading during physical training can lead to increased tendon stiffness. However, the loading regimen that maximises tendon adaptation and the extent to which adaptation is driven by changes in tendon material properties or tendon geometry is not fully understood.

**Objective:**

To determine (1) the effect of mechanical loading on tendon stiffness, modulus and cross-sectional area (CSA); (2) whether adaptations in stiffness are driven primarily by changes in CSA or modulus; (3) the effect of training type and associated loading parameters (relative intensity; localised strain, load duration, load volume and contraction mode) on stiffness, modulus or CSA; and (4) whether the magnitude of adaptation in tendon properties differs between age groups.

**Methods:**

Five databases (PubMed, Scopus, CINAHL, SPORTDiscus, EMBASE) were searched for studies detailing load-induced adaptations in tendon morphological, material or mechanical properties. Standardised mean differences (SMDs) with 95% confidence intervals (CIs) were calculated and data were pooled using a random effects model to estimate variance. Meta regression was used to examine the moderating effects of changes in tendon CSA and modulus on tendon stiffness.

**Results:**

Sixty-one articles met the inclusion criteria. The total number of participants in the included studies was 763. The Achilles tendon (33 studies) and the patella tendon (24 studies) were the most commonly studied regions. Resistance training was the main type of intervention (49 studies). Mechanical loading produced moderate increases in stiffness (standardised mean difference (SMD) 0.74; 95% confidence interval (CI) 0.62–0.86), large increases in modulus (SMD 0.82; 95% CI 0.58–1.07), and small increases in CSA (SMD 0.22; 95% CI 0.12–0.33). Meta-regression revealed that the main moderator of increased stiffness was modulus. Resistance training interventions induced greater increases in modulus than other training types (SMD 0.90; 95% CI 0.65–1.15) and higher strain resistance training protocols induced greater increases in modulus (SMD 0.82; 95% CI 0.44–1.20; *p* = 0.009) and stiffness (SMD 1.04; 95% CI 0.65–1.43; *p* = 0.007) than low-strain protocols. The magnitude of stiffness and modulus differences were greater in adult participants.

**Conclusions:**

Mechanical loading leads to positive adaptation in lower limb tendon stiffness, modulus and CSA. Studies to date indicate that the main mechanism of increased tendon stiffness due to physical training is increased tendon modulus, and that resistance training performed at high compared to low localised tendon strains is associated with the greatest positive tendon adaptation.

PROSPERO registration no.: CRD42019141299.

**Supplementary Information:**

The online version contains supplementary material available at 10.1007/s40279-022-01695-y.

## Key Points

**Table:** 

Mechanical loading induced moderate increases in tendon stiffness, large increases in tendon modulus, and small increases in cross-sectional area
Changes in tendon modulus were the primary moderator of changes in stiffness
High-strain protocols induced larger increases in tendon stiffness than low-strain protocols

## Introduction

Tendons are viscoelastic structures that transmit forces from muscles to bones to enable movement. During stretch-shorten cycles, tendons store and release elastic energy [1, 2], which augments the power generated by the muscle–tendon unit (MTU) [3, 4], reduces the metabolic cost of muscle work [5], and may reduce the risk of muscle damage during active lengthening [6]. Tendon compliance also allows for a decoupling of fascicle length from MTU length changes (i.e., muscle gearing), which can modulate muscle force due to a dependency of muscle force on fibre length and speed [7, 8]. A large body of research over the past two decades has revealed that mechanosensitive tendons are highly responsive to changes in mechanical load, and can become stiffer and stronger with sustained increases in loading [9, 10]. These adaptations alter the mechanical function of the tendon, as a stiffer tendon experiences less strain at a given load and stores more energy at a given strain, and a stronger tendon resists a greater external load prior to failure. Stiffer lower limb tendons are associated with greater rates of force development [11–14], faster sprint times [15], improved running economy [5, 16, 17], and reduced risk of tendon damage [18]. Although there is some evidence that strain magnitude and duration are important factors [19–21], the mechanical loading stimulus across other loading dimensions (e.g., loading volume, rate, duty cycle) required to induce anabolic tendon adaptation is unclear. Consequently, determining the optimal training parameters for tendon adaptations is important for enhancing athletic performance and preventing injury.

Although the mechanobiological mechanisms underpinning training-induced tendon adaptations are not fully understood, mechanical loading clearly triggers a biological cascade that regulates structural tissue adaptation including collagen synthesis and turnover [22, 23]. Mechanical loading applied to the whole tendon is transmitted to the cell nucleus, causing an acute upregulation of cytokines and collagen-inducing growth factors (e.g., insulin-like growth factor), which results in secretion of new proteins into the extracellular matrix [24]. Evidence from in vitro [25–27] and in vivo [19–21] studies suggests that the strain magnitude and duration experienced by the tenocytes is the crucial mechanical stimulus for tendon adaptation. Indeed, it appears that an optimal strain dose may exist where the anabolic tendon remodelling is maximised [19, 20], with either too little or too much strain leading to catabolic adaptation [24]. It therefore follows that dose–response studies of tendon adaptation should, where possible, control the dose of tendon strain.

One of the challenges in understanding the dose–response relationship for in vivo human tendons is, with a few notable exceptions [19–21], that the direct mechanical loading dose applied to the tendon (i.e., strain) is rarely measured. Instead, the mechanical load is typically defined by the training type performed (e.g., resistance training, endurance training, jump training) and/or the relative loading intensity expressed as a percentage of one repetition maximum or maximal voluntary contraction. Although these external loading parameters have no fixed relationship with tendon strain, from a practitioner perspective, comparisons between training types and relative training intensities nevertheless provides insight into the training features most associated with positive tendon adaptation. In addition to training intensity, loading parameters such as load duration (repetitions × sets) and total load volume (relative intensity × duration) may also influence the extent and time course of tendon adaptation, and therefore also require investigation. Although it is acknowledged that load intensity and load duration may be independent stimuli for tendon adaptation, the total loading volume captures the overall effect of specific loading interventions on tendon adaptation. A further loading parameter that has been the subject of prior human in vivo tendon training studies is muscle contraction mode (e.g., concentric, eccentric, concentric:eccentric, isometric) which has been evaluated in two systematic reviews on tendon adaptation [9, 10]. Unlike strain magnitude, there is no compelling evidence to suggest contraction mode is an independent driver of tendon adaptation, but in line with earlier reviews, should be examined primarily for completeness and as important information for practitioners. An updated review of such studies in the context of the broader dose–response literature may give practitioners a clear evidence base for the training parameters likely to be most effective for promoting positive tendon adaptation.

Tendon properties also change across the lifespan. Maturation during childhood and adolescence leads to increased lower limb tendon CSA [28] and stiffness [29], whereas ageing is typically associated with reduced tendon stiffness and modulus [30]. Although older tendons appear to retain their mechanosensitivity [31], age-related declines in cell proliferation and collagen synthesis might influence the type or magnitude of training-induced tendon adaptation [22]. However, to date, no previous review has systematically synthesised the effect of age on mechanical loading-induced tendon adaptations.

The in vivo tendon stiffness response to altered mechanical loading can occur via changes in tendon morphology (e.g., increased cross-sectional area (CSA)), changes in material properties such as Young’s modulus (slope of the linear part of the stress–strain curve), or a combination of these factors. Tendon stiffness is commonly obtained from the slope of the force–deformation curve, with tendon forces typically obtained from the ratio of external joint moment and tendon moment arm, and tendon longitudinal deformation measured using B-mode ultrasound [32]. Reliable measures of in vivo tendon CSA have been reported using two- and three-dimensional ultrasound and magnetic resonance imaging (MRI) [33, 34], although the evidence for ultrasound assessment is mixed [35, 36]. Young’s modulus, which reflects the material properties of the tendon independent of tendon geometry, is obtained from the slope of the stress–strain curve, where stress is force normalised by CSA, and strain is deformation normalised to resting (slack) length.

In their systematic review of 27 studies investigating in vivo human tendon adaptation in response to mechanical loading, Bohm et al. [9] concluded that increased tendon stiffness was primarily explained by adaptation in material rather than morphological properties. In a similar review on the effects of increased mechanical loading on in vivo tendon properties (including ten cross-sectional studies), Wiesinger et al. [10] concluded that short-term adaptation of tendon stiffness was primarily due to increased tendon modulus and, based on observation from cross-sectional studies, that tendon hypertrophy may contribute to long-term increases in tendon stiffness. As a significant number of studies have been published on this topic since these prior systematic reviews, a contemporary synthesis may help to clarify which loading regimes result in the greatest adaptation and the extent to which training-induced changes in stiffness are driven by alterations in tendon material properties or morphology.

The purpose of this systematic review and meta-analysis was therefore to determine: (1) the overall effect of mechanical loading on tendon mechanical, material and morphological properties; (2) whether adaptations in stiffness are driven primarily by changes in CSA or modulus; (3) the effect of training type (i.e., aerobic training, resistance training, concurrent training or jump-based training) and associated loading parameters (relative intensity, localised strain, load duration, load volume and contraction mode) on the tendon mechanical, material and morphological properties; and (4) whether the magnitude of adaptation in tendon properties differs between age groups.

## Methods

This study was registered on the international Prospective Register of Systematic Reviews (PROSPERO 2019, CRD42019141299) and is compliant with the Preferred Reporting Items for Systematic Reviews and Meta-Analysis (PRISMA) guidelines [37].

### Search Strategy

A systematic search of the literature was conducted in PubMed, Scopus, EBSCOHost (CINAHL and SPORTDiscus) and EMBASE from database inception to 4 September 2021. The search terms included keywords and synonyms relating to tendon tissue location, mechanical loading including resistance training and endurance training, and tendon properties (see Online Supplemental Material (OSM) S1). Searches were limited to articles with human participants that were published in English, peer-reviewed and available in full-text. If the full-text version of an article was not available, the authors were contacted to source access, and all full-texts were made available for screening. Studies identified through searches were imported to Rayyan [38] and duplicates removed. Reference lists of included papers and previous systematic reviews [9, 10] were also inspected for further relevant titles.

### Study Selection

Titles and abstracts were initially screened by two independent reviewers (SLL and MNB) based on predetermined selection criteria (Table 1), with included studies progressing to full text review. Full text reviews were conducted by the same two independent reviewers (SLL and MNB) based on predetermined criteria (Table 1). Conflicts at either stage were resolved by consensus moderation. If consensus could not be reached, a decisive third reviewer (full text screen: DAO) was employed to resolve the conflict. Studies were required to report both pre- and post-intervention measurements of morphology (i.e., CSA), material (i.e., modulus) or mechanical (i.e., stiffness) properties of lower limb tendons, and were excluded if pre- and post-intervention data were unavailable.

**Table 1:** Study selection criteria

Criteria	Inclusion	Exclusion
Participants	Human Any age Any sex Uninjured/healthy participants with no systemic or long-term problems which would alter tendon health and/or adaptation to exercise	Animal Participants with tendon-related injury (e.g., tendinopathies, direct lesions, ruptures) or post-surgical tendons (e.g., grafts, tendon transfers) Any long-term issue which would affect tendon health and/or adaptation to exercise, e.g., cerebral palsy
Paper type	Original research articles	Reviews
		Editorials/commentaries/opinion papers
		Conference abstracts/papers
Study design	Experimental designs, e.g., RCT	Descriptive designs, e.g., cross-sectional
Outcome measures	Morphology/geometry (i.e., CSA)	Other tendon-related outcome measures
	Material properties (i.e., modulus)	
	Mechanical properties (i.e., stiffness)	
Protocol	Lower limb exercise interventions, including: targeted resistance training; stretch–shortening activity (e.g., jump or plyometric); endurance training (e.g., running, cycling) ≥ 2 weeks in duration Two or more supervised resistance training sessions Loading parameters (repetitions, sets, or duration/time under tension) detailed	Periods of immobilisation prior to loading Unsupervised training or home programmes with training diaries only < 2 weeks duration No full description of training intervention

### Study Quality Assessment

Included studies were assessed using the Physiotherapy Evidence Database (PEDro) scale. Study quality for each article was assessed by two reviewers (SLL and MNB or SJD) with conflicts resolved by consensus. Where conflicts could not be resolved, the assessment of a third reviewer (DAO) was used. Due to the nature of exercise-based trials, it was not possible to blind participants or the supervising therapist(s) to the presence of exercise or a specific exercise-type during the study. As a consequence, criteria five and six could not be met for any of the included studies and so the maximum score for randomised controlled trials and non-controlled experimental designs was eight [39]. Further, for single-group study designs, all criteria (items two, three, four and ten) that make reference to multiple groups were not applicable. As such, the maximum score for a single-group design was four. The relative score was presented as a percentage of the total score divided by the total possible score for each design as per a previous review [39]. Funnel plots were created and Egger’s test was used to provide an estimation of small study bias for each of the three main outcome measures. Where Egger’s test indicated statistically significant bias, we used a selection model [40] to compute an adjusted SMD to ensure that our conclusions were robust. These adjusted SMDs, and details of the selection model, are provided in the OSM (S2–S4). All included papers were retained for analysis. Inter-rater agreement on the PEDro criteria and the aforementioned full-text screening were expressed using Cohen’s Kappa using the ‘irr’ package [41].

### Data Extraction

Data extraction was conducted by one reviewer (SLL) employing Microsoft Excel. Study characteristics extracted were: participant information (age, sex, physical characteristics); tissue assessed (AT, patellar tendon (PT), quadriceps tendon (QT), vastus lateralis (VL) aponeurosis); intervention type and loading parameters (exercise/activity used, contraction type, repetitions, sets, load or strain magnitude, contraction durations, training volume, intervention duration); and pre- and post-intervention measures of CSA, modulus or stiffness properties. No control group data were extracted due to the heterogeneity between control groups. Description of control groups or non-included intervention groups is described in the OSM (S5). Where studies performed interventions on single limbs (either between- or within-participant), these were extracted and analysed as independent groups. Articles were separated into high (≥ 70%) or low (< 70%) intensity protocols and < 12 weeks’ or ≥ 12 weeks’ duration in line with a previous systematic review investigating tendon adaptation to mechanical loading [9]. Contraction mode sub-groups were separated into concentric-only protocols (concentric), eccentric-only (eccentric), isometric or traditional contractions modes encompassing both concentric and eccentric phases (Con:Ecc). Volume in arbitrary units (au; repetitions × sets × relative intensity [10]) was categorised as high (> 3,200 au) or low (< 3,200 au) based on the median of all articles that provided sufficient detail to calculate this. Studies that included hop-, jump- or plyometric-based protocols were included and classified as jump-based training. Central tendency and variation data from pre- and post-intervention measures for each available outcome measure were also extracted. Where variance was presented as standard error, this was converted to standard deviation (SD) using the method provided in the Cochrane Handbook (Sect. 6.5.2.2) [42]. Where required data were only presented graphically, rather than tabulated or documented in-text, data were extracted manually from figures using GetData Graph Digitizer (version 2.24). If studies did not report sufficient detail for outcome measures (e.g., only reported percentage change with no pre-intervention measures, absent central tendency or variation data), authors were contacted by email to request clarification or additional data. If no reply was received within 2 weeks, a follow-up email was sent. If no contact was received after a further 2 weeks, the studies were excluded if there were no usable data from the article. Where studies did not report sufficient protocol information related to sub-groupings, for example, protocol intensity, the affected intervention group was excluded from that sub-group analysis only but retained for analysis elsewhere.

### Data Processing and Analysis

All data analysis was conducted in R [43]. Three separate meta-analyses were performed: (1) studies that assessed exercise-induced changes in stiffness, modulus or CSA; (2) studies of different training types (aerobic, concurrent, resistance training, jump-based training); and (3) resistance-training studies only to investigate differential effects of age, contraction mode, duration, intensity, strain and volume. Meta-analyses were conducted using the ‘meta’ [44] and ‘metafor’ [45] packages.

Standardised mean differences (SMD) with 95% confidence intervals (CI) were calculated for each analysis from each group’s pre- and post-intervention mean and standard deviation (SD) using Hedges’ *g*. In other words, the SMD represents the standardized difference between the pre- and post-intervention scores. SMD were described as trivial (< 0.20), small (0.20–0.49), moderate (0.50–0.79) or large (≥ 0.80) [46]. Data were pooled using a random effects model, using the DerSimonian and Laird method to estimate variance. The percentage of variation across studies that is due to heterogeneity rather than sampling error was expressed using the I^2^ statistic. To minimise intervention heterogeneity for the sub-group analyses of age, training duration and contraction mode, only high-intensity training protocols (≥ 70% 1RM/MVC) were included [9]. Where possible, sub-group analyses also included results for matched studies, i.e., those that included more than one intervention group and manipulated the training variable of interest while matching other potentially confounding variables. Data for all lower limb tendons were aggregated based on the conclusion by Wiesinger et al. that tendon adaptations were comparable between tendons [10]. If papers reported values from a single site or already included a mean for multiple sites, these values were used in the meta-analysis. Where only sub-regions (e.g., distal, mid-tendon, proximal or specified intervals) were reported for CSA, pooled mean and SD were calculated using the formulae in the Cochrane Handbook (Table 6.5.a [9, 42]).

When significant effects (*p* < 0.05) were indicated by the random effects model, a post hoc Wald-type test was used to assess pairwise comparisons using the ‘metafor’ (45) package with alpha set at 0.05 to establish differences in the magnitude of adaptations between sub-groups. This was presented as *χ*^2^.

Meta-regression was used to assess whether differences in pre- and post-intervention stiffness were moderated by differences in modulus and/or CSA. To improve interpretability, the effect size for this analysis was computed using the log-transformed ratio of means [47], and then back-transformed and expressed as a percentage.

## Results

### Yield and Study Characteristics

**Fig. 1:**
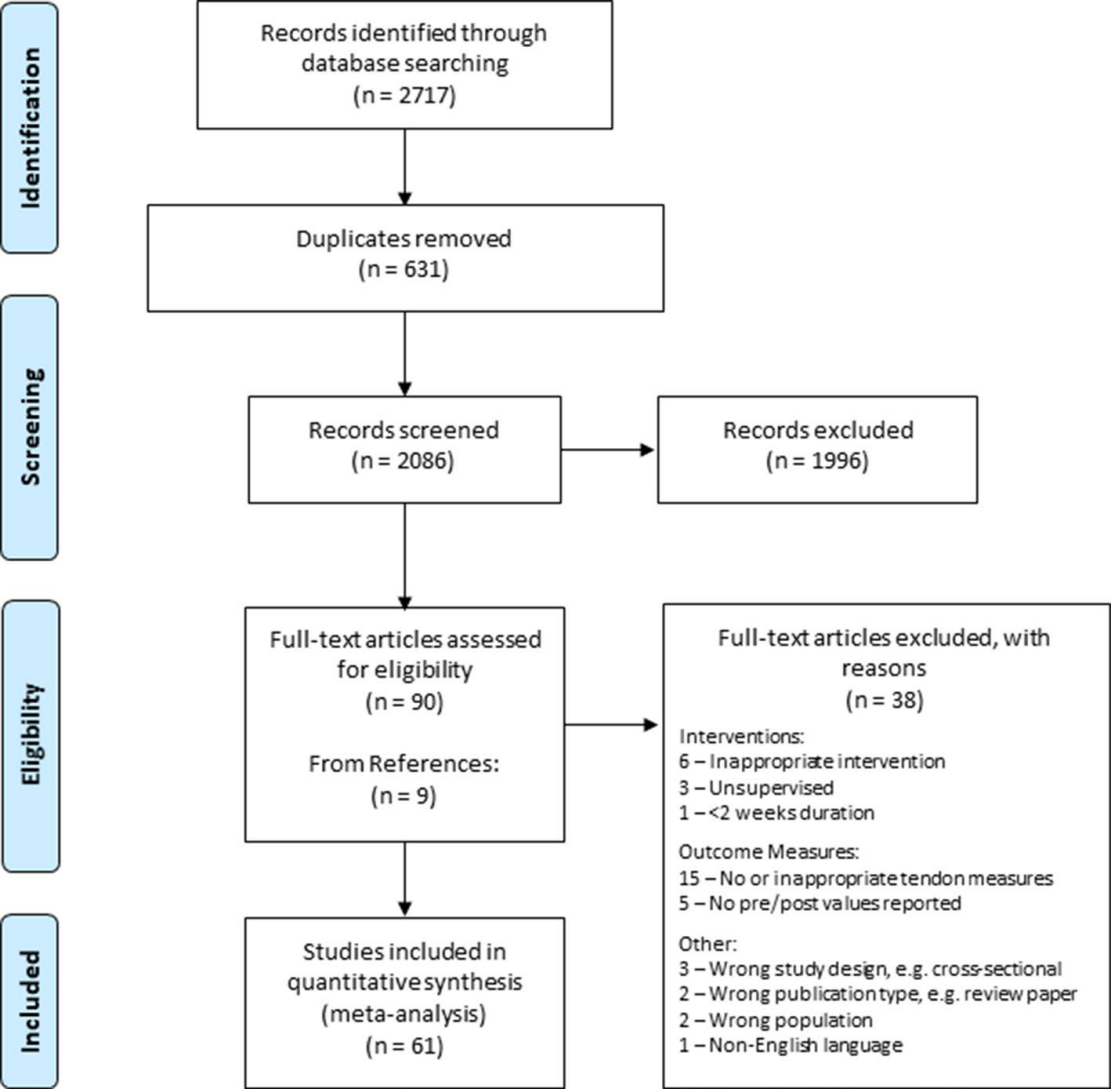
PRISMA flow diagram

Following the systematic search and screening processes (Fig. 1), a total of 61 articles were included in this review. Reviewer agreement was assessed using Cohen’s kappa and demonstrated very good agreement (*k* = 0.89) for full-text screening [48]. Thirty-two studies investigated AT only, 18 investigated PT only, two investigated the VL aponeurosis, and nine investigated multiple tendons (PT and VL aponeurosis = 5, QT and VL aponeurosis = 3, PT and AT = 1). Seven studies reported morphological measures only, 15 reported mechanical measures only, and 39 reported two or more measures. Twenty studies reported all three outcome measures and were included in the meta-regression analysis. Of the studies that reported morphological measures, 24 used MRI, 20 used two-dimensional/B-mode ultrasound and two used three-dimensional ultrasound. All studies that measured tendon stiffness used isometric maximal voluntary contractions, with 42 studies employing isokinetic dynamometry, seven using strain gauges or load cells, one using a ‘myometer’ and one using an ‘ergometer’. This was paired with two-dimensional ultrasound assessment of tendon elongation at associated musculotendinous junctions (20 studies), displacement at tendon insertions (14 studies), displacement of fascicles at an associated aponeurosis (13 studies), or a combination of these (four studies). Study characteristics are summarised in Table 2 with extended descriptions of exercise parameters and measurement methods in the OSM (S5).

**Table 2:** Study characteristics

Source	Participants					Intervention		Tendon			
Author	Group	*N* (M/F)	Age (years)	Height (cm)	Mass (kg)	Duration (weeks), Frequency (n x p/wk)	Group exercise characteristics (intensity, volume, contraction/exercise type)	Stiffness	Modulus	CSA	Tissue
Albracht et al., 2013 [5]	Exercise	13 (M/F n/s)	27 ± 5	180 ± 6	76 ± 7	14, 4	High intensity, low volume iso	✔	✖	✖	AT
Arampatzis et al., 2007 [19]	Low strain limb High strain limb	3/8	29.5 ± 5.0	172 ± 5	64.1 ± 5.0	14, 4	Low strain/intensity, low volume iso vs. high strain/intensity, low volume iso	✔	✔	✔	AT
Arampatzis et al., 2010 [20]	Low strain limb High strain limb	11/0	23.9 ± 2.2	178 ± 5	77.2 ± 4.1	14, 4	Low strain/intensity, high volume iso vs. high strain/intensity, high volume iso	✔	✔	✔	AT
Baptista et al., 2016 [95]	Con limb Ecc limb	23/0	62.74 ± 2.2	172.02 ± 6.11	80.21 ± 14.82	12, 2	High intensity, low volume con vs. high intensity, low volume ecc	✖	✖	✔	PT
Bohm et al., 2014 [21]	High strain rate & Reference	14/0	26.7 ± 4.2	182.3 ± 5.3	82.2 ± 13.1	14, 4	High strain/intensity SSC vs. high strain/intensity iso + reference limb high intensity, low volume iso	✔	✔	✔	AT
	Long strain duration & Reference	12/0	29.5 ± 3.0	177.6 ± 7.0	74.8 ± 7.3						
Bohm et al., 2021 [17]	Intervention	9/4	29 ± 5	178 ± 8	73 ± 8	14, 3–4	High intensity, low volume iso	✔	✖	✖	AT
Carroll et al., 2011 [62]	Placebo	8/4	67 ± 2	170 ± 3	77.3 ± 4.4	12, 3	High intensity, low volume con:ecc	✔	✔	✔	PT
Centner et al., 2019 [71]	Heavy load	14/0	26.1 ± 4.2	179.7 ± 9.2	76.4 ± 15.4	14, 3	High intensity, low volume con:ecc	✔	✔	✔	AT
Dalgaard et al., 2019 [104]	Non-contraceptive	0/14	24 ± 1	167 ± 2	66.7 ± 2.2	10, 3	Variable intensity, variable volume con:ecc	✖	✖	✔	PT
Duclay et al., 2009 [105]	Ecc training	10/0	23 ± 3	178 ± 8	73 ± 7	7, 3	High intensity, high volume ecc	✔	✖	✖	AT
Eriksen et al., 2018 [63]	Old heavy	5/4	69 ± 2.2	170 ± 10	77.2 ± 15.2	12, 3	High intensity, low volume con:ecc	✔	✔	✔	PT
	Very old heavy	8/4	88 ± 3.7	171 ± 11	70.5 ± 13.5						
Eriksen et al., 2019 [64]	Heavy resistance	7/3	67 ± 2.3	171 ± 8	79 ± 14	52, 3	Variable intensity, variable volume con:ecc	✔	✔	✔	PT
Farup et al., 2014 [106]	Placebo Con	11/0	24.1 ± 0.9	181.0 ± 1.7	77.9 ± 2.2	12, 3	Variable intensity, variable volume con:ecc	✖	✖	✔	PT
	Placebo Ecc										
Fletcher et al., 2010 [60]	Iso	6/0	22.2 ± 3.1	180 ± 6	68.2 ± 8.3	8, 3	High intensity, low volume iso + intensity n/s R	✔	✖	✖	AT
Fouré et al., 2009 [107]	Training/Jump	9/0	18.8 ± 0.9	179.2 ± 6.1	68.5 ± 7.1	8, 2	Variable intensity, variable volume SSC	✔	✖	✖	AT
Fouré et al., 2010 [49]	Training/Jump	9/0	18.8 ± 0.9	177.3 ± 6.2	68.4 ± 6.5	14, n/s	Variable intensity, variable volume SSC	✔	✖	✔	AT
Fouré et al., 2011 [50]	Training/Jump	9/0	18.8 ± 0.9	177.3 ± 6.2	68.4 ± 6.5	14, n/s	Variable intensity, variable volume SSC	✖	✖	✔	AT
Fouré et al., 2012 [51]	Jump	9/0	19.6 ± 1.8	177.3 ± 6.2	68.1 ± 6.4	14, n/s	Variable intensity, variable volume SSC	✖	✖	✔	AT
Fouré et al., 2013 [108]	Ecc	11/0	21.2 ± 2.7	177.1 ± 6.1	71.1 ± 5.8	14, n/s	Variable intensity, variable volume 34 sessions total ecc	✔	✖	✔	AT
Geremia et al., 2018 [72]	Ecc	15/0	26 ± 5	176 ± 7	75 ± 9	12, 2	High intensity, high volume ecc	✔	✔	✔	AT
Hirayama et al., 2017 [109]	Training	11/0	22 ± 3	172.0 ± 5.8	66.9 ± 10.5	12, 3	High intensity, high volume SSC	✔	✖	✖	AT
Houghton et al., 2013 [73]	Plyometric	7/0	21 ± 4	174.6 ± 3.1	73.7 ± 10.3	8, 2	Variable intensity SSC	✔	✔	✔	AT
Kay et al., 2016 [110]	Training	13/0	20.0 ± 0.9	180 ± 10	75.9 ± 8.5	6, 2	High intensity ecc	✔	✖	✖	AT
Kongsgaard et al., 2007 [65]	Heavy Resistance	12/0	24.6 ± 1	183 ± 2	80.9 ± 3.9	12, 3	High intensity, high volume con:ecc vs. low intensity con:ecc	✔	✔	✔	PT
	Light Resistance										
Kubo et al., 2001 [51]	Short duration	8/0	22.6 ± 2.8	171.5 ± 6.1	69.2 ± 5.8	12, 4	High intensity, high volume iso vs. high intensity, low volume iso	✔	✖	✔	QT VL apon
	Long duration										
Kubo et al., 2001 [52]	Iso	8/0	22.6 ± 2.8	171.5 ± 6.1	69.2 ± 5.8	12, 4	High intensity iso	✔	✔	✔	QT VL Apon
Kubo et al., 2002 [111]	Resistance Training	8/0	21 ± 2	172 ± 4	64 ± 6	8, 4	High intensity, low volume con:ecc	✔	✖	✔	AT
Kubo et al., 2006 [112]	Iso	14/0	20 ± 1	168 ± 4	60 ± 2	12, 4	High intensity, low volume iso	✔	✖	✔	PT VL Apon
Kubo et al., 2006 [113]	Short length	9/0	24 ± 1	172 ± 6	70 ± 9	12, 4	High intensity, high volume iso vs. high intensity, high volume iso	✔	✖	✔	QT VL Apon
	Long length										
Kubo et al., 2006 [96]	High load	9/0	24 ± 1	172 ± 4	73 ± 13	12, 3	High intensity con:ecc	✔	✖	✔	PT VL Apon
Kubo et al., 2007 [85]	Plyometric/ Jump	10/0	22 ± 2	170 ± 3	63 ± 8	12, 4	Low intensity SSC vs. high intensity, high volume con:ecc	✔	✖	✔	AT
	Weight training										
Kubo et al., 2009 [114]	Iso	10/0	22.3 ± 1.1	171.4 ± 6.1	63.8 ± 8.7	12, 4	High intensity, low volume iso vs. high intensity, high volume con:ecc	✔	✖	✔	PT VL Apon
	Con:Ecc										
Kubo et al., 2010 [115]	Knee extension	10/0	22.3 ± 1.1	171.4 ± 6.1	63.8 ± 8.7	12, 4	High intensity con:ecc vs. high intensity con:ecc	✔	✖	✔	PT AT
	Plantar flexion	10/0	22.5 ± 1.6	169.8 ± 3.3	62.7 ± 7.8						
Kubo et al., 2010 [88]	Iso	8/0	22.0 ± 0.8	171.2 ± 6.7	62.6 ± 9.3	12, 4	High intensity iso	✔	✖	✔	PT VL Apon
Kubo et al., 2012 [89]	Iso	9/0	23.4 ± 0.6	174.5 ± 2.0	69.1 ± 3.2	12, 4	High intensity, low volume iso	✔	✖	✔	AT
Kubo et al., 2017 [86]	Iso	11/0	22.5 ± 3.2	172.2 ± 2.5	60.9 ± 6.5	12, 3	High intensity, low volume iso vs. low intensity SSC	✔	✖	✔	AT
	Plyometric										
Kubo et al., 2017 [116]	Con	9/0	20.8 ± 0.5	173.6 ± 5.5	65.6 ± 7.7	12, 3	High intensity, high volume con vs. high intensity, high volume ecc	✔	✖	✔	PT
	Ecc										
Laurent et al., 2020 [117]	Knee extended	6/5	n/s (adult) n/s (adult)	180.5 ± 5.8	68.7 ± 14	10, 2	Intensity n/s, variable volume SSC	✔	✖	✔	AT
	Knee flexed	6/5		180.9 ± 10.5	69.7 ± 10.8						
Malliaras et al., 2013 [61]	Con	9/0	29 ± 5.1	179 ± 9.0	79.0 ± 14.5	12, 3	High intensity con vs. high intensity ecc vs. high intensity ecc	✔	✔	✔	PT
	Ecc	10/0	28 ± 4.6	179 ± 7.5	76.0 ± 12						
	High load ecc	10/0	27 ± 3.8	177 ± 8.3	75.0 ± 6.3						
Massey et al., 2018 [66]	Explosive	14/0	25 ± 2	174 ± 7	71 ± 10	12, 3	High intensity iso vs. high intensity iso	✔	✔	✔	PT VL apon
	Sustained	15/0	25 ± 2	175 ± 8	70 ± 8						
McMahon et al., 2013 [67]	Short range	6/4	19 ± 2.2	176 ± 15	75.7 ± 3.2	8, 3	High intensity, variable volume con:ecc vs. Low intensity, variable volume con:ecc vs. High intensity, variable volume con:ecc	✔	✔	✔	PT
	Long range	5/6	21 ± 3.4	175 ± 14	74.9 ± 14.7						
	Full range	7/4	19 ± 2.6	171 ± 11	73.8 ± 14.9						
McMahon et al., 2018 [68]	Trained males	8/0	20 ± 1	n/s	81 ± 4	8, 3	High intensity con:ecc	✔	✔	✔	PT
	Trained females	0/8	19 ± 3	n/s	69 ± 3						
Mouraux et al., 2000 [118]	Ecc	6/4	24.7 ± 3.2	n/s	n/s	6, 3	Variable intensity, variable volume ecc	✖	✖	✔	AT
Ogiso et al., 2020 [119]	Non-muscle stimulation	9/0	19.2 ± 0.8	169 ± 5	61.0 ± 3.5	3, 3	High intensity, high volume SSC	✔	✖	✖	AT
Onambélé et al., 2008 [120]	Resistance training	6/6	70.2 ± 1.5	n/s	n/s	12, 3	High intensity, variable volume con:ecc vs. high intensity variable volume con:ecc	✔	✖	✖	AT
	Inertial flywheel training	6/6	69.6 ± 1.1	n/s	n/s						
Quinlan et al., 2021 [121]	Young con Young ecc Old con Old ecc	10/0 10/0 8/0 9/0	23.3 ± 3.8 25.3 ± 6.2 69.1 ± 3.0 67.5 ± 1.5	175 ± 1.3 176 ± 2.2 175 ± 3.3 176 ± 5.8	74.2 ± 4.4 73.0 ± 6.1 79.2 ± 8.5 76.8 ± 10.4	8, 3	Low intensity, high volume con vs. low intensity high volume ecc	✔	✔	✔	PT
Reeves et al., 2003 [69]	Training	4/5	74.3 ± 3.5	163.4 ± 9.1	69.7 ± 14.8	14, 3	Variable intensity, variable volume	✔	✔	✔	PT
Reeves et al., 2003 [122]	Training	3/4	73.6 ± 3.4	162 ± 10.7	69.4 ± 17.7	14, 3	Variable intensity, variable volume	✔	✖	✖	PT
Sanz-López et al., 2016 [123]	Ecc overload	10/0	22.8 ± 4.2	179.8 ± 7.9	72.6 ± 6.7	6, 2	High intensity con:ecc	✖	✖	✔	AT
Seynnes et al., 2009 [70]	Training	15/0	20.4 ± 2.2	177 ± 4	73.6 ± 6.3	9, 3	High intensity con:ecc	✔	✔	✔	PT
Standley et al., 2013 [124]	Aer cyc	0/9	70 ± 2	165 ± 2	67.2 ± 4.1	12, 3–4	Variable intensity, variable volume Aer (cyc)	✖	✖	✔	PT
Tillin et al., 2012 [125]	Trained limb	10/0	20 ± 2	182 ± 7	74 ± 7	4, 4	High intensity, high volume iso	✔	✖	✖	VL Apon
Vikmoen et al., 2016 [59]	Endurance + strength	0/11	31.5 ± 8.0	169 ± 5	62.4 ± 5.2	11, 2	Cc, variable intensity, variable volume con:ecc + Cyc/R	✔	✔	✔	PT
Wakahara et al., 2015 [75]	Training	11/0	26.5 ± 2.0	173.4 ± 4.6	67.9 ± 7.4	12, 3	High intensity, high volume con:ecc	✖	✖	(Width)	VL apon
Walker et al., 2020 [58]	Traditional training	10/0	21 ± 2	178 ± 7	78 ± 12	10, 2	Variable intensity, variable volume con:ecc vs. variable intensity, variable volume con:ecc	✔	✖	✖	PT
	Accentuated ecc training	10/0	21 ± 2	179 ± 8	76 ± 11						
Waugh et al., 2014 [74]	Training	5/5	8.9 ± 0.2	136.3 ± 5.6	28.4 ± 4.7	10, 2	Variable intensity, variable volume	✔	✔	✔	AT
Waugh et al., 2018 [54]	Long rest	7/7	M: 30.1 ± 7.9	M: 181.9 ± 5.8	M: 83.2 ± 6.0	12, 3	High intensity, high volume iso vs. high intensity, high volume iso	✔	✔	✔	AT
	Short rest		F: 29.9 ± 5.2	F: 164.5 ± 7.5	F: 61.3 ± 7.2						
Waugh et al., 2021 [55]	Long rest	7/7	M: 30.1 ± 7.9	M: 181.9 ± 5.8	M: 83.2 ± 6.0	12, 3	High intensity, high volume iso vs. high intensity, high volume iso	✔	✔	✔	AT
	Short rest		F: 29.9 ± 5.2	F: 164.5 ± 7.5	F: 61.3 ± 7.2						
Werkhausen et al., 2018 [56]	Isometric	5/6	26 ± 4	174 ± 9	70 ± 9	10, 3	High intensity, high volume iso	✔	✖	✖	AT
Werkhausen et al., 2019 [57]	Training	5/6	26	174	70	10, 3	High intensity, high volume iso	✔	✖	✖	AT
Wu et al., 2010 [126]	Training/Jump	11/0	22.1 ± 1.6	174.4 ± 7.6	65.8 ± 8.6	8, 2	Variable intensity, variable volume	✔	✖	✖	AT

Aer, aerobic; AT, Achilles tendon; Cc, concurrent training; Con, concentric; Con:Ecc, concentric:eccentric; Cyc, cycling; DF, dorsiflexion; Ecc, eccentric; CMJ, countermovement jump; DJ, drop jump; F, female; GaM Apon, Gastronemius medialis aponeurosis; GRF, ground reaction force; Iso, isometric; M, Male; n/s, not specified; min, minute; MVC, maximal voluntary contraction; PF, plantarflexion; PT, patellar tendon; QT, Quadriceps tendon; R, running; rpm, revolutions per minute; SJ = squat jump; SSC, stretch shortening cycle; VL Apon, Vastus lateralis aponeurosis; vs., versus; Wk, week(s); 1RM, one repetition maximum; 5RM, five repetition maximum; n x p/wk, number of sessions per week

NB. Relative volume = relative intensity × repetitions × sets, and is expressed as ‘high’ or ‘low’ based on median. Full exercise parameters and prescription available in Online Resource S5

Three papers from Fouré et al. reported duplicate data for AT CSA and two reported duplicate stiffness measures [49–51]. Two papers from Kubo et al. reported duplicate data for QT CSA and VL aponeurosis stiffness [52, 53]. Two papers from Waugh et al. [54, 55] and Werkhausen et al. [56, 57] reported duplicate data for Achilles stiffness. Duplicates were confirmed through identical outcome measure values and corresponding participant characteristic information including anthropometrics. As such, the relevant values were only included once in the meta-analysis.

### Participant Characteristics

The total number of unique participants in eligible groups was 763 (male = 615, female = 135, not specified = 13). One study investigated tendinous tissue adaptations in children, seven studies examined elderly participants (> 60 years), one study included adult and elderly participants, and all others investigated adults. Participant characteristics for child (< 18 years), adult (18–59 years) and elderly (≥ 60 years) groups are displayed in Table 3. Participants were described in their respective studies as being a variation of “physically active”, “not involved in structured resistance training”, or “untrained”. One paper contained participants who regularly performed resistance training for the lower limb [58], one involved female endurance athletes [59], and one recruited highly trained runners [60].

**Table 3:** Pooled mean ± standard deviation for the main participant characteristics

	N	Age (years)	Height (cm)	Mass (kg)
Child	10	8.9 ± 0.2	136.3 ± 5.6	28.4 ± 4.7
Adult	629	23.7 ± 4.5	175.5 ± 7.8	71.1 ± 10.0
Elderly	124	70.0 ± 7.0	170.0 ± 7.8	75.4 ± 13.0

### Protocol/Intervention Characteristics

Forty-six studies included resistance training only, nine included jump-based training only, three studies included comparisons of resistance training and jump-based training, one included an aerobic (stationary cycling) intervention, and two performed concurrent resistance and aerobic training. Of the intervention groups included in the studies featuring resistance training, 22 interventions involved conventional protocols (concentric and eccentric contraction modes combined), 19 groups underwent isometric-only protocols, nine were eccentric-only, and four were concentric-only.

### Study Quality Assessment

Study quality scores ranged from 1 to 8 of a revised total of 8, with a median score of 4 (interquartile range (IQR): 3–6; OSM S6), and a median adjusted percentage of 50% (IQR: 38–75%). Only one paper met all relevant criteria [61], and 44% of the 61 included papers achieved an adjusted score of 60% or higher, representing a moderate-to-high methodological quality. The most common sources of potential methodological bias were: (1) not specifying the method of randomisation and concealment (7% of eligible studies met criteria), (2) not blinding the assessor to key outcomes (18% of eligible studies met criteria), (3) not reporting key outcomes for ≥ 85% of participants (41% of eligible studies met criteria), (4) failure to specify that all participants received the intervention (i.e., performed the training protocol) or were included in an intention to treat analysis (48% of eligible studies met criteria), and (5) inadequate reporting of between-group statistical differences (64% of eligible studies met criteria). Agreement between independent reviewers was good (*k* = 0.77) for individual criteria [48]. Complete agreement was achieved after consensus moderation.

### Meta-Analysis of All Included Studies

**Fig. 2:**
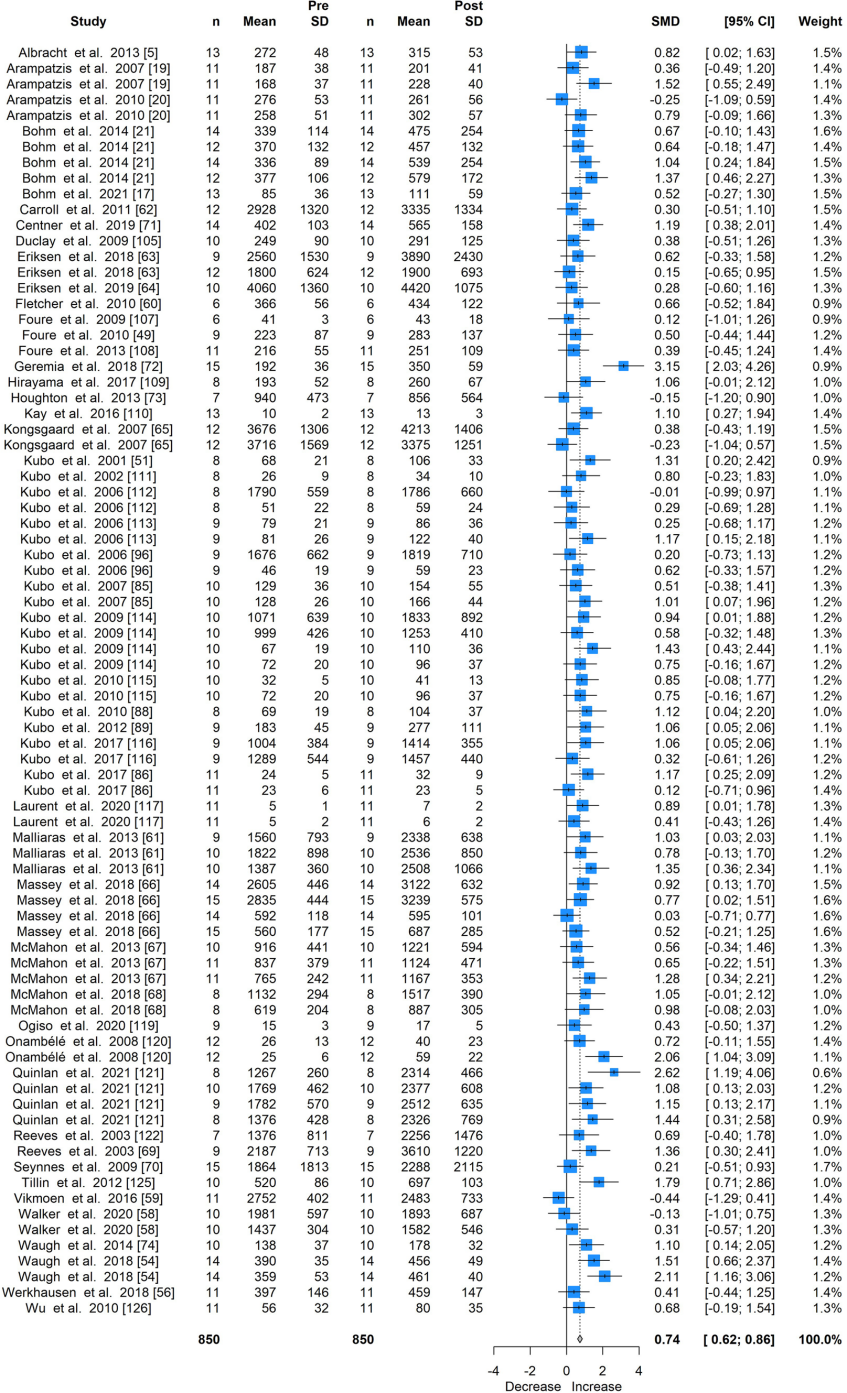
Forest plot for the meta-analysis of all studies providing stiffness measures, showing standardised mean differences (SMD) and 95% confidence intervals (CI)

**Fig. 3:**
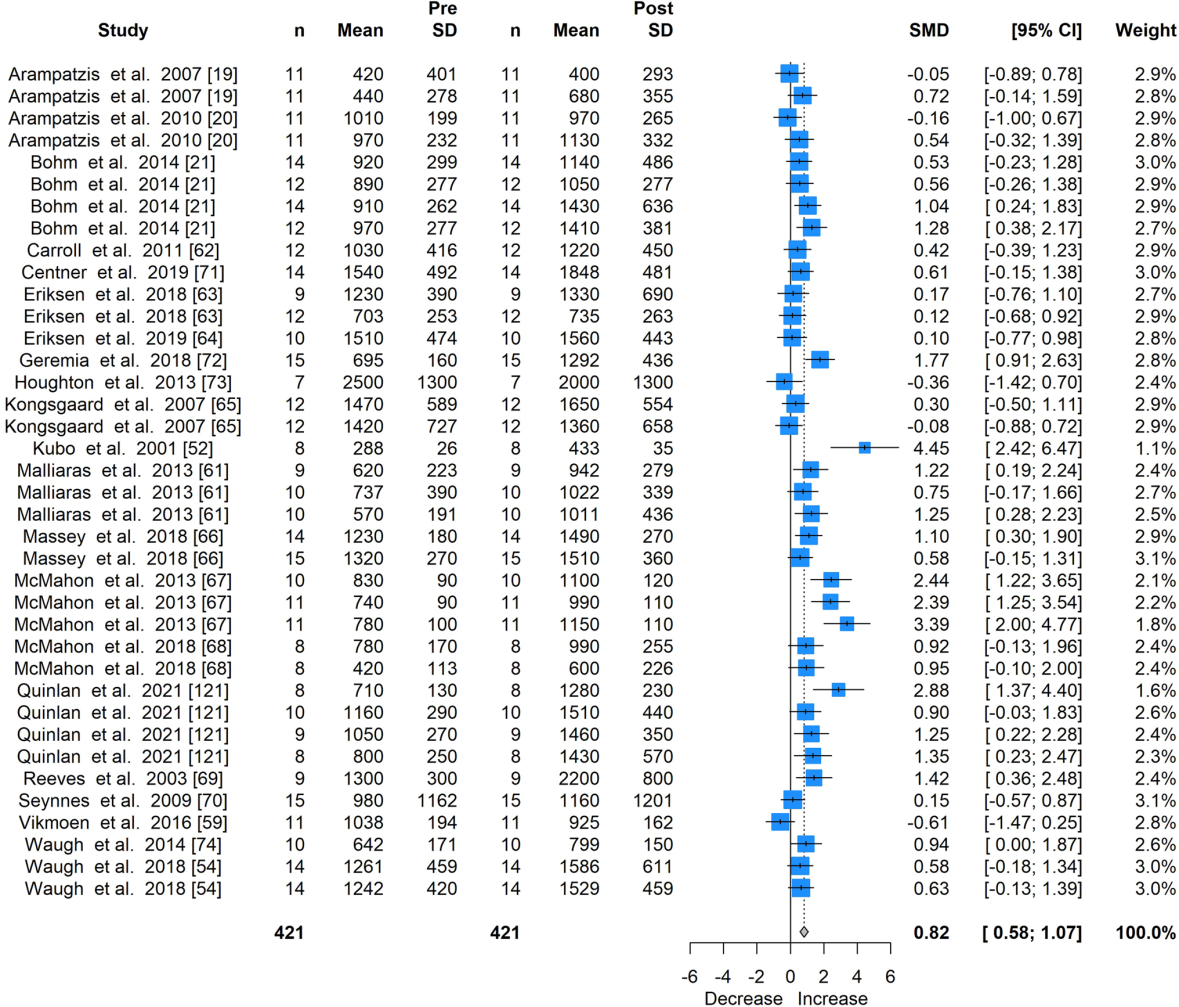
Forest plot for the meta-analysis of all studies providing elastic modulus measures, showing standardised mean differences (SMD) and 95% confidence intervals (CI) of all studies

**Fig. 4:**
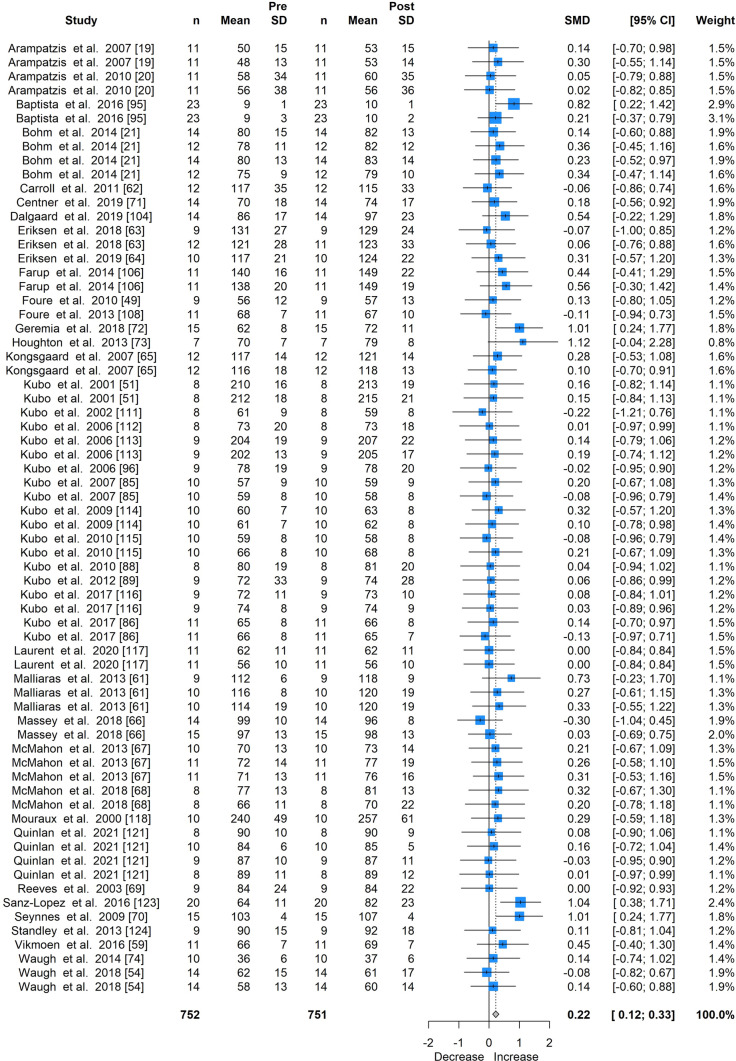
Forest plot for the meta-analysis of all studies providing cross-sectional area measures, showing standardised mean differences (SMD) and 95% confidence intervals (CI) of all studies

Mechanical loading was associated with a moderate increase in tendon stiffness (SMD 0.74; 95% CI 0.62–0.86; Fig. 2; 81 intervention groups), a large increase in modulus (SMD 0.82; 95% CI 0.58–1.07; Fig. 3; 38 intervention groups), and a small increase in tendon CSA (SMD 0.22; 95% CI 0.12–0.33; Fig. 4; 68 intervention groups).

### Meta-Regression Analysis

**Fig. 5:**
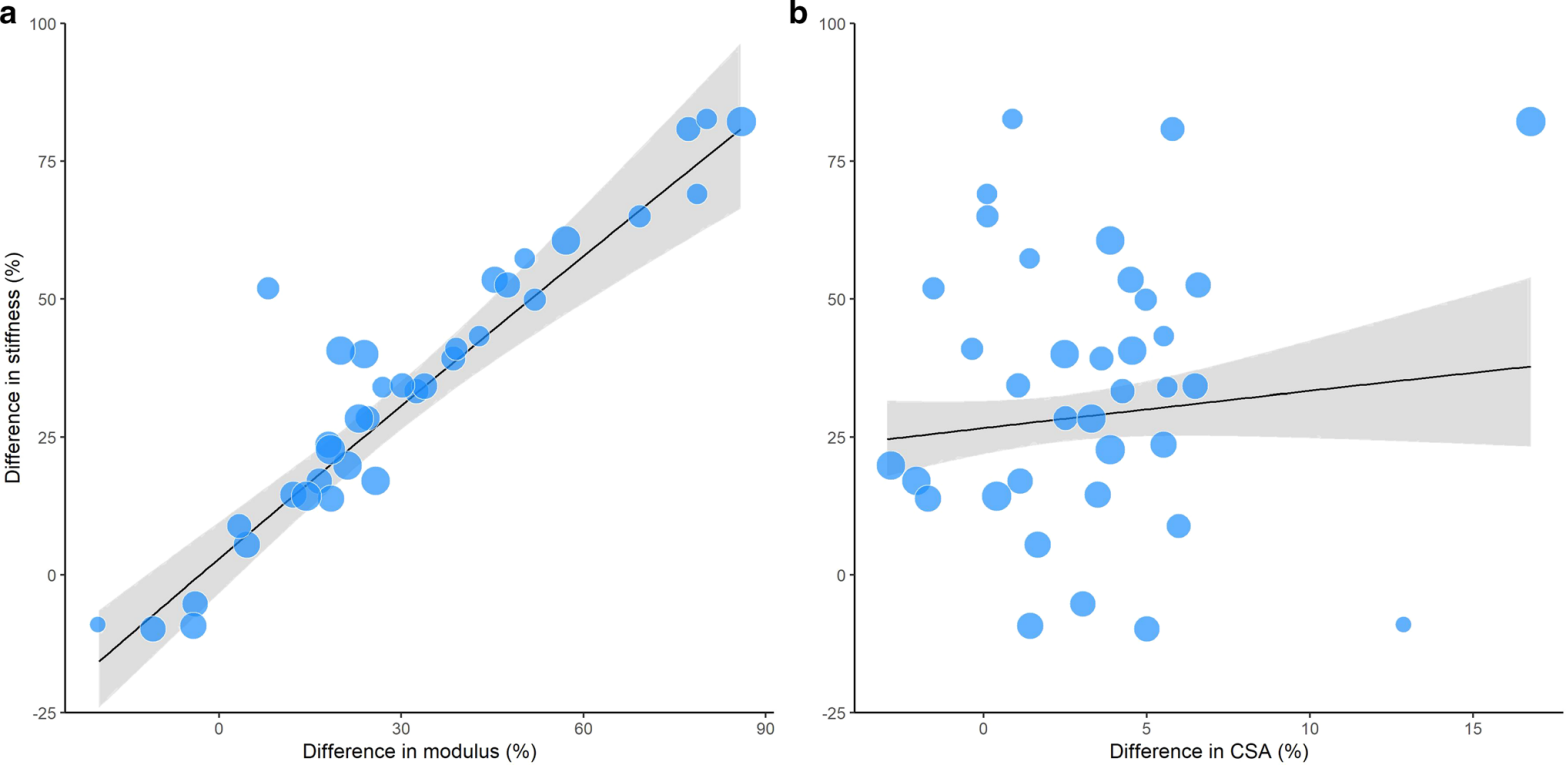
Bubble plot visualisation of meta-regression between the pre- and post-intervention percentage difference in stiffness increases versus **a** pre- and post-intervention percentage difference in modulus, and **b** pre- and post-intervention percentage difference in cross-sectional area (CSA). Only studies that concurrently measured stiffness, modulus and CSA were included in the meta-regression. The size of each bubble is proportional to the sample size of the included intervention groups. The black line represents the regression line of best fit. Grey-shaded area represents the 95% confidence intervals of the regression line

Meta-regression analysis of 20 studies [20, 21, 52, 54, 59, 61–74] that concurrently assessed tendon stiffness, modulus and CSA revealed that increased modulus was the predominant moderator of increased tendon stiffness (*p* < 0.001; Fig. 5). CSA was not a significant moderator of tendon stiffness (*p* = 0.19).

### Effect of Training Type on Tendon Adaptation

**Fig. 6:**
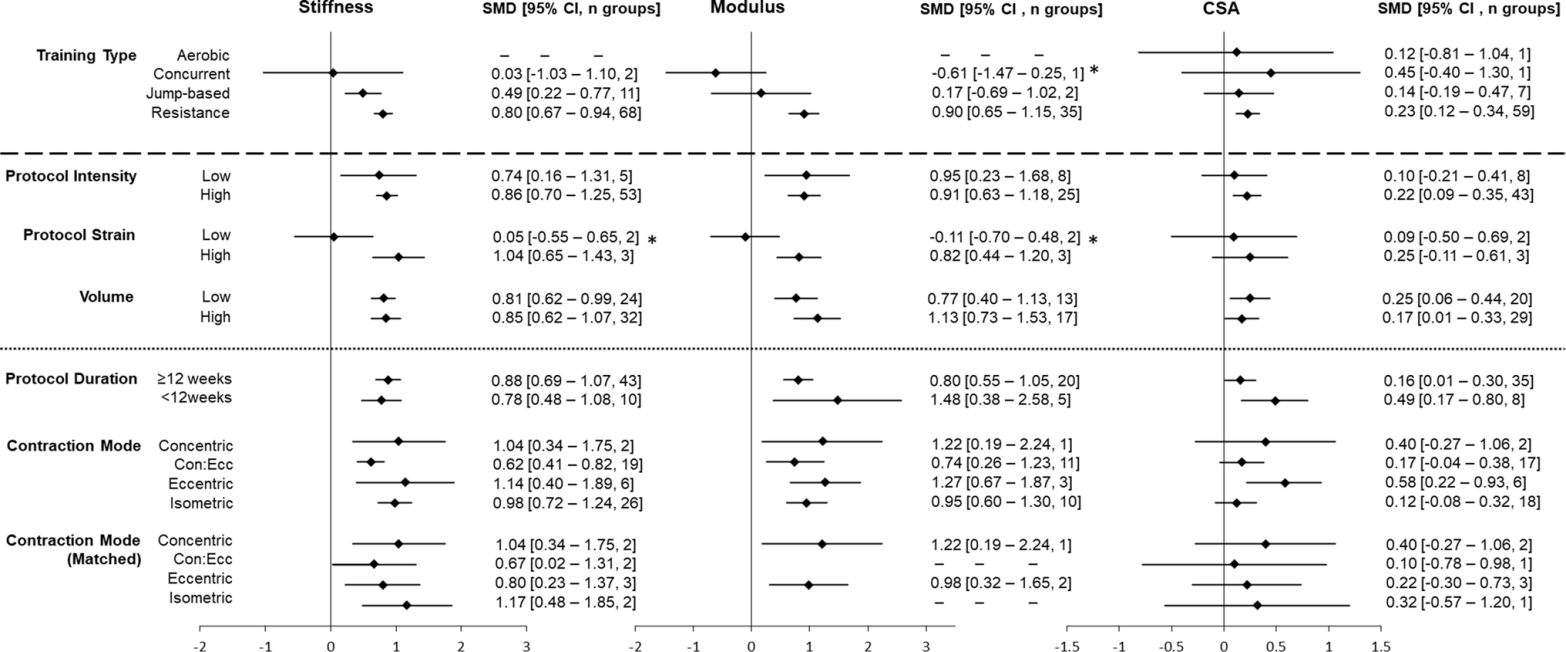
Sub-groups of moderating factors of adaptation in stiffness, modulus and cross-sectional area (CSA), demonstrating standardised mean differences (SMD) and 95% confidence intervals (CI) for each factor. All comparisons beneath the dashed line contain resistance training-only groups. Comparisons beneath the dotted line contain high intensity, resistance training groups. Con:Ecc = concentric:eccentric action; low intensity =  < 70% of maximal voluntary contraction or one repetition maximum; high intensity =  ≥ 70% of maximal voluntary contraction or one repetition maximum, low strain =  ~ 3%; high strain =  ~ 5%; low volume =  ≤ 3100 arbitrary units, high volume =  > 3100 arbitrary units. *p < 0.05 for sub-group analysis

Resistance training (SMD 0.80; 95% CI 0.67–0.94; 68 intervention groups) and jump-based interventions (SMD 0.49; 95% CI 0.22–0.77; 11 intervention groups) induced large and small increases, respectively, in tendon stiffness (Fig. 6, meta-analyses for each tendon property in OSM S7–S9). No change in stiffness was observed after concurrent training (SMD 0.03; 95% CI -1.03–1.10; two intervention groups). Only resistance training resulted in clear increases in modulus and CSA, with large increases in modulus (SMD 0.90; 95% CI 0.65–1.15; 35 intervention groups) and small increases in CSA (SMD 0.23; 95% CI 0.12–0.34; 59 intervention groups). The results of the random-effects model demonstrated significant differences between training interventions for modulus only (*p* = 0.002). Increased modulus following resistance training was significantly greater than concurrent training (*χ*^2^ (1, *n* = 36) = 4.24, *p* = 0.04) but not jump-based training (*χ*^2^ (1, *n* = 37) = 2.00, *p* = 0.17). As resistance training was the only intervention type to demonstrate clear differences in all three outcome measures, sub-group analyses were limited to resistance training interventions only.

While no studies examined aponeurosis CSA, one study [75] reported a significant increase in VL aponeurosis width (relative change = 1.9 ± 3.1%, *p* < 0.05) following a 12-week isotonic knee extension protocol.

### Effect of Loading Parameters on Tendon Adaptation in Resistance Training

All sub-group comparisons for resistance training loading parameters are displayed in Fig. 6. Descriptors (e.g., mean, variance) and individual meta-analyses can be found in OSM S10–S31.

#### Relative Intensity and Strain

High-intensity (≥ 70% 1RM/MVC) resistance training protocols produced large increases in tendon stiffness (SMD 0.86; 95% CI 0.70–1.25; 53 intervention groups) and modulus (SMD 0.91; 95% CI 0.63–1.18; 25 intervention groups), and small increases in CSA (SMD 0.22; 95% CI 0.09–0.35; 43 intervention groups). In comparison, low-intensity (< 70%) resistance training produced moderate increases in stiffness (SMD 0.74; 95% CI 0.16–1.31; five intervention groups), large increases in modulus (SMD 0.95; 95% CI 0.23–1.67; eight intervention groups), and no clear change in CSA (SMD 0.10; 95% CI − 0.21–0.41; eight intervention groups). High-strain (~ 5%) protocols induced large increases in tendon stiffness (SMD 1.04; 95% CI 0.65–1.43; three intervention groups) and modulus (SMD 0.82; 95% CI 0.44–1.20; three intervention groups), with no effect for CSA (SMD 0.25; 95% CI − 0.11–0.61; three intervention studies). Low-strain (~ 3%) protocols did not induce significant increases for any outcome measure. Results of the random effects model demonstrated significant differences between training interventions for stiffness (*p* = 0.007) and modulus (*p* = 0.009). High-strain interventions induced significantly larger increases in tendon stiffness (*χ*^2^ (1, *n* = 7) = 7.45, *p* = 0.006) and modulus (*χ*^2^ (1, *n* = 7) = 6.76, *p* = 0.009) than low-strain groups.

#### Protocol Volume

Both low-volume (SMD 0.81; 95% CI 0.62–0.99; 24 intervention groups) and high-volume (SMD 0.85; 95% CI 0.62–1.07; 32 intervention groups) resistance training interventions induced large increases in tendon stiffness. Low-volume interventions induced moderate increases in modulus (SMD 0.77; 95% CI 0.40–1.13; 13 intervention groups) versus large increases from high-volume protocols (SMD 1.13; 95% CI 0.73–1.53; 17 intervention groups). Low-volume resistance training induced small increases in CSA (SMD 0.25; 95% CI 0.06–0.44; 20 intervention groups) versus trivial CSA increases in high-volume interventions (SMD 0.17; 95% CI 0.01–0.33; 29 intervention groups).

#### Protocol Duration

All high-intensity protocol durations were effective in inducing increases in tendon stiffness, modulus and CSA. No significant differences were found between high-intensity protocols of ≥ 12 and < 12 weeks’ duration for increases in tendon stiffness (≥ 12 weeks: SMD 0.88; 95% CI 0.69–1.07; 43 intervention groups; < 12 weeks: SMD 0.78; 95% CI 0.48–1.08, ten intervention groups), modulus (≥ 12 weeks: SMD 0.80; 95% CI 0.55–1.05, 20 intervention groups; < 12 weeks: SMD 1.48; 95% CI 0.38–2.58; five intervention groups) or CSA (≥ 12 weeks: SMD 0.16; 95% CI 0.01–0.30; 35 intervention groups; < 12 weeks: SMD 0.49; 95% CI 0.17–0.80; eight intervention groups). No included studies manipulated the duration of protocols and so there were insufficient studies available to provide a matched analysis for duration.

#### Contraction Mode

When considering all high-intensity resistance training interventions, all contraction modes produced significant, moderate-to-large increases in tendon stiffness (SMD range 0.62–1.14) and modulus (SMD range 0.74–1.27), but only eccentric contractions produced a significant increase in CSA (SMD 0.58; 95% CI 0.22–0.93; six intervention groups). The analysis of matched interventions revealed similar increases in tendon stiffness (SMD range 0.67–1.17) and modulus (SMD range 0.98–1.22) for all contraction modes, but no significant change in CSA. The fixed effect model demonstrated no significant differences existed between contraction modes for any tendon property in either matched or unmatched analyses.

### Effect of Age on Tendon Adaptation in Resistance Training

There were insufficient studies available to provide a matched analysis for age, and so all high-intensity, adult and elderly intervention groups were synthesised into the following results (full meta-analyses for age are available in OSM S29–S31). Large increases in tendon modulus were found in adult participants (SMD 1.05; 95% CI 0.75–1.34; 21 intervention groups) in comparison to small increases in elderly participants (SMD 0.21; 95% CI − 0.21–0.63; four intervention groups). Large increases in stiffness were found in adults (SMD 0.91; 95% CI 0.74–1.09; 48 intervention groups) versus small increases in elderly participants (SMD 0.40; 95% CI 0.02–0.78; five intervention groups). Only adults experienced clear, albeit small increases in CSA (SMD 0.24; 95% CI 0.10–0.37; 39 intervention groups) versus trivial increases in elderly participants (SMD 0.06; 95% CI -0.37–0.48). There were significant differences between adult and elderly groups in the magnitude of pre-post differences in tendon stiffness (*p* = 0.02) and modulus (*p* = 0.002). Only one, low-intensity study investigated tendon adaptation in children reporting large increases in tendon stiffness (SMD 1.10; 95% CI 0.14–2.05; one intervention group) and modulus (SMD 0.94; 95% CI 0.00–1.87; one intervention group), and trivial increases in CSA (SMD 0.14; 95% CI − 0.74–1.02; one intervention group) [74].

## Discussion

Overall, findings of this systematic review and meta-analysis suggest that high-strain training interventions result in increased tendon stiffness, which is primarily driven by adaptations in material properties (i.e., modulus) rather than morphological properties (i.e., CSA). Sixty-one studies were identified that investigated in vivo adaptations of lower limb tendons to mechanical loading in 763 participants, providing a comprehensive update on two previous systematic reviews examining the effect of mechanical loading on tendon adaptation (Bohm et al. [9], 27 intervention studies; Wiesinger et al. [10], 29 intervention studies of an included 35 studies). The main tendons examined were the AT (33 studies) and the PT (24 studies). The most common training type was resistance training (49 studies), with interventions ranging from 3 to 52 weeks in duration. Meta-analysis of all included studies revealed mechanical loading induced moderate increases in stiffness (SMD 0.74; 81 intervention groups), large increases in modulus (SMD 0.82; 38 intervention groups), and small increases in CSA (SMD 0.22; 68 intervention groups). Resistance training induced consistent increases in stiffness, modulus and CSA; however, no consistent effects were observed when considering jump-based, aerobic and concurrent training modes in isolation. Meta-regression analysis of a subsample of 20 studies that concurrently examined adaptations in CSA, modulus and stiffness revealed that increased tendon stiffness was predominantly explained by increased tendon modulus. Sub-group analyses of three resistance training interventions that directly measured strain further demonstrated that protocols involving high tendon strains (~ 5%) promoted the greatest increase in tendon modulus and stiffness. There was no consistent evidence that relative intensity, protocol duration, contraction mode, or training volume differentially influenced tendon adaptation.

### Adaptations in Tendon Stiffness, Modulus and Cross-Sectional Area (CSA)

The findings from the meta-analysis of all 61 articles in the present review confirm the findings from prior systematic reviews of 29 training studies [10] and 27 training studies [9], that tendon stiffness, modulus and CSA are increased in response to increased mechanical loading. The primary mechanism underlying the training-induced increase in tendon stiffness is believed to be greater strain-induced anabolic gene expression [76] and a corresponding increase in collagen synthesis and turnover [23, 77]. Increased enzymatic cross-linking of collagen [78] may also contribute, although this may not be a factor in older adults [64]. Collectively, these mechanisms can plausibly lead to changes in tendon material and/or morphological properties. However, it should be noted that core tendon tissue may have very limited turnover [79], and adaptation may instead be limited to the peripheral tissue. Transmission of external tendon strain to the mechanosensitive tenocytes occurs via the extracellular matrix and is thought to result in cellular deformation and interstitial fluid flow-induced shear stress of cell cytoskeletons [80, 81]. The present review also provides novel evidence, based on a meta-regression of 20 studies that concurrently assessed stiffness, modulus and CSA, that modulus is the main moderator of increased tendon stiffness. Increased modulus presumably occurs due to an increase in collagen concentration within the extracellular matrix [82], although greater tendon hydration resulting from the proliferation of water binding proteins (i.e., proteoglycans) might also contribute [83]. These data support the hypothesis that material properties are the main driver of increased tendon stiffness [9].

### Influence of Training Type on Tendon Adaptation

Four broad types of training interventions were identified in the included studies: aerobic training, resistance training, concurrent aerobic and resistance training, and jump-based activity. Only resistance training (SMD 0.80; 68 intervention groups) and jump-based activity induced clear changes in tendon stiffness (SMD 0.49; 11 intervention groups), with resistance training being the only intervention type to increase modulus (SMD 0.90; 35 intervention groups) and CSA (SMD 0.23; 59 intervention groups). Due to the viscoelastic properties of the extracellular matrix, tendons deform more and therefore absorb more energy, at low compared to high strain rates [84]. As a consequence, longer duration contractions at any given load may augment the transmission of external tendon strain to the mechanosensitive tendon cells, which would be expected to promote greater anabolic responses [21, 22, 77]. The relatively brief ground contact times and greater rates of tendon loading associated with plyometric activity may not provide an optimal stimulus for adaptation. In support of this, studies that directly compared the effects of sustained loading (i.e., concentric:eccentric or isometric interventions) to jump-based activity, only observed increased tendon stiffness in the former [21, 85, 86]. Interestingly, when resistance training was performed concurrently with aerobic exercise (i.e., running or cycling), no significant change in any tendon property was observed. It is well established that concurrent training—as is commonly performed by athletes—attenuates gains in skeletal muscle size and strength compared to resistance training alone [87]. The mechanisms underpinning this “interference” effect are poorly understood, although it is possible that the molecular signalling responses to aerobic exercise inhibit protein synthesis and stimulate protein breakdown [87]. Future work is needed to determine the mechanism(s) by which concurrent training blunts loading-induced adaptations in tendon mechanical, material and morphological properties.

### Effect of Loading Parameters on Tendon Adaptation in Resistance Training

Resistance training was the most commonly employed intervention in the available literature (49 studies), and the only training type to elicit significant increases in stiffness, modulus and CSA. Consequently, sub-group analyses to determine the effect of key training parameters and the effect of participant age were limited to resistance training groups only. Protocols were predominantly undertaken by adults (18–60 years), and typically consisted of high-intensity (≥ 70% 1RM or MVC) concentric:eccentric or isometric contractions, performed over a period of ≥ 12 weeks. There is evidence to suggest that resistance training performed using all contraction modes, of all durations, of high or low intensity, and of high or low volume elicit clear but similar increases in tendon stiffness. However, when considering only high-intensity studies’ results, the magnitude of pre-post intervention difference in stiffness and modulus was greater in adults than in elderly participants. Importantly, there is strong evidence that high strain protocols demonstrate significantly greater increases in tendon stiffness and modulus than low strain interventions.

Evidence from two studies that plantar flexor training performed at ~ 5% AT strain for 14 weeks demonstrated increased tendon-aponeurosis stiffness and modulus, and regional hypertrophy, whereas training performed at ~ 3% strain did not [19, 20]. Larger whole tendon strains could theoretically result in larger localised tendon deformations, which would be expected to result in more tenocytes experiencing more strain. The overall findings of the present review support previous in vitro observations [25–27], and suggest that there may be a load/strain threshold that must be exceeded for positive tendon adaption to occur in vivo.

No clear relationships were found between tendon adaptations and protocol intensity, protocol volume, the contraction mode(s) used, or protocol duration. In disagreement with an earlier systematic review on tendon adaptation following mechanical loading [9], the present review did not demonstrate significant differences between high- (≥ 70% 1RM/MVC) and low-intensity (< 70% 1RM/MVC) protocols (Fig. 6). These data suggest the possibility that the relative intensity of an exercise may not reflect the actual stress and/or strain experienced by the tendon. However, between-study differences in intervention characteristics including exercise selection, range of motion, velocity, contraction duration, and the absence of reported tendon strain may confound these findings.

Previous mechanical loading reviews [9, 10] reported that protocol duration was not a key moderator of lower limb tendon adaptation following resistance training and the present review demonstrated that all outcome measures increased regardless of protocol duration. Three included studies [72, 88, 89] documented tendon property measurements across multiple time-points, but only one, single-group study concurrently measured all three main outcomes (stiffness, modulus, CSA) [72]. These studies suggest that tendon stiffness changes are notable between 4 [72] and 8 weeks [88, 89] of training, with corresponding early changes in material properties [72]. However, there is conflicting evidence on the time-course of morphological adaptation, with one study reporting CSA changes after 8 weeks of training [72], and others observing no increases after 12 weeks of training [88, 89]. Cross-sectional studies investigating the size of lower limb tendons in habitual runners, fencers and badminton players [90, 91] suggest that tendon hypertrophy may occur to a greater extent following long-term (i.e., > 12 months) mechanical loading, and this should be a focus of future work.

All contraction modes induced significant increases in tendon stiffness and modulus; however, only high-intensity eccentric interventions evoked a clear increase in tendon CSA when considering all intervention groups in this category. However, when studies were matched for analysis, i.e., including only those studies that manipulated contraction mode, this effect was lost. Eccentric contractions can allow for the development of higher forces than concentric or isometric contractions [92, 93], which might be expected to stimulate greater localised tendon strains. In the present systematic review, the majority of eccentric-only group protocols featured relative intensities of ≥ 80% 1RM/MVC, including use of protocols with supramaximal concentric:eccentric loads or based on eccentric-only repetition maximum loads. In comparison, isometric protocol intensity ranged from 50 to 90% 1RM/MVC and concentric:eccentric loads ranged from 50 to 85%. Heinemeier et al. [94] demonstrated that rat tendons exposed to mechanical loading via concentric, isometric and eccentric contractions all displayed similar insulin-like growth factor (IGF-1) and collagen expression, despite impulse being greater in the eccentric actions. In summary, contraction mode does not appear to be a key programming variable; however certain contraction modes may be beneficial for inducing higher strains.

### Effect of Age on Tendon Adaptation in Resistance Training

Finally, adult participants in the included studies demonstrated consistent increases in tendon stiffness, modulus and CSA (Fig. 6); however, children (< 18 years) and elderly participants (≥ 60 years) still experienced increased tendon stiffness and modulus following resistance training. Elderly participants experienced small increases in both tendon stiffness and modulus, which suggests ageing has a dampening effect on the magnitude of adaptation. Consequently, the present review suggests that resistance training interventions may be beneficial for tendons across all ages, but that practitioners should be aware of lower levels of effectiveness in elderly populations.

### Limitations and Future Directions

The following limitations of this review are acknowledged. While efforts were made to provide sub-group analyses based on similar interventions, significant heterogeneity existed between protocols. For example, isometric interventions were grouped together but contraction duration and thus time under tension were not matched. Further, while groups from the same studies could be matched for the meta-regression, it was not possible to match methods of measurement across all three tendon properties for the sub-group analyses, resulting in a small number of intervention groups for some analyses. This was particularly evident when assessing the influence of training protocol strain, as only three included studies provided this information. To minimise heterogeneity in the group comparisons of training duration, contraction mode, and age, we only synthesised the effects of high-intensity (≥ 70% 1RM/MVC) resistance training interventions. Where possible, we also performed additional analyses on matched interventions that had only manipulated a specific training variable (meaning intervention groups were matched to at least one other group and thus directly comparable).

Adaptations in free tendons and aponeuroses were considered together, despite having different structure and mechanical function within the musculotendinous unit. Wiesinger et al. [10] reported that the patellar and Achilles free tendons responded similarly to a period of mechanical loading; however, it is unclear if aponeuroses may differ given their interaction with muscular tissue.

Conclusions were based on a large number of relatively small studies, with all but one study [95] including ≤ 15 participants. Additionally, the majority of participants in the included studies were classified as untrained or recreationally active with no consistent histories of resistance training. Only three studies recruited well-trained athletes – including highly trained distance runners [60], female endurance athletes [59], and strength-trained males [58] – all of which displayed no change in PT stiffness even following high-intensity training. As such, caution should be applied when directly applying the findings of this review to highly trained populations.

In the present review, only Eriksen et al. [55] studied tendon adaptation for a period longer than 14 weeks. Consequently, it is not possible to provide clear conclusions on the time-course of adaptation beyond this timeframe. Studies investigating longer term (i.e., > 14 weeks) interventions may be beneficial in furthering our understanding of the effects of long-term loading on tendon stiffness and the relative contributions of changes in material versus morphological properties.

The use of Egger’s test and funnel plots demonstrated some evidence of small study bias; however, adjusted SMDs (OSM S2–S4) showed no substantial differences to the original SMDs and so the conclusions of the review and meta-analysis remain unchanged. The calculation of SMDs was used in the present meta-analyses rather than standardised mean change (SMC) due to not all studies reporting sufficient information to calculate SMC, some studies not including a control group, and heterogeneity between existing control groups, for example, active versus passive controls. Additionally, heterogeneity existed between studies in the methods of measurement employed for each key outcome and the location of measurement; however, the use of SMDs allows appropriate comparison where measurement methods are not identical.

No adjuncts to training, such as blood-flow restriction or electrical muscle stimulation, were included in the present systematic review. There is emerging evidence from two studies [71, 96], that suggest that blood-flow restriction training with very light loads might also be effective in promoting tendon adaptation.

The overall quality of included studies was moderate to high and no studies were excluded based on the quality assessment. However, several criteria were under-reported in the included studies. Allocation concealment (reported in 7% of studies) and assessor blinding (18% of studies) were poorly reported, and this could introduce investigator bias into some studies. Approximately half of included papers did not clearly report that all participants received the allocated condition and that data for > 85% of participants were included in the results. Previous studies have demonstrated that underreporting of these criteria may overestimate treatment effects [97–99]. Randomisation of groups, which is important for interpreting intervention effects, was not reported in 21% of included studies, and, further, the inclusion of non-randomised, single-group designs may hinder interpretation of results. Additionally, the PEDro criteria assess blinding of participants and therapists delivering interventions, which is not typically possible in exercise-based protocols, such as those included in the current review. Further, some of the criteria relate to multiple groups and are not applicable to single-group study designs. To aid interpretation, we calculated a relative score as a percentage of the total score divided by the total possible score for each design as per a previous review [39]; however, studies with different objectives were included in the current systematic review and designs may not be directly comparable. Future work may consider other risk of bias tools which provide the possibility of evaluating individual domains in a more differentiated way.

Consistent with in vitro studies [25–27], evidence from two studies in our review [19, 20] indicated that adaptation was more pronounced when the tendon was exposed to ~ 5% strains compared to ~ 3% strains. These two included studies were also notable because the dose of mechanical strain applied to the tendon was measured and controlled. Although it is likely that the external loads applied to the body during tendon training (alongside the muscle–tendon length and contraction velocities) are related to the load applied directly to the tendon, we recommend that the mechanical stimulus applied to the tendon be measured and controlled in future studies so that tissue-level dose–response relationships can be better understood. This is important because tendon stress and/or strain could differ substantially between individuals performing the same exercise, and because it could ultimately allow more targeted and personalised exercise prescription. The next generation of technologies that help match the applied tissue loading with the loading conditions that elicit maximal tendon adaptation are already being developed [100–103]. The results of this systematic review and meta-analysis were based on interventions related to the AT, the PT or QT, and VL aponeurosis. Consequently, it may be useful to determine whether other lower limb tendons demonstrate similar types and magnitudes of adaptation. Further, it would be beneficial to determine the extent to which the tendinous adaptations identified in this review influence athletic performance and injury risk. Finally, caution is warranted in applying the findings of the present review to pathological tendons since the tissue state in a tendinopathic tendon is altered, and may undergo different biological responses to the same mechanical stimulus. Exploration of the mechanical, material and morphological adaptations of pathological tendons to mechanical loading should be a focus of future work.

### Practical Applications

The results of this systematic review and meta-analysis indicate that resistance training is the most effective training strategy for promoting adaptation in tendon mechanical, material and morphological properties for all age groups, although the magnitude of change may vary with age. Jump-based protocols also induced increases in stiffness but to a lesser extent than resistance training. In contrast, aerobic training performed in isolation appears to have no effect on tendon properties, and may in fact blunt positive adaptations if performed concurrently with resistance training. High strain (~ 5%) resistance training interventions induced large increases in tendon stiffness and so preference should be given to the application of strain to tendons if this can be estimated in clinical practice. However, should this not be possible, prescription of interventions using a very high relative intensity (e.g., ≥ 90% 1RM/MVC), as per the high-strain protocols, may indirectly elicit high strain on tendons and induce positive adaptation. Further, while contraction mode does not appear to influence tendon adaptation, some contraction modes may act as better vehicles for delivering higher intensities and strains.

## Conclusion

This review found consistent evidence that mechanical loading promotes moderate increases in tendon stiffness and modulus, and small increases in CSA. Meta-regression analysis further revealed that the training-induced increases in tendon stiffness are primarily explained by material and not morphological adaptations. Resistance training was found to be the loading regime that stimulated the greatest change in tendon properties. In contrast, there was no consistent evidence that jump-based, aerobic and concurrent training modes lead to positive tendon adaptation. We also found strong and consistent evidence that high-strain resistance training interventions elicited significantly greater changes in tendon stiffness and modulus than low-strain interventions, and that differences exist between the magnitude of adaptation between adult and elderly participants. Evidence to date suggests that tendon adaptation is not significantly influenced by protocol duration, relative protocol intensity, contraction mode, or training volume. Therefore, high-strain resistance training protocols are likely to be most beneficial where tendon adaptation is a desired outcome. These findings may have important implications for strategies targeted at improving performance and reducing injury risk across a range of athletic populations.

## Supplementary Information

Below is the link to the electronic supplementary material.

Supplementary file1 (PDF 7231 kb)

## Declarations

### Availability of data and material

Available on request.
