# Supplementary material for: Mechanical, Material and Morphological Adaptations of Healthy Lower Limb Tendons to Mechanical Loading: A Systematic Review and Meta-Analysis
Source: Sports Med. 2022 Jun 3;52(10):2405–29. doi: 10.1007/s40279-022-01695-y (PMC9474511; doi:10.1007/s40279-022-01695-y)

Supplementary information for:

TITLE: Mechanical, morphological and material adaptations of healthy lower limb tendons to mechanical loading: A systematic review and meta-analysis

JOURNAL: Sports Medicine

AUTHORS

Lazarczuk, Stephanie L.[1, 2] (ORCID: 0000-0001-8467-8799)

Maniar, Nirav[3, 4] (ORCID: 0000-0002-6180-6003)

Opar, David A.[3, 4] (ORCID: 0000-0002-8354-6353)

Duhig, Steven J.[1, 2] (ORCID: 0000-0002-4014-7731)

Shield, Anthony[5] (ORCID: 0000-0002-0393-2466)

Barrett, Rod S.[1, 2] (ORCID: 0000-0002-1784-1629)

Bourne, Matthew N.[1, 2] (ORCID: 0000-0002-3374-4669)

AFFILIATIONS

1. School of Health Sciences and Social Work, Griffith University, Gold Coast, Australia
2. Griffith Centre of Biomedical and Rehabilitation Engineering (GCORE), Menzies Health Institute Queensland, Griffith University, Gold Coast, Australia
3. School of Behavioural and Health Sciences, Australian Catholic University, Melbourne, Australia
4. Sports Performance, Recovery, Injury and New Technologies (SPRINT) Research Centre, Australian Catholic University, Melbourne, Australia
5. School of Exercise and Nutrition Sciences and Institute of Health and Biomedical Innovation, Queensland University of Technology, Brisbane, Australia

CORRESPONDING AUTHOR:

Stephanie L. Lazarczuk – stephanie.lazarczuk@griffithuni.edu.au

S1. Search strategy and limits

1. Location	2. Tendon Tissue	3. Trg Intervention	4. Tendon Properties	5. NOT
OR<br><br>Lower limb<br>Hamstring*<br>Vastus lateralis<br>Quadricep*<br>Achilles<br>Patella*<br>Adductor*<br>Gastrocnemius<br>Soleus	OR<br><br>Tend*<br>Aponeuros*	OR<br><br>Strength*<br>Resistance<br>Run*<br>Sprint*<br>Power<br>Endurance<br>jump*<br>plyometric<br>ballistic<br>bound*<br>land*<br>stretch short*<br>SSC<br><br>AND<br><br>(Load* OR<br>Exercis* OR<br>Training OR<br>Intervention)	OR<br><br>Adapt*<br>Modulus<br>Stiffness<br>Cross-sectional area<br>CSA<br>Morphology<br>Geometry<br>Material prop*<br>Mechanical prop*<br>Complian*<br>Stress<br>Strain<br>Deformation<br>Elongation<br>Plasticity	OR<br><br>ACL<br>Anterior cruciate ligament<br>Tendinopath*<br>Tendinitis<br>Tendinosis<br>Rupture<br>Reconstruction<br>Graft<br>Injur*

Strategy: The terms in each column were searched using the Boolean operator above them. These searches were combined using the following strategy for titles, abstracts: (1 AND 2 AND 3 AND 4) NOT 5

The following database keywords were also searched in addition to the title and abstract search above: PubMed = MeSH Terms; Scopus = Keywords; CINAHL = Subject Terms ; SportDISCUS = Subject Terms; EMBASE = Author key terms

Limits applied: Human(s), English

A manual check of reference lists of included studies and similar reviews was also conducted.

S2. Funnel plot for all studies reporting stiffness, demonstrating the standardised mean differences versus standard error, with result for Egger's test and adjusted SMDs based on the methods in Vevea & Woods (2005).

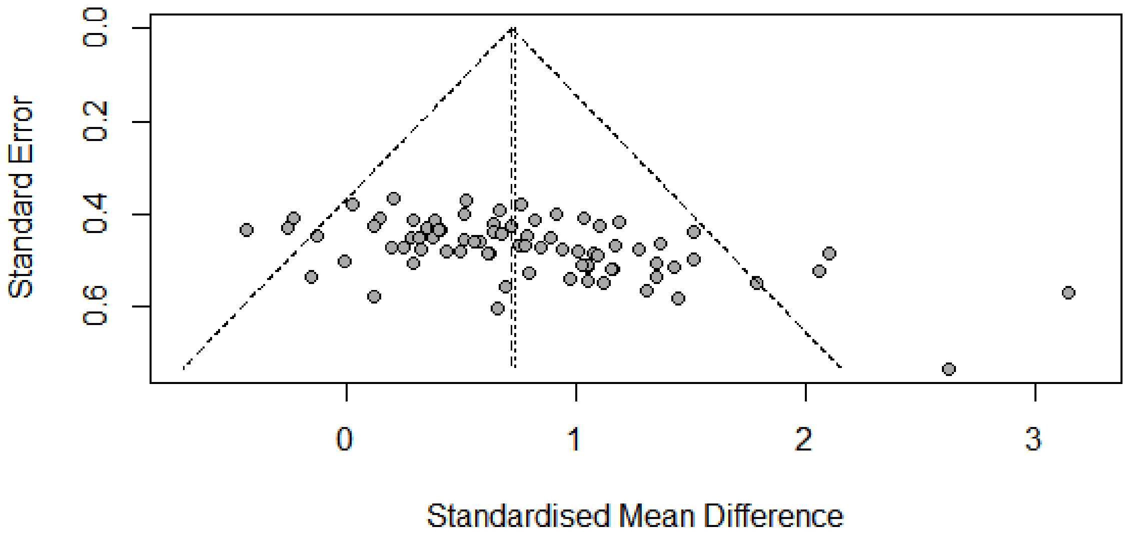

Egger's: intercept = 4.90, t = 4.89, p < 0.001

Original SMD: 0.74, 95%CI 0.62 – 0.86

Adjusted SMD – moderate bias: 0.63, 95%CI 0.50 – 0.76

Reference: Vevea JL, Woods CM. Publication bias in research synthesis: Sensitivity analysis using a priori weight functions. Psychol Methods. 2005;10(4):428–43.

S3. Funnel plot for all studies reporting modulus, demonstrating the standardised mean differences versus standard error, with result for Egger's test and adjusted SMDs based on the methods in Vevea & Woods (2005).

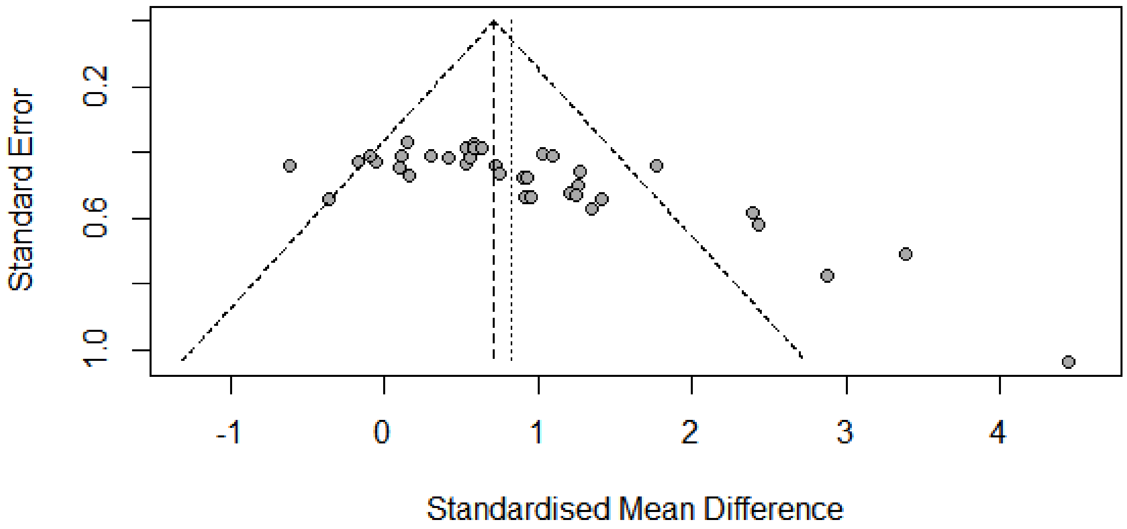

Eggers: intercept = 6.39, t = 5.99, $p < 0.001$

Original SMD: 0.82, 95%CI 0.58 – 1.07

Adjusted SMD – moderate bias: 0.65, 95%CI 0.39 – 0.92

Reference: Vevea JL, Woods CM. Publication bias in research synthesis: Sensitivity analysis using a priori weight functions. Psychol Methods. 2005;10(4):428–43.

S4. Funnel plot for all studies reporting cross-sectional area, demonstrating the standardised mean differences versus standard error, with result for Egger's test and adjusted SMDs based on the methods in Vevea & Woods (2005).

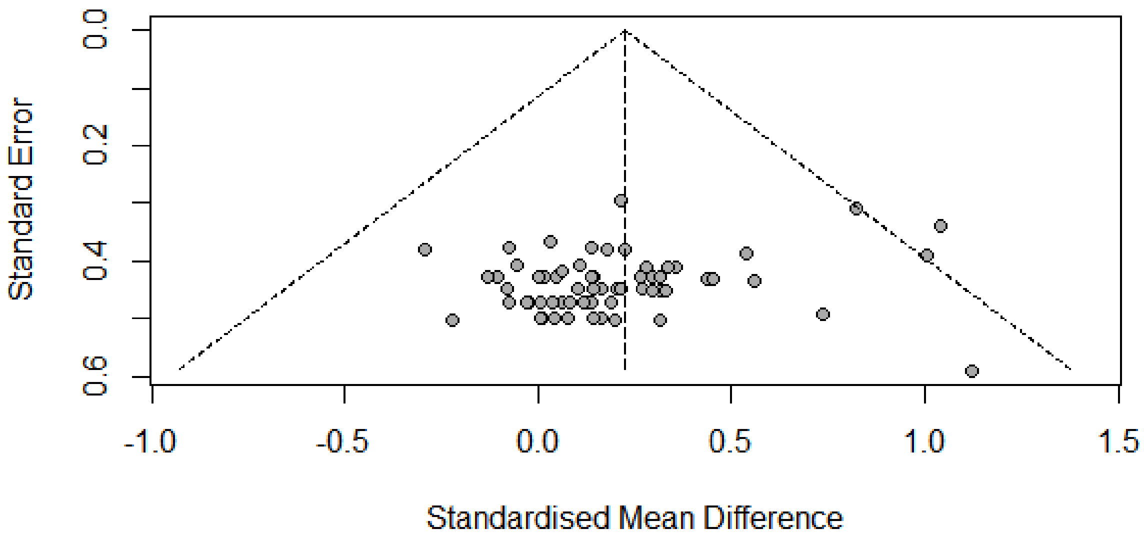

Eggers: intercept = -1.56, t = -2.27, p = 0.026

Original SMD: 0.22, 95%CI 0.12 – 0.33

Adjusted SMD – moderate: 0.14, 95%CI 0.04 – 0.24

Reference: Vevea JL, Woods CM. Publication bias in research synthesis: Sensitivity analysis using a priori weight functions. Psychol Methods. 2005;10(4):428–43.

S5. Study characteristics (extended).

Source	Participants	Intervention		Tendon	
Author	Group	Duration (weeks), Frequency per week	Exercise Parameters/Activity Descriptor	Outcome measures - method	Tissue
Albracht et al., 2013 [5]	Exercise	14, 4	Unilat iso PF @ 5° DF (knee extended)<br>5 x 4 @ 90% MVC<br>3s contract, 3s rest	Stiffness: ramped iso PF MVC on isokinetic dynamometer with 2D US to assess elongation of distal GaM fascicles and apon<br>Modulus: not assessed<br>CSA: not assessed	AT
	Control (rec active)		Continued own endurance training (running, ≥3 x p/wk)		
Arampatzis et al., 2007 [19]	Low strain limb<br><br>High strain limb	14, 4	Unilat iso PF @ 85° DF (knee extended)<br>Low strain: 5 x 7 @ 55% MVC = 2.85 ± 0.99% strain<br>High strain: 5 x 4 @ 90% MVC = 4.55 ± 1.38% strain<br>3s contract, 3s rest	Stiffness: ramped iso PF MVC on isokinetic dynamometer with 2D US to assess elongation of distal GaM fascicles and apon<br>Modulus: calculated from linear regression of the tendon stress-tendon/aponeurosis strain relationship between 50-100% of maximum tendon stress<br>CSA: T1 MRI; 10% intervals along length	AT
	Control *		No exercise intervention		
Arampatzis et al., 2010 [20]	Low strain limb<br><br>High strain limb	14, 4	Unilat iso PF @ 85° DF (knee extended)<br>Low strain: 5 x 20 @ 55% MVC @ 2.97 ± 0.47% strain<br>High strain: 5 x 12 @ 90% MVC @ 4.72 ± 1.08% strain<br>1s contract, 1s rest	Stiffness: ramped iso PF MVC on isokinetic dynamometer with 2D US to assess elongation of distal GaM fascicles and apon<br>Modulus: calculated from linear regression of the tendon stress-tendon/aponeurosis strain relationship between 50-100% of maximum tendon stress<br>CSA: T1 MRI; 10% intervals along length	AT
Baptista et al., 2016 [95]	Concentric limb<br><br>Eccentric limb	12, 2	Unilat con or ecc knee extension<br>Con: 2 x 10 @ ~80% 5RM<br>Ecc: 2 x 10 @ ~80% 5RM<br>0.5 s load acceptance + 3 s con or ecc	Stiffness: not assessed<br>Modulus: not assessed<br>CSA: 2D US; 50% of distance between patella apex and tibial insertion	PT
Bohm et al., 2014 [21]	High strain rate limb & Reference protocol limb<br><br>Long strain duration limb & Reference protocol limb	14, 4	Unilat hops<br>High strain rate: 5 x 72 @ 90% MVC = 6.63 ± 1.24% maximum strain<br><br>Unilat iso PF<br>Long strain duration: 5 x 12s @ 90% MVC = 6.94 ± 1.54% maximum strain<br><br>Reference protocol (completed on non-intervention limb):<br>Unilat iso PF @ 5° DF (knee extended): 5 x 4 (3s contract, 3s rest) @ 90% MVC	Stiffness: iso PF MVC on isokinetic dynamometer with 2D US to assess elongation at GaM MTJ, calculated from linear regression of the tendon force-tendon elongation ratio between 50-100% maximum tendon force<br>Modulus: calculated from linear regression of the tendon stress-tendon strain relationship between 50-100% maximum stress<br>CSA: MRI, 10% intervals along length	AT
	Control *		No exercise intervention		

Bohm et al., 2021 [17]	Intervention	14, 3-4	Unilat iso PF<br>5 x 4 @ 90% MVC<br>3 s contract, 3 s rest	Stiffness: ramped iso PF MVC on isokinetic dynamometer with 2D US to assess elongation at GaM MTJ, calculated between 50-100% max tend force and strain<br>Modulus: not assessed<br>CSA: not assessed	AT
	Control (rec active)		Continued own endurance training (running, ≥2 x p/wk)		
Carroll et al., 2011 [62]	Placebo (control = extracted group)	12, 3	Bilat con:ecc knee extension<br>3 x 10 @ 74 ± 1% 1RM (mean)<br>120s inter-set rest	Stiffness: ramped iso MVC in seated, force recorded via strain-gauge, with 2D US used to assess displacement of patella and tibial insertions, calculated from final 10% of force-elongation curve<br>Modulus: calculated from final 10% of stress-strain curve<br>CSA: MRI; proximal, middle and distal regions	PT
	Acetaminophen		Resistance training + 4000 mg acetaminophen (daily)		
	Ibuprofen		Resistance training + 1200 mg ibuprofen (daily)		
Centner et al., 2019 [71]	Heavy load (extracted group)	14, 3	Standing and seated con:ecc PF<br>3 x 6-12 each exercise @ 70-85% 1RM<br>60 s inter-set rest<br>180 s inter-exercise rest	Stiffness: ramped iso PF on isokinetic dynamometer with 2D US to assess elongation at GaM MTJ, calculated as the slope of the force-elongation curve between 50-80% MVC<br>Modulus: slope of the stress-strain curve between 50-80% MVC<br>CSA: 2D US at 25% length of AT length (measured calcaneal tuberosity to most distal aspect Gastrocnemius)	AT
	Low load + BFR		Exercises above @ 20% ⟶ 35% 1RM<br>First set: 1 x 30<br>Subsequent sets: 3 x 15<br>@ 50% limb occlusive pressure		
	Control (rec active)		No exercise intervention		
Dalgaard et al., 2019 [104]	Non-contraceptive (extracted group)	10, 3	Con:ecc Knee extension and incline leg press<br>Wk 1: 3x12 @ 15RM ⟶ Wk 6-10: 4x10 @10RM.	Stiffness: not assessed<br>Modulus: not assessed<br>CSA: T1 MRI; proximal, middle and distal regions	PT
	Contraceptive		Exercises as above + oral contraceptives		
Duclay et al., 2009 [105]	Eccentric training	7, 3	Unilat ecc calf raise<br>6 x 6 @ 120% concentric 1RM<br>180s inter-set rest<br>1 x session per week seated (calf machine) @ 90 knee° flexion, other sessions supine (sled)<br>18 sessions total	Stiffness: ramped iso PF MVC on isokinetic dynamometer in prone lying, with 2D US to assess elongation at GaM at distal myotendinous junction; assessed at 10% intervals of MVC torque<br>Modulus: not assessed<br>CSA: not assessed	AT
	Control (rec active)		No exercise intevention		

Eriksen et al., 2018 [63]	Old heavy resistance<br><br>Very old heavy resistance	12, 3	Knee con:ecc extension, leg press & leg curls<br>Wk 1: 3 x 12 @ 12RM (~70% 1RM) → Wk 10: 5 x 6 @ 6RM (~90% 1RM) → Wk 11: 3 x 6 @ 6RM → Wk 12: 2 x 6 @6RM	Stiffness: iso knee extension MVC in seated, force recorded via strain-gauge, with 2D US to assess elongation of PT between patella and tibial tendon insertions, calculated from the final 10% of force-elongation curve.<br>Modulus: calculated from final 10% of stress-strain curve (inferred from citations)<br>CSA: T1 MRI; proximal, middle and distal regions	PT
	Control (sedentary)		No exercise intervention		
Eriksen et al., 2019 [64]	Heavy resistance (extracted group)	52, 3	Knee con:ecc extension & leg press<br>6-8 wk of 3 x 15 @ ~50-60 %1RM.<br>8 wk blocks (1 wk inter-block break).<br>3 x 12 @ 70% → 3 x 6 @ 85% 1RM	Stiffness: iso knee extension in seated, force recorded via dynamometer, with 2D US to assess elongation of PT between patella and tibial tendon insertions, calculated from the final 10% of the force-elongation curve<br>Modulus: calculated from the final 10% of the stress-strain curve<br>CSA: T1 MRI; proximal, middle and distal regions	PT
	Moderate resistance		Unsupervised, home-based circuit + elastic band activity		
	Control (habitual activity)		No exercise intervention		
Farup et al., 2014 [106]	Placebo Concentric limb<br><br>Placebo Eccentric limb	12, 3	Unilat con:ecc knee extension<br>6 x 10-15RM → 8 x 6-10RM<br>Eccentric = 120% concentric load<br>Concentric = 2s, Eccentric = 2s<br>120s inter-set rest<br>33 sessions total	Stiffness: not assessed<br>Modulus: not assessed<br>CSA: T1 MRI; proximal, middle and distal regions	PT
	Whey hydrolysate (not extracted)		Exercise above + high-leucine whey protein hydrolysate + carbohydrate supplementation		
Fletcher et al., 2010 [60]	Isometric	8, 3	Unilat iso PF<br>4 x 20s @ 80% MVC<br><br>R: 70-170 km/wk	Stiffness: ramped iso PF MVC on isokinetic dynamometer in prone lying, with 2D US to assess elongation at GaM deep apon; assessed between 25-45%, 30-70% and 50-100% of MVC force<br>Modulus: not assessed<br>CSA: not assessed	AT
	Control (active)		Continued own endurance training (running, ≥6 x p/wk)		
Fouré et al., 2009 [107]	Training/Jump	8, 2	SJ, CMJ, DJ, hurdles (DL to SL combos)<br>150-280 jumps per session<br>Progressive increase in number of jumps and heights over first 5 wk (detail n/s)	Stiffness: iso PF MVC on isokinetic dynamometer in prone lying, with 2D US to assess elongation at Ga MTJ, calculated as the slope of force-elongation values<br>Modulus: not assessed<br>CSA: not assessed	AT
	Control (rec active)		No exercise intervention – habitual exercise		

Fouré et al., 2010 [49]	Training/Jump	14, n/s	SJ, CMJ, DJ (@ 40cm, 60cm, or 80cm), hurdle hops/jumps.<br>200-600 jumps/session ≈ 6800 jumps total in programme<br>34 sessions total	Stiffness: iso PF MVC on isokinetic dynamometer in prone lying, with 2D US to assess elongation at GaM MTJ, calculated as the slope of the force-elongation curve between 50-90% maximum force<br>Modulus: not assessed<br>CSA: 2D US, level with medial malleolus	AT
	Control (rec active)		No exercise intervention – habitual exercise		
Fouré et al., 2011 [50]	Training/Jump	14, n/s	SJ, CMJ, DJ (@ 35cm, 50cm, 65cm), hurdle hops/jumps (40cm hurdle)<br>200-600 jumps/session<br>Progressive increase in number of exercises, jumps, and/or height (detail n/s)<br>34 sessions total	Stiffness: not assessed<br>Modulus: not assessed<br>CSA: 2D US, level with medial malleolus	AT
	Control (rec active)		No exercise intervention – habitual exercise		
Fouré et al., 2012 [51]	Jump	14, n/s	SJ, CMJ, DJ (@ 40cm, 60cm, or 80cm), hurdle hops/jumps.<br>200-600 jumps/session ≈ 6800 jumps total in programme<br>34 sessions total	Stiffness: not assessed<br>Modulus: not assessed<br>CSA: 2D US, level with medial malleolus	AT
	Control (rec active)		No exercise intervention – habitual exercise		
Fouré et al., 2013 [108]	Eccentric	14, n/s	Unilat ecc heel drops + jump/landings from 35/50/65cm box (landing: unilateral or bilateral).<br>Progressive increase in number of PF actions or height of jump (increments n/s)<br>200-600 ecc actions/session ≈ 6800 contractions total in programme<br>34 sessions total	Stiffness: iso PF MVC on isokinetic dynamometer in prone lying, with 2D US to assess elongation at GaM MTJ, calculated as the slope of the force-elongation curve between 50-90% maximum force<br>Modulus: not assessed<br>CSA: 2D US, level with medial malleolus	AT
	Control (rec active)		No exercise intervention – habitual exercise		
Geremia et al., 2018 [72]	Eccentric	12, 2	Unilat ecc calf raises<br>3-5 x 10 @ 100% MVC<br>60 s inter-set rest<br>23 sessions total	Stiffness: ramped iso PF MVC on isokinetic dynamometer, with 2D US to assess elongation at GaM MTJ, calculated as the slope of the force-elongation curve from 50-100% MVC<br>Modulus: calculated as the slope of the stress-strain curve from 50-100% MVC<br>CSA: 2D US at 2, 4 and 6 cm from calcaneal insertion	AT
Hirayama et al., 2017 [109]	Training	12, 3	Unilat sled depth jumps<br>10 x 10 @ 100%<br>30 s inter-rep rest	Stiffness: iso PF MVC on myometer in prone lying, with 2D US to assess displacement of GaM fascicle intersection at deep apon, calculated as the slop of the force-elongation curve from 50-100% peak torque<br>Modulus: not assessed<br>CSA: not assessed	AT
	Control (rec active)		No exercise intervention		

Houghton et al., 2013 [73]	Plyometric	8, 2	Various unilat/bilat horizontal and lateral jump exercises.<br>Varying intensity.<br>15 sessions total	Stiffness: ramped iso PF MVC on isokinetic dynamometer in prone lying, with 2D US to assess elongation at GaM MTJ, calculated as the gradient of linear regressions against the force-elongation curve between 0-40% and 50-90% MVC<br>Modulus: calculated as the gradient of linear regression against the stress-strain curve between 50-90% peak stress<br>CSA: 2D US, 2cm superior to line between medial and lateral malleoli	AT
	Control (rec active)		No exercise intervention – habitual exercise		
Kay et al., 2016 [110]	Training	6, 2	Unilat PF @ 20° PF, sustained contraction with passive DF to 10° (i.e., 30° ROM) = induced ecc<br>5 x 12 @ 100%<br>1 s inter-rep rest<br>60 s inter-set rest	Stiffness: ramped iso PF MVC on isokinetic dynamometer, with 2D US to assess elongation at GaM MTJ, calculated as change in PF moment from 50-90% MVC divided by elongation<br>Modulus: not assessed<br>CSA: not assessed	AT
Kongsgaard et al., 2007 [65]	Heavy Resistance limb	12, 3	Unilat con:ecc knee extension<br>Heavy: 10 x 8 @ 70% 1RM<br>180 s inter-set rest	Stiffness: ramped iso knee extension MVC using strain gauge, with 2D US to assess elongation of PT between patella and tibial insertions, calculated in final 10% of force-elongation curve<br>Modulus: calculated in the final 10% of stress-strain curve<br>CSA: T1 MRI; proximal, middle and distal regions	PT
	Light Resistance limb		Light: 10 x 36 @ equivalent load (not reported)<br>30 s inter-set rest		
Kubo et al., 2001 [51]	Short duration limb	12, 4	Unilat iso knee extension @ 80° knee flexion<br>Short duration: 3 x 50 rapid contraction @ 70% MVC<br>2 s inter-rep rest<br>60 s inter-set rest	Stiffness: ramped iso knee extension MVC on isokinetic dynamometer, with 2D US to assess elongation of VL apon at 50% length of thigh visualising fascicle insertion into apon, calculated as the slope of linear regression over 50-100% MVC<br>Modulus: not assessed<br>CSA: T1 MRI, prone lying, immediate superior to patella and 10mm from patella	QT<br>VL apon
	Long duration limb		Long duration: 4 x 20s @ 70% MVC<br>60 s inter-rep rest.		
Kubo et al., 2001 [52]	Isometric	12, 4	Unilat iso knee extension @ 80° knee flexion<br>4 x 20s @ 70% MVC<br>60 s inter-rep rest	Stiffness: ramped iso knee extension MVC on isokinetic dynamometer, with 2D US to assess elongation of VL apon at 50% length of thigh visualising fascicle insertion into apon, calculated as the slope of linear regression over 50-100% MVC<br>Modulus: not assessed<br>CSA: T1 MRI, prone lying, immediate superior to patella and 10mm from patella	QT<br>VL Apon
Kubo et al., 2002 [111]	Resistance Training (extracted group)	8, 4	Unilat con:ecc PF on leg press<br>5 x 10 @ 70% 1RM	Stiffness: ramped iso PF MVC on isokinetic dynamometer, with 2D US 30% length of lower leg (proximal to distal) to assess displacement of GaM fascicle intersection with deep apon, calculated as the slope of linear regression over 50-100% MVC<br>Modulus: not assessed<br>CSA: T1 MRI, immediately superior to calcaneus and 10mm from calcaneus	AT
	Resistance training + static stretching		Exercises above + 5 x 45 s stretches for PF group		
Kubo et al., 2006 [112]	Isometric	12, 4	Bilat iso leg press<br>10 x 15s @ 70% MVC<br>60 s inter-set rest	Stiffness: ramped iso knee extension MVC on isokinetic dynamometer, with 2D US to assess elongation of VL apon at 50% length of thigh visualising fascicle insertion into apon and PT at apex of patella, calculated as the slope of linear regression over 50-100% MVC<br>Modulus: not assessed<br>CSA: T1 MRI; assessed 10, 20 and 30 mm inferior to patella	PT<br>VL Apon
	Control (rec active)		No exercise intervention		

Kubo et al., 2006 [113]	Short length limb	12, 4	Unilat iso knee extension<br>Short: 6 x 15s @ 50-70% MVC @ 50° knee flexion<br>30 s inter-rep rest	Stiffness: ramped iso knee extension MVC on isokinetic dynamometer, with 2D US to assess elongation of VL apon at 50% length of thigh visualising fascicle insertion into apon, calculated as the slope of linear regression over 50-100% MVC<br>Modulus: not assessed	QT<br>VL Apon
	Long length limb		Long: 6 x 15s @ 50-70% MVC @ 100° knee flexion<br>30 s inter-rep rest.	CSA: T1 MRI, prone lying, immediate superior to patella and 10mm from patella	
Kubo et al., 2006 [96]	High load (extracted group)	12, 3	Unilat con:ecc knee extension<br>0 - 90° knee flexion<br>4 x 10 @ 80% 1RM<br>Con = 1 s, Ecc = 3 s<br>Inter-set rest = 60 s	Stiffness: ramped iso knee extension MVC on isokinetic dynamometer, with 2D US to assess elongation of VL apon at 50% length of thigh visualising fascicle insertion into apon and PT at apex of patella, calculated as the slope of linear regression over 50-100% MVC<br>Modulus: not assessed<br>CSA: 2D US; assessed at 25, 50 and 75% length of the PT	PT<br>VL Apon
	BFR		Exercise above @ 20% 1RM<br>4 sets: 25/18/15/12 reps		
Kubo et al., 2007 [85]	Plyometric/ Jump limb	12, 4	Unilat Hop and DJ from 20cm;<br>Each exercise: 5 x 10 @ 40% 1RM PF<br>30 s inter-set rest	Stiffness: ramped iso PF MVC on isokinetic dynamometer, with 2D US to assess elongation at GaM MTJ, calculated as the slope of linear regression over 50-100% MVC<br>Modulus: not assessed	AT
	Weight training limb		Unilat con:ecc 5 x 10 PF @ 80% 1RM<br>Con = 1 s, Ecc = 3s<br>60 s inter-set rest	CSA: T1 MRI, immediately superior to calcaneus and 10mm from calcaneus	
Kubo et al., 2009 [114]	Isometric	12, 4	Unilat iso knee extension @ 90° knee flexion<br>10 x 15s @ 70% MVC<br>30 s inter-rep rest	Stiffness: ramped iso knee extension MVC on isokinetic dynamometer, with 2D US to assess elongation of VL apon at 50% length of thigh visualising fascicle insertion into apon and PT at apex of patella, calculated as the slope of linear regression over 50-100% MVC	PT<br>VL Apon
	Con:Ecc		Unilat con:ecc knee extension between 0-90°<br>5 x 10 @ 80% 1RM<br>Con = ~1s, Ecc = ~3s<br>60 s inter-set rest	Modulus: not assessed<br>CSA: 2D US; assessed at 25, 50 and 75% length of the PT	
Kubo et al., 2010 [115]	PF	12, 4	Unilat con:ecc PF<br>5 x 10 @ 80% 1RM<br>60 s inter-set rest<br>Con = 1 s, Ecc = 3 s	Stiffness: ramped iso knee extension and PF MVC on isokinetic dynamometer, with 2D US to assess elongation at the patella apex and GaM MTJ<br>Modulus: not assessed<br>CSA: T1 MRI; assessed immediately inferior to the patella and 20mm distal to patella, and immediately superior to calcaneus and 10mm superior to calcaneus	PT<br>AT
	Knee extension		Unilat con:ecc knee extension between 0-90°<br>5 x 10 @ 80% 1RM<br>Concentric = ~1 s, Eccentric: ~3 s<br>60 s inter-set rest		

Kubo et al., 2010 [88]	Isometric	12, 4	Unilat iso knee extension @ 90° knee flexion<br>10 x 15 s @ 70% MVC<br>30 s inter-rep rest	Stiffness: ramped iso knee extension MVC on isokinetic dynamometer, with 2D US to assess elongation of VL apon at 50% length of thigh visualising fascicle insertion into apon, calculated as the slope of linear regression over 50-100% MVC<br>Modulus: not assessed<br>CSA: T1 MRI;10, 20 and 30 mm inferior to patella	PT<br>VL Apon
	Control (rec active)		No exercise intervention		
Kubo et al., 2012 [89]	Isometric	12, 4	Unilat iso PF @ 0° DF (knee extended)<br>15 x 15 s @ 80% MVC<br>30 s inter-rep rest.	Stiffness: ramped iso PF MVC on isokinetic dynamometer, with 2D US to assess elongation at GaM MTJ, calculated as the slope of linear regression over 50-100% MVC<br>Modulus: not assessed<br>CSA: T1 MRI; assessed every three images	AT
	Control (rec active)		No exercise intervention		
Kubo et al., 2017 [86]	Isometric	12, 3	Unilat iso PF (prone lying) @ 0° DF (knee extended)<br>10 x 15 s @ 80% MVC<br>30 s inter-rep rest	Stiffness: ramped iso PF MVC on isokinetic dynamometer, with 2D US to assess elongation at GaM visualising fascicle intersection at deep apon at 30% lower leg length (proximal to distal), calculated as the slope of linear regression over 50-100% MVC<br>Modulus: not assessed<br>CSA: 2D US; level with lateral malleolus	AT
	Plyometric		Unilat hops/drop jumps on sled<br>5 x 10 @ 40%<br>30 s inter-set rest		
Kubo et al., 2017 [116]	Concentric<br>Eccentric	12, 3	Unilat con or ecc knee extn between 0-90° flexion<br>5 x 10 @ 80% 1RM<br>Con = 1 s (unloaded ecc = 3 s)<br>Ecc = 3 s (unloaded con = 1 s)<br>60 s inter-set rest	Stiffness: ramped iso knee extension MVC on isokinetic dynamometer, with 2D US to assess elongation between patella and tibial insertions, calculated as the slope of linear regression over 50-100% MVC<br>Modulus: not assessed<br>CSA: 2D US; assessed at 50% PT length	PT
	Control (rec active)		No exercise intervention		
Laurent et al., 2020 [117]	Knee extended<br>Knee flexed	10, 2	Bilat vertical hop and DJ variations (30-40cm)<br>6-8 exercises per session x 10 repetitions per exercise<br>~90 s inter-set rest<br>~180 s inter-exercise rest<br>200 → 400 foot contacts p/wk in either knee extended or flexed position	Stiffness: iso PF MVC using ankle ergometer with force transducer, with 2D US to assess elongation at GaM MTJ, calculated as slope of torque-elongation curve between 20-80% MVC<br>Modulus: not assessed<br>CSA: 2D US; 4cm superior to AT calcaneal insertion	AT
	Control (rec active)		No exercise intervention		

Malliaras et al., 2013 [61]	Concentric	12, 3	4 x 7-8 @ 80% con:ecc 1RM knee extension<br>Con phase = unilat, Ecc phase = bilat	Stiffness: iso knee extension MVC on isokinetic dynamometer, with 2D US to assess elongation at patella apex, calculated between 50-75% and 75-100% maximum torque<br>Modulus: calculated by multiplying stiffness between 50-75% and 75-100% MVC by ratio of tendon length to CSA<br>CSA: 2D US; 50% length of PT	PT
	Eccentric		4 x 12-15 @ 80% con:ecc 1RM knee extension<br>Con phase = bilat, Ecc phase = unilat<br>5 s ecc through 0-90° knee flexion		
	High load eccentric		4 x 7-8 @ 80% ecc 1RM knee extension<br>Con phase = bilat, Ecc phase = unilat<br>5 s ecc through 0-90° knee flexion		
	Control *		No exercise intervention		
Massey et al., 2018 [66]	Explosive contraction	12, 3	Unilat iso knee extension<br>Explosive: 4 x 10 @ >80% maximal torque<br>5 s inter-rep rest<br>120 s inter-set rest	Stiffness: ramped iso knee extension MVC using strain gauge, with 2D US to assess elongation of VL apon at 50% length of the thigh by visualising fascicle intersection with deep apon and of PT via displacement of patella and tibial insertions, calculated as slope of force-elongation curve over 70-80% MVT<br>Modulus: calculated as slope of stress-strain curve over stress range corresponding to 70-80% MVT<br>CSA: T1 MRI; contiguous images from 2cm superior to patella apex to 2cm inferior to tibial insertion	PT<br>VL apon
	Sustained contraction		Sustained: 4 x 10 @ 75% maximal torque<br>Contraction: 1 s ramp, 3 s plateau<br>2 s inter-rep rest<br>120 s inter-set rest		
	Control *		No exercise intervention – habitual exercise		
McMahon et al., 2013 [67]	Short range	8, 3	4 x con:ecc exercises p/session (2 x squat variations, 1 x machine, 1 x Sampson chair), from: exercises = barbell back squat, Bulgarian split squat, leg press, leg extension, dumbbell lunge, static Sampson chair<br>3 x 10 → 4 x 8<br>Short = 0-50° knee flexion @ 80% 1RM<br>Long range = 40-90° knee flexion @ 55% 1RM<br>Full range = 0-90° knee flexion @ 80% 1RM	Stiffness: ramped iso knee extension on isokinetic dynamometer, with 2D US to assess elongation at the patella apex, calculated as slope of force-elongation curve of 10% MVC intervals<br>Modulus: calculated as stiffness multiplied by the ratio of tendon length to CSA<br>CSA: 2D US; assessed at 25, 50 and 75% PT length	PT
	Long range				
	Full range				
	Control (rec active)		No exercise intervention – habitual exercise		
McMahon et al., 2018 [68]	Trained males	8, 3	4 x con:ecc exercises p/session (barbell back squat, Bulgarian split squat, leg press, leg extension, dumbbell lunge, static Sampson chair).<br>3 x 10 → 4 x 8 @ 80% 1RM	Stiffness: ramped iso knee extension on isokinetic dynamometer, with 2D US to assess elongation at the patella apex, calculated as the average stiffness value from 10-100% MVC<br>Modulus: calculated as stiffness multiplied by the ratio of tendon length to CSA<br>CSA: 2D US; assessed at 25, 50 and 75% PT length	PT
	Trained females				
	Control (males, females; rec active)		No exercise intervention		

Mouraux et al., 2000 [118]	Eccentric	6, 3	Unilat ecc DF on isokinetic dynamometer<br>3-6 x 10 @ 30-80% peak torque	Stiffness: not assessed<br>Modulus: not assessed<br>CSA: T2 MRI; assessed 2 cm proximally to calcaneal insertion	AT
	Control limb		Untrained contralateral limb		
Ogiso et al., 2020 [119]	Non-muscle stimulation (extracted group)	3, 3	3 x 10 reactive jump + 20 maximum effort reactive jumps	Stiffness: iso PF MVC on isokinetic dynamometer, with 2D US to assess elongation at GaM MTJ<br>Modulus: not assessed<br>CSA: not assessed	AT
	Electrical muscle stimulation		Exercises above + electrical muscle stimulation		
	Control (rec active)		No exercise intervention		
Onambélé et al., 2008 [120]	Resistance training	12, 3	Bilat con:ecc knee extension and ankle rotator<br>Resistance: 1-4 x 8-12 @ 80% 1RM	Stiffness: ramped iso PF MVC on isokinetic dynamometer, with 2D US to assess elongation (location n/s)<br>Modulus: not assessed<br>CSA: not assessed	AT
	Inertial flywheel training		Bilat con:ecc YOYO leg extension flywheel and ankle rotator<br>Flywheel: 1-4 x 8-12 @ 100% power output<br><br>5 min inter-set rest		
Quinlan et al., 2021 [121]	Young con<br><br>Young ecc<br><br>Old con<br><br>Old ecc	8, 3	Bilat ⟶ unilat con or ecc leg press<br>Con: 4 x 15 @ 60% Con 1RM<br>Ecc: 4 x 15 @ 60% Ecc 1RM<br>3 s contraction<br>120 s inter-set rest	Stiffness: ramped iso knee extension MVC on isokinetic dynamometer, with 2D US to assess elongation of PT between patella and tibial insertions, calculated as the gradient of force-elongation curve between 90-100% maximal force<br>Modulus: calculated as tendon stiffness multiplied by the ratio of tendon length to tend CSA<br>CSA: 3T MRI, every 1cm along length of PT	PT
Reeves et al., 2003 [69]	Training	14, 3	Bilat con:ecc leg press + leg extension (+ five other non-PT loading/general strength exercises)<br>2 x ~10 @ ~60-80% 5RM<br>Con = ~2s<br>Ecc = ~3s<br>~180 s inter-set rest	Stiffness: ramped iso knee extension MVC on isokinetic dynamometer, with 2D US to assess elongation of the PT, calculated as the gradient over 10% intervals of tendon force<br>Modulus: calculated as stiffness multiplied the ratio of tendon length to CSA<br>CSA: 2D US; 25, 50 and 75% of patella tendon length	PT
	Control *		No exercise intervention – habitual activity		

Reeves et al., 2003 [122]	Training	14, 3	Bilat con:ecc leg extension + leg press (+ five non-PT loading/general strength exercises)<br>2 x 10 @ ~60-80% 5RM<br>Con = ~2s<br>Ecc = ~3s.<br>~180 s inter-set rest	Stiffness: iso knee extension MVC on isokinetic dynamometer, with 2D US to assess elongation at patella apex, calculated as gradient of force-elongation curve between 60-100% MVC<br>Modulus: not assessed<br>CSA: not assessed	PT
	Control *		No exercise intervention – habitual activity		
Sanz-López et al., 2016 [123]	Eccentric overload	6, 2	Bilat con:ecc YoYo flywheel squats to parallel<br>4 x 7 @ 80% 1RM<br>120 s inter-set rest	Stiffness: not assessed<br>Modulus: not assessed<br>CSA: 2D US; 3cm proximal to calcaneal insertion	AT
	Control (rec active)		No resistance training intervention		
Seynnes et al., 2009 [70]	Training	9, 3	Unilat con:ecc knee extension (Technogym)<br>4 x 10 @ 80% 1RM<br>120 s inter-set rest	Stiffness: ramped iso knee extension on isokinetic dynamometer, with 2D US to assess elongation at patella apex<br>Modulus: calculated as tendon stiffness multiplied by ratio of tendon length to mean CSA<br>CSA: T1 MRI; assessed at 10% intervals	PT
Standley et al., 2013 [124]	Aerobic cycling	12, 3-4	20-45 min cyc @ 60-80% heart rate reserve @ 70-90 rpm<br>42 sessions total	Stiffness: not assessed<br>Modulus: not assessed<br>CSA: MRI; average of all slices	PT
Tillin et al., 2012 [125]	Trained limb	4, 4	Unilat iso explosive knee extension<br>4 x 10 @ ≥90% MVC<br>5 s inter-rep rest<br>120 s inter-set rest	Stiffness: ramped iso knee extension MVC via strain gauge, with 2D US to assess displacement of fascicle intersection at VL apon, calculated as slope of force-elongation curve between 10-50% and 50-90% MVC<br>Modulus: not assessed<br>CSA: not assessed	VL Apon
	Control limb		No exercise intervention		

Vikmoen et al., 2016 [59]	Cc Endurance + strength (extracted group)<br><br>Endurance (habitual)	11, 2	Strength: Smith machine half squat, unilat leg press, unilat cable hip flexion, calf raises<br>3 x 10RM ⟶ 4RM (each exercise)<br>Con = ~1 s<br>Ecc = 2-3 s<br><br>Endurance training (cyc/R, not prescribed): completed on separate day<br>~ 4 x sessions/wk<br>60-100% heart rate	Stiffness: ramped iso knee extension MVC via force cell, with 2D US to assess displacement of patellar apex relative to tibial plateau; calculated the slope of force-elongation curves between 90-100% MVC<br>Modulus: calculated as stiffness multiplied by ratio of patella length and mean CSA<br>CSA: 2D US; assessed at proximal, middle and distal regions	PT
Wakahara et al., 2015 [75]	Training<br><br>Control (sedentary/rec active)	12, 3	Unilat con:ecc knee extension @ 20-100° knee flexion<br>5 x 8 @ 80% 1RM<br>Con = 2s<br>Ecc = 2s<br>90 s inter-set rest<br><br>No exercise intervention – habitual activity	Stiffness: not assessed<br>Modulus: not assessed<br>CSA: T1 MRI; mean value from all slices with visible apon	VL apon
Walker et al., 2020 [58]	Traditional training<br><br>Accentuated eccentric training<br><br>Control (active)	10, 2	Bilat con:ecc leg press, unilat knee extension, bilat knee flexion<br>Each wk: Session 1 = 3 x 6RM, session 2 = 3 x 10RM<br><br>Accentuated ecc = con load + 40%<br><br>Concentric = 2s, Eccentric = 2s<br><br>No exercise intervention – own resistance training	Stiffness: ramped iso knee extension on custom dynamometer, with 2D US to assess elongation between patellar apex and tibial insertion, calculated as the slope of force-elongation curve from 50-100% MVC<br>Modulus: not assessed<br>CSA: not assessed	PT
Waugh et al., 2014 [74]	Training<br><br>Control *	10, 2	Circuit of team-based activity + 1 x station with 45° con:ecc incline calf raise.<br>2 x 8-15 RM ⟶ 3 x 8-15RM<br><br>No exercise intervention	Stiffness: iso PF on isokinetic dynamometer, with 2D US to assess displacement of GaM MTJ, calculated as the slope of linear force-elongation curve between 10-90% MVC<br>Modulus: calculated as slope of stress-strain relationship between 10-90% peak stress<br>CSA: 2D US; assessed ~25 mm from proximal calcaneus	AT
Waugh et al., 2018 [54]	Long rest<br><br>Short rest	12, 3	Unilat iso PF<br>5 x 10 x 3 s @ 90% MVC<br>Long: 10 s inter-rep rest<br>Short: 3 s inter-rep rest<br>90 s inter-set rest	Stiffness: ramped iso PF on isokinetic dynamometer, with 2D US to assess displacement of GaM MTJ, calculated as the slope of the linear force-alongation curve between 25-90% MVC<br>Modulus: calculated as the slope of the stress-strain curve between 25-90% peak stress<br>CSA: 3D UTC transverse images at 1, 2, 3 and 4 cm proximal to tendon insertion	AT

Waugh et al., 2021 [55]	Long rest<br><br>Short rest	12, 3	Unilat iso PF<br>5 x 10 x 3 s @ 90% MVC<br>Long: 10 s inter-rep rest<br>Short: 3 s inter-rep rest<br>90 s inter-set rest	Stiffness: ramped iso PF on isokinetic dynamometer, with 2D US to assess displacement of GaM MTJ, calculated as the slope of the linear force-alongation curve between 25-90% MVC<br>Modulus: calculated as the slope of the stress-strain curve between 25-90% peak stress<br>CSA: 3D UTC transverse images at 1, 2, 3 and 4 cm proximal to tendon insertion	AT
Werkhausen et al., 2018 [56]	Isometric	10, 3	Unilat iso PF in standing<br>4 x 10 explosive (~1 s) @ 80% MVC<br>5 s inter-rep rest	Stiffness: ramped iso PF on isokinetic dynamometer, with 2D US to assess displacement at GaM MTJ, calculated as the slope of the force-elongation curve between 50-80% MVC<br>Modulus: not assessed	AT
	Control (rec active)		No exercise intervention – habitual exercise	CSA: not assessed	
Werkhausen et al., 2019 [57]	Training	10, 3	Unilat iso PF in standing<br>4 x 10 explosive (~1 s) @ 80% MVC<br>5 s inter-rep rest	Stiffness: ramped iso PF on isokinetic dynamometer, with 2D US to assess displacement at GaM MTJ, calculated as the slope of the force-elongation curve between 50-80% MVC<br>Modulus: not assessed	AT
	Control (rec active)		Control (rec active)	CSA: not assessed	
Wu et al., 2010 [126]	Training/Jump	8, 2	Wk 1-2 (low intensity): SJ (10x2); split SJ (10x2); cycled split SJ (10x2)<br>Wk 3-4 (low/med): Split SJ (10x2); pike jump (10x2); double leg tuck jump (10x2)<br>Wk 5-6 (med): Pike jump (10x3); double leg tuck jump (10x3); double leg zigzag hop (10x3); double leg hop (10x3)<br>Wk 7-8 (med/high): Double leg zigzag hop (10x3); double leg hop (10x3); depth jump (10x3); box jump (10x3)<br>30s inter-set rest<br>120s inter-exercise rest<br>Box height = 45cm	Stiffness: ramped iso PF MVC via load cell, with 2D US to assess displacement of GaM MTJ, calculated as slope of ascending phase of muscle contraction between 60-100% MVC (on stress-displacement loop)<br>Modulus: not assessed<br>CSA: not assessed	AT
	Control *		General stretch activity for upper limb and back, 2 x p/wk		

Notes/abbreviations: AT = Achilles tendon; Bilat = bilateral; Cc = concurrent training; Con = concentric; Con:Ecc = concentric:eccentric; Cyc = cycling; DF = dorsiflexion; Ecc = eccentric; CMJ = countermovement jump; DJ = drop jump; F = female; GaM Apon = Gastronemius medialis aponeurosis; GRF = ground reaction force; Iso = isometric; M = Male; min = minute; MRI = magnetic resonance imaging; MTJ = myo/musculotendinous junction; MVC = maximal voluntary contraction; MVT = maximal voluntary torque; n/s = not specified; PF = plantarflexion; PT = patellar tendon; QT = Quadriceps tendon; R = running; rec active = recreationally active; rpm = revolutions per minute; SJ = squat jump; SSC = stretch shortening cycle; Unilat = unilateral; UTC = ultrasound tissue characterisation; VL Apon = Vastus lateralis aponeurosis; Wk = week(s); 2D US = two-dimensional ultrasound; nRM = repetition maximum of n; n x p/wk = number of sessions per week; * = healthy control participants, no activity status (i.e., not active, recreationally active, trained athlete) not specified; → = progressing to

S6. Quality analysis using the PEDro scale, showing individual criteria scores, total score and adjusted relative score (i.e., using number of criteria applicable to study design as the denominator).

Author	Criteria											Total	Relative score %
	1	2	3	4	5	6	7	8	9	10	11		
Albracht et al., 2013 [5]	*1*	0	0	0	-	-	0	1	1	0	1	3	38
Arampatzis et al. 2007 [19]	*0*	1	0	1	-	-	0	1	1	1	1	6	75
Arampatzis et al. 2010 [20]	*0*	1	0	1	-	-	0	1	1	1	1	6	75
Baptista et al. 2016 [95]	*0*	1	0	1	-	-	1	1	0	1	1	6	75
Bohm et al. 2014 [21]	*0*	1	0	1	-	-	0	1	1	1	1	6	75
Bohm et al., 2021 [17]	*1*	1	0	1	-	-	0	0	1	1	1	5	62.5
Carroll et al. 2011 [62]	*1*	1	0	0	-	-	1	1	0	1	1	5	63
Centner et al., 2019 [71]	*1*	1	1	1	-	-	1	0	1	1	1	7	88
Dalgaard et al. 2019 [104]	*1*	0	0	1	-	-	1	1	1	0	1	5	63
Duclay et al., 2009 [105]	*1*	0	0	1	-	-	0	1	1	0	1	4	50
Eriksen et al. 2018 [63]	*1*	1	0	0	-	-	1	0	1	1	1	5	63
Eriksen et al. 2019 [64]	*1*	1	1	1	-	-	0	1	1	1	1	7	88
Farup et al., 2014 [106]	*1*	0	0	1	-	-	1	0	1	1	1	5	63
Fletcher et al., 2010 [60]	*1*	1	0	1	-	-	0	1	1	1	1	6	75
Fouré et al. 2009 [107]	*0*	1	0	1	-	-	0	0	0	0	1	3	38
Fouré et al. 2010 [49]	*0*	1	0	1	-	-	0	1	1	0	1	5	63
Fouré et al. 2011 [50]	*0*	1	0	1	-	-	0	0	0	0	1	3	38
Fouré et al. 2012 [51]	*0*	0	0	1	-	-	0	1	1	0	1	4	50
Fouré et al. 2013 [108]	*0*	1	0	1	-	-	0	0	0	0	1	3	38

Geremia et al., 2018 [72] *	*1*	-	-	-	-	-	0	0	1	-	1	2	50
Hirayama et al. 2017 [109]	*0*	1	0	1	-	-	0	0	0	0	1	3	38
Houghton et al., 2013 [73]	*1*	0	0	0	-	-	0	0	0	1	1	2	25
Kay et al. 2016 [110] *	*0*	-	-	-	-	-	0	0	0	-	1	1	25
Kongsgaard et al. 2007 [65]	*0*	1	0	1	-	-	1	0	0	1	1	5	63
Kubo et al. 2001 [51]	*0*	1	0	1	-	-	0	0	0	1	1	4	50
Kubo et al. 2001 [52] *	*0*	-	-	-	-	-	0	0	0	-	1	1	25
Kubo et al. 2002 [111]	*0*	1	0	1	-	-	0	0	0	1	1	4	50
Kubo et al. 2006 [112]	*0*	0	0	1	-	-	0	1	1	1	1	5	63
Kubo et al. 2006 [113]	*0*	1	0	1	-	-	0	0	0	1	1	4	50
Kubo et al., 2006 [96]	*0*	1	0	1	-	-	0	0	0	1	1	4	50
Kubo et al. 2007 [85]	*0*	1	0	0	-	-	0	0	0	0	1	2	25
Kubo et al. 2009 [114]	*0*	1	0	1	-	-	0	0	0	0	1	3	38
Kubo et al., 2010 [115]	*0*	1	0	1	-	-	0	0	0	1	1	4	50
Kubo et al. 2010 [88]	*0*	1	0	0	-	-	0	1	1	0	1	4	50
Kubo et al. 2012 [89]	*0*	1	0	0	-	-	0	0	0	0	1	2	25
Kubo et al., 2017 [86]	*0*	1	0	1	-	-	0	0	0	1	1	4	50
Kubo et al., 2017 [116]	*0*	1	0	0	-	-	0	0	0	0	1	2	25
Laurent et al. 2020 [117]	*0*	1	0	1	-	-	0	1	1	1	1	6	75
Malliaras et al. 2013 [61]	*1*	1	1	1	-	-	1	1	1	1	1	8	100
Massey et al. 2018 [66]	*0*	1	0	1	-	-	0	1	1	1	1	6	75
McMahon et al. 2013 [67]	*1*	1	0	0	-	-	0	0	0	1	1	3	38
McMahon et al. 2018 [68]	*1*	1	0	1	-	-	0	1	1	1	1	6	75

Mouraux et al., 2000 [118]	*1*	0	0	1	-	-	0	0	0	1	1	3	38
Ogiso et al., 2020 [119]	*1*	1	0	1	-	-	0	0	0	0	1	3	38
Onambélé et al., 2008 [120]	*0*	1	0	1	-	-	0	1	1	1	1	6	75
Quinlan et al., 2021 [121]	*1*	1	0	1	-	-	0	1	1	1	1	6	75
Reeves et al. 2003 [69]	*0*	1	0	1	-	-	0	0	0	0	1	3	38
Reeves et al. 2003 [122]	*0*	1	0	1	-	-	0	1	1	0	1	5	63
Sanz-López et al. 2016 [123]	*1*	0	0	1	-	-	1	1	1	0	1	4	50
Seynnes et al. 2009 [70] *	*1*	-	-	-	-	-	0	0	0	-	1	1	25
Standley et al., 2013 [124] *	*0*	-	-	-	-	-	1	0	1	-	1	3	75
Tillin et al., 2012 [125]	*1*	1	0	1	-	-	0	1	0	1	1	5	63
Vikmoen et al., 2016 [59]	*1*	1	0	1	-	-	0	0	0	1	1	4	50
Wakahara et al., 2015 [75]	*0*	1	0	1	-	-	0	0	0	0	1	3	38
Walker et al., 2020 [58]	*1*	0	0	0	-	-	0	0	0	1	1	2	25
Waugh et al. 2014 [74]	*0*	1	0	1	-	-	0	1	1	1	1	6	75
Waugh et al. 2018 [54]	*0*	1	1	1	-	-	1	0	1	1	1	7	88
Waugh et al., 2021 [55]	*1*	1	0	1	-	-	0	0	0	1	1	4	50
Werkhausen et al. 2018 [56]	*0*	0	0	1	-	-	0	0	0	1	1	3	38
Werkhausen et al., 2019 [57]	*1*	0	0	1	-	-	0	0	0	1	1	3	38
Wu et al. 2010 [126]	*1*	1	0	1	-	-	0	1	1	0	1	5	63
	43	79	7	82	-	-	18	41	48	64	100		

Note: Relative score = total score/maximum possible score for the study design (Multiple group, experimental = 8, Single group = 4). Dash/hyphen denotes criterion is not applicable for study design. PEDro score does not use Criterion 1 for calculating total and subsequently is also not included in the calculation of the relative score. Studies with a single group (*) cannot achieve criteria 2-4 or 10 which have also been removed from

calculation of the relative score for those papers and the percentage of papers meeting the criteria. ITT = Intention to treat; Criteria: 1) Eligibility criteria specified; 2) Random allocation to groups; 3) Allocation concealment ; 4) Groups are similar at baseline; 5) Blinding of subjects to allocation/condition; 6) Blinding of therapist delivering condition; 7) Blinding of assessor of key outcome; 8) Key outcome recorded for >85% of participants; 9) All subjects received the condition, or an intention to treat analysis was used; 10) Between-groups statistics documented; 11) Point measures and variability provided for outcomes.

S7. Forest plot for the meta-analysis of stiffness subdivided by training intervention type showing standardised mean differences (SMD) and 95% confidence intervals (CI).

	Post			Pre					
**Study**	**n**	**Mean**	**SD**	**n**	**Mean**	**SD**	**SMD**	**[95% CI]**	**Weight**
Concurrent training									
Fletcher et al. 2010 [60]	6	434	122	6	366	56	0.66	[-0.52; 1.84]	0.9%
Vikmoen et al. 2016 [59]	11	2483	733	11	2752	402	-0.44	[-1.29; 0.41]	1.4%
Random effects model	17			17			0.03	[-1.03; 1.10]	2.2%
Heterogeneity: I^2 = 55%, p < 0.01									
Jump-based training									
Bohm et al. 2014 [21]	14	475	254	14	339	114	0.67	[-0.10; 1.43]	1.6%
Foure et al. 2009 [107]	6	43	18	6	41	3	0.12	[-1.01; 1.26]	0.9%
Foure et al. 2010 [49]	9	283	137	9	223	87	0.50	[-0.44; 1.44]	1.2%
Hirayama et al. 2017 [109]	8	260	67	8	193	52	1.06	[-0.01; 2.12]	1.0%
Houghton et al. 2013 [73]	7	856	564	7	940	473	-0.15	[-1.20; 0.90]	1.0%
Kubo et al. 2007 [85]	10	154	55	10	129	36	0.51	[-0.38; 1.41]	1.3%
Kubo et al. 2017 [86]	11	23	5	11	23	6	0.12	[-0.71; 0.96]	1.4%
Laurent et al. 2020 [117]	11	7	2	11	5	1	0.89	[0.01; 1.78]	1.3%
Laurent et al. 2020 [117]	11	6	2	11	5	2	0.41	[-0.43; 1.26]	1.4%
Ogiso et al. 2020 [119]	9	17	5	9	15	3	0.43	[-0.50; 1.37]	1.2%
Wu et al. 2010 [126]	11	80	35	11	56	32	0.68	[-0.19; 1.54]	1.3%
Random effects model	107			107			0.49	[0.22; 0.77]	13.5%
Heterogeneity: I^2 = 0%, p = 0.90									
Resistance training									
Albracht et al. 2013 [5]	13	315	53	13	272	48	0.82	[0.02; 1.63]	1.5%
Arampatzis et al. 2007 [19]	11	201	41	11	187	38	0.36	[-0.49; 1.20]	1.4%
Arampatzis et al. 2007 [19]	11	228	40	11	168	37	1.52	[0.55; 2.49]	1.1%
Arampatzis et al. 2010 [20]	11	261	56	11	276	53	-0.25	[-1.09; 0.59]	1.4%
Arampatzis et al. 2010 [20]	11	302	57	11	258	51	0.79	[-0.09; 1.66]	1.3%
Bohm et al. 2014 [21]	12	457	132	12	370	132	0.64	[-0.18; 1.47]	1.4%
Bohm et al. 2014 [21]	14	539	254	14	336	89	1.04	[0.24; 1.84]	1.5%
Bohm et al. 2014 [21]	12	579	172	12	377	106	1.37	[0.46; 2.27]	1.3%
Bohm et al. 2021 [17]	13	111	59	13	85	36	0.52	[-0.27; 1.30]	1.5%
Carroll et al. 2011 [62]	12	3335	1334	12	2928	1320	0.30	[-0.51; 1.10]	1.5%
Centner et al. 2019 [71]	14	565	158	14	402	103	1.19	[0.38; 2.01]	1.4%
Duclay et al. 2009 [105]	10	291	125	10	249	90	0.38	[-0.51; 1.26]	1.3%
Eriksen et al. 2018 [63]	9	3890	2430	9	2560	1530	0.62	[-0.33; 1.58]	1.2%
Eriksen et al. 2018 [63]	12	1900	693	12	1800	624	0.15	[-0.65; 0.95]	1.5%
Eriksen et al. 2019 [64]	10	4420	1075	10	4060	1360	0.28	[-0.60; 1.16]	1.3%
Foure et al. 2013 [108]	11	251	109	11	216	55	0.39	[-0.45; 1.24]	1.4%
Geremia et al. 2018 [72]	15	350	59	15	192	36	3.15	[2.03; 4.26]	0.9%
Kay et al. 2016 [110]	13	13	3	13	10	2	1.10	[0.27; 1.94]	1.4%
Kongsgaard et al. 2007 [65]	12	4213	1406	12	3676	1306	0.38	[-0.43; 1.19]	1.5%
Kongsgaard et al. 2007 [65]	12	3375	1251	12	3716	1569	-0.23	[-1.04; 0.57]	1.5%
Kubo et al. 2001 [51]	8	106	33	8	68	21	1.31	[0.20; 2.42]	0.9%
Kubo et al. 2002 [111]	8	34	10	8	26	9	0.80	[-0.23; 1.83]	1.0%
Kubo et al. 2006 [112]	8	1786	660	8	1790	559	-0.01	[-0.99; 0.97]	1.1%
Kubo et al. 2006 [112]	8	59	24	8	51	22	0.29	[-0.69; 1.28]	1.1%
Kubo et al. 2006 [113]	9	86	36	9	79	21	0.25	[-0.68; 1.17]	1.2%
Kubo et al. 2006 [113]	9	122	40	9	81	26	1.17	[0.15; 2.18]	1.1%
Kubo et al. 2006 [96]	9	1819	710	9	1676	662	0.20	[-0.73; 1.13]	1.2%
Kubo et al. 2006 [96]	9	59	23	9	46	19	0.62	[-0.33; 1.57]	1.2%
Kubo et al. 2007 [85]	10	166	44	10	128	26	1.01	[0.07; 1.96]	1.2%
Kubo et al. 2009 [114]	10	1833	892	10	1071	639	0.94	[0.01; 1.88]	1.2%
Kubo et al. 2009 [114]	10	1253	410	10	999	426	0.58	[-0.32; 1.48]	1.3%
Kubo et al. 2009 [114]	10	110	36	10	67	19	1.43	[0.43; 2.44]	1.1%
Kubo et al. 2009 [114]	10	96	37	10	72	20	0.75	[-0.16; 1.67]	1.2%
Kubo et al. 2010 [115]	10	41	13	10	32	5	0.85	[-0.08; 1.77]	1.2%
Kubo et al. 2010 [115]	10	96	37	10	72	20	0.75	[-0.16; 1.67]	1.2%
Kubo et al. 2010 [88]	8	104	37	8	69	19	1.12	[0.04; 2.20]	1.0%
Kubo et al. 2012 [89]	9	277	111	9	183	45	1.06	[0.05; 2.06]	1.1%
Kubo et al. 2017 [116]	9	1414	355	9	1004	384	1.06	[0.05; 2.06]	1.1%
Kubo et al. 2017 [116]	9	1457	440	9	1289	544	0.32	[-0.61; 1.26]	1.2%
Kubo et al. 2017 [86]	11	32	9	11	24	5	1.17	[0.25; 2.09]	1.2%
Malliaras et al. 2013 [61]	9	2338	638	9	1560	793	1.03	[0.03; 2.03]	1.1%
Malliaras et al. 2013 [61]	10	2536	850	10	1822	898	0.78	[-0.13; 1.70]	1.2%
Malliaras et al. 2013 [61]	10	2508	1066	10	1387	360	1.35	[0.36; 2.34]	1.1%
Massey et al. 2018 [66]	14	3122	632	14	2605	446	0.92	[0.13; 1.70]	1.5%
Massey et al. 2018 [66]	15	3239	575	15	2835	444	0.77	[0.02; 1.51]	1.6%
Massey et al. 2018 [66]	14	595	101	14	592	118	0.03	[-0.71; 0.77]	1.6%
Massey et al. 2018 [66]	15	687	285	15	560	177	0.52	[-0.21; 1.25]	1.6%
McMahon et al. 2013 [67]	10	1221	594	10	916	441	0.56	[-0.34; 1.46]	1.3%
McMahon et al. 2013 [67]	11	1124	471	11	837	379	0.65	[-0.22; 1.51]	1.3%
McMahon et al. 2013 [67]	11	1167	353	11	765	242	1.28	[0.34; 2.21]	1.2%
McMahon et al. 2018 [68]	8	1517	390	8	1132	294	1.05	[-0.01; 2.12]	1.0%
McMahon et al. 2018 [68]	8	887	305	8	619	204	0.98	[-0.08; 2.03]	1.0%
Onambélé et al. 2008 [120]	12	40	23	12	26	13	0.72	[-0.11; 1.55]	1.4%
Onambélé et al. 2008 [120]	12	59	22	12	25	6	2.06	[1.04; 3.09]	1.1%
Quinlan et al. 2021 [121]	8	2314	466	8	1267	260	2.62	[1.19; 4.06]	0.6%
Quinlan et al. 2021 [121]	10	2377	608	10	1769	462	1.08	[0.13; 2.03]	1.2%
Quinlan et al. 2021 [121]	9	2512	635	9	1782	570	1.15	[0.13; 2.17]	1.1%
Quinlan et al. 2021 [121]	8	2326	769	8	1376	428	1.44	[0.31; 2.58]	0.9%
Reeves et al. 2003 [122]	7	2256	1476	7	1376	811	0.69	[-0.40; 1.78]	1.0%
Reeves et al. 2003 [69]	9	3610	1220	9	2187	713	1.36	[0.30; 2.41]	1.0%
Seynnes et al. 2009 [70]	15	2288	2115	15	1864	1813	0.21	[-0.51; 0.93]	1.7%
Tillin et al. 2012 [125]	10	697	103	10	520	86	1.79	[0.71; 2.86]	1.0%
Walker et al. 2020 [58]	10	1893	687	10	1981	597	-0.13	[-1.01; 0.75]	1.3%
Walker et al. 2020 [58]	10	1582	546	10	1437	304	0.31	[-0.57; 1.20]	1.3%
Waugh et al. 2014 [74]	10	178	32	10	138	37	1.10	[0.14; 2.05]	1.2%
Waugh et al. 2018 [54]	14	456	49	14	390	35	1.51	[0.66; 2.37]	1.4%
Waugh et al. 2018 [54]	14	461	40	14	359	53	2.11	[1.16; 3.06]	1.2%
Werkhausen et al. 2018 [56]	11	459	147	11	397	146	0.41	[-0.44; 1.25]	1.4%
Random effects model	726			726			0.80	[0.67; 0.94]	84.2%
Heterogeneity: I^2 = 35%, p = 0.14									
**Random effects model**	**850**			**850**			**0.74**	**[0.62; 0.86]**	**100.0%**
Heterogeneity: I^2 = 33%, p < 0.01									
Residual heterogeneity: I^2 = 29%, p < 0.01									

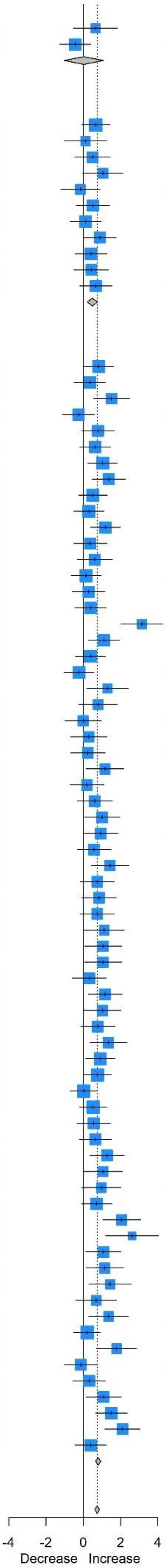

S8. Forest plot for the meta-analysis of modulus subdivided by training intervention type showing standardised mean differences (SMD) and 95% confidence intervals (CI).

Study	Post n	Post Mean	Post SD	Pre n	Pre Mean	Pre SD	SMD	[95% CI]	Weight
**Concurrent training**									
Vikmoen et al. 2016 [59]	11	925	162	11	1038	194	-0.61	[-1.47; 0.25]	2.8%
Random effects model	11			11			-0.61	[-1.47; 0.25]	2.8%
Heterogeneity: not applicable									
**Jump-based training**									
Bohm et al. 2014 [21]	14	1140	486	14	920	299	0.53	[-0.23; 1.28]	3.0%
Houghton et al. 2013 [73]	7	2000	1300	7	2500	1300	-0.36	[-1.42; 0.70]	2.4%
Random effects model	21			21			0.17	[-0.69; 1.02]	5.4%
Heterogeneity: $I^2 = 44\%$, $p = 0.18$									
**Resistance training**									
Arampatzis et al. 2007 [19]	11	400	293	11	420	401	-0.05	[-0.89; 0.78]	2.9%
Arampatzis et al. 2007 [19]	11	680	355	11	440	278	0.72	[-0.14; 1.59]	2.8%
Arampatzis et al. 2010 [20]	11	970	265	11	1010	199	-0.16	[-1.00; 0.67]	2.9%
Arampatzis et al. 2010 [20]	11	1130	332	11	970	232	0.54	[-0.32; 1.39]	2.8%
Bohm et al. 2014 [21]	12	1050	277	12	890	277	0.56	[-0.26; 1.38]	2.9%
Bohm et al. 2014 [21]	14	1430	636	14	910	262	1.04	[0.24; 1.83]	2.9%
Bohm et al. 2014 [21]	12	1410	381	12	970	277	1.28	[0.38; 2.17]	2.7%
Carroll et al. 2011 [62]	12	1220	450	12	1030	416	0.42	[-0.39; 1.23]	2.9%
Centner et al. 2019 [71]	14	1848	481	14	1540	492	0.61	[-0.15; 1.38]	3.0%
Eriksen et al. 2018 [63]	9	1330	690	9	1230	390	0.17	[-0.76; 1.10]	2.7%
Eriksen et al. 2018 [63]	12	735	263	12	703	253	0.12	[-0.68; 0.92]	2.9%
Eriksen et al. 2019 [64]	10	1560	443	10	1510	474	0.10	[-0.77; 0.98]	2.8%
Geremia et al. 2018 [72]	15	1292	436	15	695	160	1.77	[0.91; 2.63]	2.8%
Kongsgaard et al. 2007 [65]	12	1650	554	12	1470	589	0.30	[-0.50; 1.11]	2.9%
Kongsgaard et al. 2007 [65]	12	1360	658	12	1420	727	-0.08	[-0.88; 0.72]	2.9%
Kubo et al. 2001 [52]	8	433	35	8	288	26	4.45	[2.42; 6.47]	1.1%
Malliaras et al. 2013 [61]	9	942	279	9	620	223	1.22	[0.19; 2.24]	2.4%
Malliaras et al. 2013 [61]	10	1022	339	10	737	390	0.75	[-0.17; 1.66]	2.7%
Malliaras et al. 2013 [61]	10	1011	436	10	570	191	1.25	[0.28; 2.23]	2.5%
Massey et al. 2018 [66]	14	1490	270	14	1230	180	1.10	[0.30; 1.90]	2.9%
Massey et al. 2018 [66]	15	1510	360	15	1320	270	0.58	[-0.15; 1.31]	3.1%
McMahon et al. 2013 [67]	10	1100	120	10	830	90	2.44	[1.22; 3.65]	2.1%
McMahon et al. 2013 [67]	11	990	110	11	740	90	2.39	[1.25; 3.54]	2.2%
McMahon et al. 2013 [67]	11	1150	110	11	780	100	3.39	[2.00; 4.77]	1.8%
McMahon et al. 2018 [68]	8	990	255	8	780	170	0.92	[-0.13; 1.96]	2.4%
McMahon et al. 2018 [68]	8	600	226	8	420	113	0.95	[-0.10; 2.00]	2.4%
Quinlan et al. 2021 [121]	8	1280	230	8	710	130	2.88	[1.37; 4.40]	1.6%
Quinlan et al. 2021 [121]	10	1510	440	10	1160	290	0.90	[-0.03; 1.83]	2.6%
Quinlan et al. 2021 [121]	9	1460	350	9	1050	270	1.25	[0.22; 2.28]	2.4%
Quinlan et al. 2021 [121]	8	1430	570	8	800	250	1.35	[0.23; 2.47]	2.3%
Reeves et al. 2003 [69]	9	2200	800	9	1300	300	1.42	[0.36; 2.48]	2.4%
Seynnes et al. 2009 [70]	15	1160	1201	15	980	1162	0.15	[-0.57; 0.87]	3.1%
Waugh et al. 2014 [74]	10	799	150	10	642	171	0.94	[0.00; 1.87]	2.6%
Waugh et al. 2018 [54]	14	1586	611	14	1261	459	0.58	[-0.18; 1.34]	3.0%
Waugh et al. 2018 [54]	14	1529	459	14	1242	420	0.63	[-0.13; 1.39]	3.0%
Random effects model	389			389			0.90	[0.65; 1.15]	91.8%
Heterogeneity: $I^2 = 61\%$, p = NA									
**Random effects model**	**421**			**421**			**0.82**	**[0.58; 1.07]**	**100.0%**
Heterogeneity: $I^2 = 63\%$, $p < 0.01$									
Residual heterogeneity: $I^2 = 61\%$, $p < 0.01$									

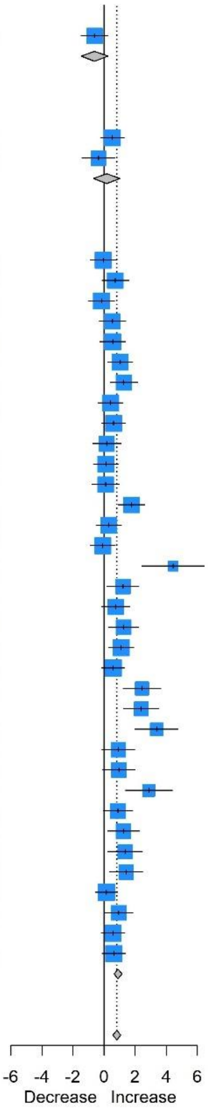

S9. Forest plot for the meta-analysis of cross-sectional area subdivided by training type showing standardised mean differences (SMD) and 95% confidence intervals (CI).

Study	n (Post)	Mean (Post)	SD (Post)	n (Pre)	Mean (Pre)	SD (Pre)	SMD	[95% CI]	Weight
**Aerobic training**									
Standley et al. 2013 [124]	9	92	18	9	90	15	0.11	[-0.81; 1.04]	1.2%
Random effects model	9			9			0.11	[-0.81; 1.04]	1.2%
Heterogeneity: not applicable									
**Concurrent training**									
Vikmoen et al. 2016 [59]	11	69	7	11	66	7	0.45	[-0.40; 1.30]	1.4%
Random effects model	11			11			0.45	[-0.40; 1.30]	1.4%
Heterogeneity: not applicable									
**Jump-based training**									
Bohm et al. 2014 [21]	14	82	13	14	80	15	0.14	[-0.60; 0.88]	1.9%
Foure et al. 2010 [49]	9	57	13	9	56	12	0.13	[-0.80; 1.05]	1.2%
Houghton et al. 2013 [73]	7	79	8	7	70	7	1.12	[-0.04; 2.28]	0.8%
Kubo et al. 2007 [85]	10	59	9	10	57	9	0.20	[-0.67; 1.08]	1.3%
Kubo et al. 2017 [86]	11	65	7	11	66	8	-0.13	[-0.97; 0.71]	1.5%
Laurent et al. 2020 [117]	11	62	11	11	62	11	0.00	[-0.84; 0.84]	1.5%
Laurent et al. 2020 [117]	11	56	10	11	56	10	0.00	[-0.84; 0.84]	1.5%
Random effects model	73			73			0.14	[-0.19; 0.47]	9.7%
Heterogeneity: $I^2 = 0\%$, p = NA									
**Resistance training**									
Arampatzis et al. 2007 [19]	11	53	15	11	50	15	0.14	[-0.70; 0.98]	1.5%
Arampatzis et al. 2007 [19]	11	53	14	11	48	13	0.30	[-0.55; 1.14]	1.5%
Arampatzis et al. 2010 [20]	11	60	35	11	58	34	0.05	[-0.79; 0.88]	1.5%
Arampatzis et al. 2010 [20]	11	56	36	11	56	38	0.02	[-0.82; 0.85]	1.5%
Baptista et al. 2016 [95]	23	10	1	23	9	1	0.82	[0.22; 1.42]	2.9%
Baptista et al. 2016 [95]	23	10	2	23	9	3	0.21	[-0.37; 0.79]	3.1%
Bohm et al. 2014 [21]	12	82	12	12	78	11	0.36	[-0.45; 1.16]	1.6%
Bohm et al. 2014 [21]	14	83	14	14	80	13	0.23	[-0.52; 0.97]	1.9%
Bohm et al. 2014 [21]	12	79	10	12	75	9	0.34	[-0.47; 1.14]	1.6%
Carroll et al. 2011 [62]	12	115	33	12	117	35	-0.06	[-0.86; 0.74]	1.6%
Centner et al. 2019 [71]	14	74	17	14	70	18	0.18	[-0.56; 0.92]	1.9%
Dalgaard et al. 2019 [104]	14	97	23	14	86	17	0.54	[-0.22; 1.29]	1.8%
Eriksen et al. 2018 [63]	9	129	24	9	131	27	-0.07	[-1.00; 0.85]	1.2%
Eriksen et al. 2018 [63]	11	123	33	12	121	28	0.06	[-0.76; 0.88]	1.6%
Eriksen et al. 2019 [64]	10	124	22	10	117	21	0.31	[-0.57; 1.20]	1.3%
Farup et al. 2014 [106]	11	149	22	11	140	16	0.44	[-0.41; 1.29]	1.5%
Farup et al. 2014 [106]	11	149	19	11	138	20	0.56	[-0.30; 1.42]	1.4%
Foure et al. 2013 [108]	11	67	10	11	68	7	-0.11	[-0.94; 0.73]	1.5%
Geremia et al. 2018 [72]	15	72	11	15	62	8	1.01	[0.24; 1.77]	1.8%
Kongsgaard et al. 2007 [65]	12	121	14	12	117	14	0.28	[-0.53; 1.08]	1.6%
Kongsgaard et al. 2007 [65]	12	118	13	12	116	18	0.10	[-0.70; 0.91]	1.6%
Kubo et al. 2001 [51]	8	213	19	8	210	16	0.16	[-0.82; 1.14]	1.1%
Kubo et al. 2001 [51]	8	215	21	8	212	18	0.15	[-0.84; 1.13]	1.1%
Kubo et al. 2002 [111]	8	59	8	8	61	9	-0.22	[-1.21; 0.76]	1.1%
Kubo et al. 2006 [112]	8	73	18	8	73	20	0.01	[-0.97; 0.99]	1.1%
Kubo et al. 2006 [113]	9	207	22	9	204	19	0.14	[-0.79; 1.06]	1.2%
Kubo et al. 2006 [113]	9	205	17	9	202	13	0.19	[-0.74; 1.12]	1.2%
Kubo et al. 2006 [96]	9	78	20	9	78	19	-0.02	[-0.95; 0.90]	1.2%
Kubo et al. 2007 [85]	10	58	8	10	59	8	-0.08	[-0.96; 0.79]	1.4%
Kubo et al. 2009 [114]	10	63	8	10	60	7	0.32	[-0.57; 1.20]	1.3%
Kubo et al. 2009 [114]	10	62	8	10	61	7	0.10	[-0.78; 0.98]	1.4%
Kubo et al. 2010 [115]	10	58	8	10	59	8	-0.08	[-0.96; 0.79]	1.4%
Kubo et al. 2010 [115]	10	68	8	10	66	8	0.21	[-0.67; 1.09]	1.3%
Kubo et al. 2010 [88]	8	81	20	8	80	19	0.04	[-0.94; 1.02]	1.1%
Kubo et al. 2012 [89]	9	74	28	9	72	33	0.06	[-0.86; 0.99]	1.2%
Kubo et al. 2017 [116]	9	73	10	9	72	11	0.08	[-0.84; 1.01]	1.2%
Kubo et al. 2017 [116]	9	74	9	9	74	8	0.03	[-0.89; 0.96]	1.2%
Kubo et al. 2017 [86]	11	66	8	11	65	8	0.14	[-0.70; 0.97]	1.5%
Malliaras et al. 2013 [61]	9	118	9	9	112	6	0.73	[-0.23; 1.70]	1.1%
Malliaras et al. 2013 [61]	10	120	19	10	116	8	0.27	[-0.61; 1.15]	1.3%
Malliaras et al. 2013 [61]	10	120	19	10	114	19	0.33	[-0.55; 1.22]	1.3%
Massey et al. 2018 [66]	14	96	8	14	99	10	-0.30	[-1.04; 0.45]	1.9%
Massey et al. 2018 [66]	15	98	13	15	97	13	0.03	[-0.69; 0.75]	2.0%
McMahon et al. 2013 [67]	10	73	14	10	70	13	0.21	[-0.67; 1.09]	1.3%
McMahon et al. 2013 [67]	11	77	19	11	72	14	0.26	[-0.58; 1.10]	1.5%
McMahon et al. 2013 [67]	11	76	16	11	71	13	0.31	[-0.53; 1.16]	1.5%
McMahon et al. 2018 [68]	8	81	13	8	77	13	0.32	[-0.67; 1.30]	1.1%
McMahon et al. 2018 [68]	8	70	22	8	66	11	0.20	[-0.78; 1.18]	1.1%
Mouraux et al. 2000 [118]	10	257	61	10	240	49	0.29	[-0.59; 1.18]	1.3%
Quinlan et al. 2021 [121]	8	90	9	8	90	10	0.08	[-0.90; 1.06]	1.1%
Quinlan et al. 2021 [121]	10	85	5	10	84	6	0.16	[-0.72; 1.04]	1.4%
Quinlan et al. 2021 [121]	9	87	11	9	87	10	-0.03	[-0.95; 0.90]	1.2%
Quinlan et al. 2021 [121]	8	89	12	8	89	11	0.01	[-0.97; 0.99]	1.1%
Reeves et al. 2003 [69]	9	84	22	9	84	24	0.00	[-0.92; 0.93]	1.2%
Sanz-Lopez et al. 2016 [123]	20	82	23	20	64	11	1.04	[0.38; 1.71]	2.4%
Seynnes et al. 2009 [70]	15	107	4	15	103	4	1.01	[0.24; 1.77]	1.8%
Waugh et al. 2014 [74]	10	37	6	10	36	6	0.14	[-0.74; 1.02]	1.4%
Waugh et al. 2018 [54]	14	61	17	14	62	15	-0.08	[-0.82; 0.67]	1.9%
Waugh et al. 2018 [54]	14	60	14	14	58	13	0.14	[-0.60; 0.88]	1.9%
Random effects model	658			659			0.23	[0.12; 0.34]	87.6%
Heterogeneity: $I^2 = 0\%$, p = NA									
**Random effects model**	**751**			**752**			**0.22**	**[0.12; 0.33]**	**100.0%**
Heterogeneity: $I^2 = 0\%$, $p = 1.00$									
Residual heterogeneity: $I^2 = 0\%$, $p = 1.00$									

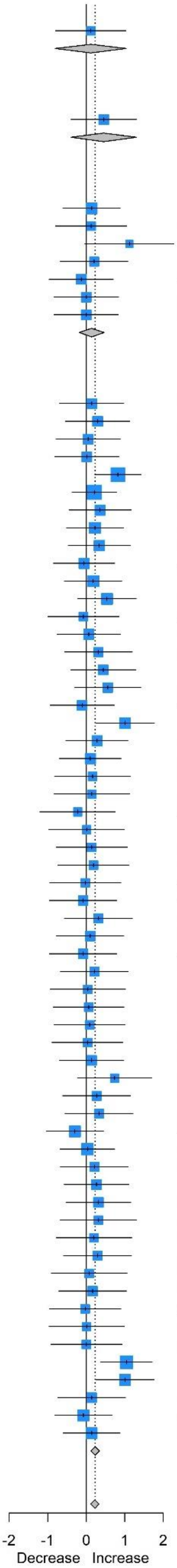

<u>S10.</u> Descriptive statistics for sub-group analyses in Figure 6, showing mean, 95% confidence intervals and range of quantitative grouping variables.

Variable	Sub-group	N	Mean	95%CI	Range
Intensity (%)	Low	7	57.86	55.88 – 59.84	55 – 60
	High	54	82.02	79.54 – 84.50	70 – 120
Strain (%)	Low	2	2.99	2.96 – 3.01	2.97 – 3.00
	High	6	6.12	5.33 – 6.91	4.72 – 6.90
Volume (au)	Low	23	1715.87	1332.73 – 2099.01	280 - 3060
	High	33	5692.73	3936.78 – 7448.68	3200 – 32400
Duration (weeks)	<12	12	7.33	6.42 – 8.25	4 – 10
	≥12	40	13.40	11.44 – 15.36	12 – 52

S11. Forest plot for the meta-analysis of stiffness subdivided by protocol intensity (high versus low) showing standardised mean differences (SMD) and 95% confidence intervals (CI) of resistance training studies.

Study	Post n	Post Mean	Post SD	Pre n	Pre Mean	Pre SD	SMD	[95% CI]	Weight
**High Intensity**									
Albracht et al. 2013 [5]	13	315	53	13	272	48	0.82	[0.02; 1.63]	1.9%
Arampatzis et al. 2007 [19]	11	228	40	11	168	37	1.52	[0.55; 2.49]	1.5%
Arampatzis et al. 2010 [20]	11	302	57	11	258	51	0.79	[-0.09; 1.66]	1.7%
Bohm et al. 2014 [21]	12	457	132	12	370	132	0.64	[-0.18; 1.47]	1.8%
Bohm et al. 2014 [21]	14	539	254	14	336	89	1.04	[0.24; 1.84]	1.9%
Bohm et al. 2014 [21]	12	579	172	12	377	106	1.37	[0.46; 2.27]	1.7%
Bohm et al. 2021 [17]	13	111	59	13	85	36	0.52	[-0.27; 1.30]	1.9%
Carroll et al. 2011 [62]	12	3335	1334	12	2928	1320	0.30	[-0.51; 1.10]	1.9%
Centner et al. 2019 [71]	14	565	158	14	402	103	1.19	[0.38; 2.01]	1.9%
Duclay et al. 2009 [105]	10	291	125	10	249	90	0.38	[-0.51; 1.26]	1.7%
Eriksen et al. 2018 [63]	9	3890	2430	9	2560	1530	0.62	[-0.33; 1.58]	1.6%
Eriksen et al. 2018 [63]	12	1900	693	12	1800	624	0.15	[-0.65; 0.95]	1.9%
Eriksen et al. 2019 [64]	10	4420	1075	10	4060	1360	0.28	[-0.60; 1.16]	1.7%
Geremia et al. 2018 [72]	15	350	59	15	192	36	3.15	[2.03; 4.26]	1.3%
Kay et al. 2016 [110]	13	13	3	13	10	2	1.10	[0.27; 1.94]	1.8%
Kongsgaard et al. 2007 [65]	12	4213	1406	12	3676	1306	0.38	[-0.43; 1.19]	1.9%
Kubo et al. 2001 [51]	8	106	33	8	68	21	1.31	[0.20; 2.42]	1.3%
Kubo et al. 2002 [111]	8	34	10	8	26	9	0.80	[-0.23; 1.83]	1.4%
Kubo et al. 2006 [112]	8	59	24	8	51	22	0.29	[-0.69; 1.28]	1.5%
Kubo et al. 2006 [112]	8	1786	660	8	1790	559	-0.01	[-0.99; 0.97]	1.5%
Kubo et al. 2006 [113]	9	86	36	9	79	21	0.25	[-0.68; 1.17]	1.6%
Kubo et al. 2006 [113]	9	122	40	9	81	26	1.17	[0.15; 2.18]	1.5%
Kubo et al. 2006 [96]	9	59	23	9	46	19	0.62	[-0.33; 1.57]	1.6%
Kubo et al. 2006 [96]	9	1819	710	9	1676	662	0.20	[-0.73; 1.13]	1.6%
Kubo et al. 2007 [85]	10	166	44	10	128	26	1.01	[0.07; 1.96]	1.6%
Kubo et al. 2009 [114]	10	110	36	10	67	19	1.43	[0.43; 2.44]	1.5%
Kubo et al. 2009 [114]	10	96	37	10	72	20	0.75	[-0.16; 1.67]	1.7%
Kubo et al. 2009 [114]	10	1833	892	10	1071	639	0.94	[0.01; 1.88]	1.6%
Kubo et al. 2009 [114]	10	1253	410	10	999	426	0.58	[-0.32; 1.48]	1.7%
Kubo et al. 2010 [115]	10	41	13	10	32	5	0.85	[-0.08; 1.77]	1.6%
Kubo et al. 2010 [115]	10	96	37	10	72	20	0.75	[-0.16; 1.67]	1.7%
Kubo et al. 2010 [88]	8	104	37	8	69	19	1.12	[0.04; 2.20]	1.4%
Kubo et al. 2012 [89]	9	277	111	9	183	45	1.06	[0.05; 2.06]	1.5%
Kubo et al. 2017 [116]	9	1414	355	9	1004	384	1.06	[0.05; 2.06]	1.5%
Kubo et al. 2017 [116]	9	1457	440	9	1289	544	0.32	[-0.61; 1.26]	1.6%
Kubo et al. 2017 [86]	11	32	9	11	24	5	1.17	[0.25; 2.09]	1.6%
Malliaras et al. 2013 [61]	9	2338	638	9	1560	793	1.03	[0.03; 2.03]	1.5%
Malliaras et al. 2013 [61]	10	2536	850	10	1822	898	0.78	[-0.13; 1.70]	1.6%
Malliaras et al. 2013 [61]	10	2508	1066	10	1387	360	1.35	[0.36; 2.34]	1.5%
Massey et al. 2018 [66]	14	595	101	14	592	118	0.03	[-0.71; 0.77]	2.0%
Massey et al. 2018 [66]	15	687	285	15	560	177	0.52	[-0.21; 1.25]	2.1%
Massey et al. 2018 [66]	14	3122	632	14	2605	446	0.92	[0.13; 1.70]	1.9%
Massey et al. 2018 [66]	15	3239	575	15	2835	444	0.77	[0.02; 1.51]	2.0%
McMahon et al. 2013 [67]	10	1221	594	10	916	441	0.56	[-0.34; 1.46]	1.7%
McMahon et al. 2013 [67]	11	1167	353	11	765	242	1.28	[0.34; 2.21]	1.6%
McMahon et al. 2018 [68]	8	1517	390	8	1132	294	1.05	[-0.01; 2.12]	1.4%
McMahon et al. 2018 [68]	8	887	305	8	619	204	0.98	[-0.08; 2.03]	1.4%
Onambélé et al. 2008 [120]	12	40	23	12	26	13	0.72	[-0.11; 1.55]	1.8%
Seynnes et al. 2009 [70]	15	2288	2115	15	1864	1813	0.21	[-0.51; 0.93]	2.1%
Tillin et al. 2012 [125]	10	697	103	10	520	86	1.79	[0.71; 2.86]	1.4%
Waugh et al. 2018 [54]	14	541	49	14	390	35	3.43	[2.21; 4.65]	1.2%
Waugh et al. 2018 [54]	14	523	53	14	359	53	2.99	[1.87; 4.12]	1.3%
Werkhausen et al. 2018 [56]	11	459	147	11	397	146	0.41	[-0.44; 1.25]	1.8%
Random effects model	577			577			0.86	[0.70; 1.02]	87.5%
Heterogeneity: $I^2 = 42\%$, $p < 0.01$									
**Low Intensity**									
Arampatzis et al. 2007 [19]	11	201	41	11	187	38	0.36	[-0.49; 1.20]	1.8%
Arampatzis et al. 2010 [20]	11	261	56	11	276	53	-0.25	[-1.09; 0.59]	1.8%
Kongsgaard et al. 2007 [65]	12	3375	1251	12	3716	1569	-0.23	[-1.04; 0.57]	1.9%
McMahon et al. 2013 [67]	11	1124	471	11	837	379	0.65	[-0.22; 1.51]	1.8%
Quinlan et al. 2021 [121]	10	2377	608	10	1769	462	1.08	[0.13; 2.03]	1.6%
Quinlan et al. 2021 [121]	9	2512	635	9	1782	570	1.15	[0.13; 2.17]	1.5%
Quinlan et al. 2021 [121]	8	2326	769	8	1376	428	1.44	[0.31; 2.58]	1.3%
Quinlan et al. 2021 [121]	8	2314	466	8	1267	260	2.62	[1.19; 4.06]	0.9%
Random effects model	80			80			0.74	[0.16; 1.31]	12.5%
Heterogeneity: $I^2 = 66\%$, $p < 0.01$									
**Random effects model**	**657**			**657**			**0.84**	**[0.68; 1.00]**	**100.0%**
Heterogeneity: $I^2 = 46\%$, $p < 0.01$									
Residual heterogeneity: $I^2 = 46\%$, $p < 0.01$									

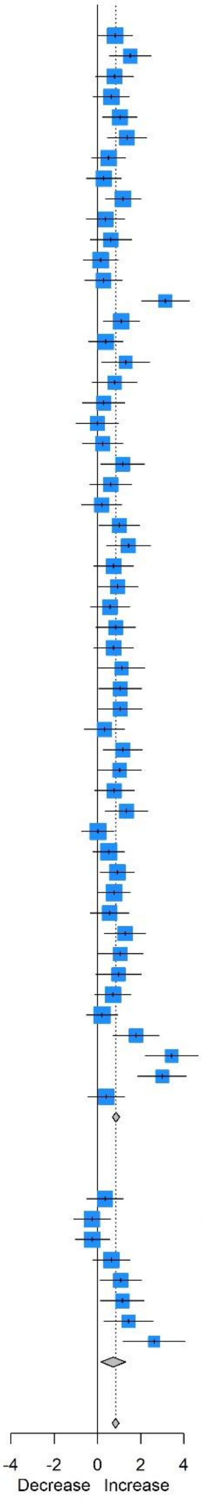

S12. Forest plot for the meta-analysis of modulus subdivided by protocol intensity (high versus low) showing standardised mean differences (SMD) and 95% confidence intervals (CI) of resistance training studies.

Study	Post n	Post Mean	Post SD	Pre n	Pre Mean	Pre SD	SMD	[95% CI]	Weight
High Intensity									
Arampatzis et al. 2007 [19]	11	680	355	11	440	278	0.72	[-0.14; 1.59]	3.2%
Arampatzis et al. 2010 [20]	11	1130	332	11	970	232	0.54	[-0.32; 1.39]	3.3%
Bohm et al. 2014 [21]	12	1050	277	12	890	277	0.56	[-0.26; 1.38]	3.4%
Bohm et al. 2014 [21]	14	1430	636	14	910	262	1.04	[0.24; 1.83]	3.4%
Bohm et al. 2014 [21]	12	1410	381	12	970	277	1.28	[0.38; 2.17]	3.2%
Carroll et al. 2011 [62]	12	1220	450	12	1030	416	0.42	[-0.39; 1.23]	3.4%
Centner et al. 2019 [71]	14	1848	481	14	1540	492	0.61	[-0.15; 1.38]	3.5%
Eriksen et al. 2018 [63]	9	1330	690	9	1230	390	0.17	[-0.76; 1.10]	3.1%
Eriksen et al. 2018 [63]	12	735	263	12	703	253	0.12	[-0.68; 0.92]	3.4%
Eriksen et al. 2019 [64]	10	1560	443	10	1510	474	0.10	[-0.77; 0.98]	3.2%
Geremia et al. 2018 [72]	15	1292	436	15	695	160	1.77	[0.91; 2.63]	3.2%
Kongsgaard et al. 2007 [65]	12	1650	554	12	1470	589	0.30	[-0.50; 1.11]	3.4%
Kubo et al. 2001 [52]	8	433	35	8	288	26	4.45	[2.42; 6.47]	1.2%
Malliaras et al. 2013 [61]	9	942	279	9	620	223	1.22	[0.19; 2.24]	2.8%
Malliaras et al. 2013 [61]	10	1022	339	10	737	390	0.75	[-0.17; 1.66]	3.1%
Malliaras et al. 2013 [61]	10	1011	436	10	570	191	1.25	[0.28; 2.23]	2.9%
Massey et al. 2018 [66]	14	1490	270	14	1230	180	1.10	[0.30; 1.90]	3.4%
Massey et al. 2018 [66]	15	1510	360	15	1320	270	0.58	[-0.15; 1.31]	3.6%
McMahon et al. 2013 [67]	10	1100	120	10	830	90	2.44	[1.22; 3.65]	2.4%
McMahon et al. 2013 [67]	11	1150	110	11	780	100	3.39	[2.00; 4.77]	2.1%
McMahon et al. 2018 [68]	8	990	255	8	780	170	0.92	[-0.13; 1.96]	2.8%
McMahon et al. 2018 [68]	8	600	226	8	420	113	0.95	[-0.10; 2.00]	2.8%
Seynnes et al. 2009 [70]	15	1160	1201	15	980	1162	0.15	[-0.57; 0.87]	3.6%
Waugh et al. 2018 [54]	14	1892	803	14	1261	459	0.94	[0.15; 1.72]	3.4%
Waugh et al. 2018 [54]	14	1720	535	14	1242	420	0.96	[0.17; 1.75]	3.4%
Random effects model	290			290			0.91	[0.63; 1.18]	77.1%
Heterogeneity: $I^2 = 57\%$, $p < 0.01$									
Low Intensity									
Arampatzis et al. 2007 [19]	11	400	293	11	420	401	-0.05	[-0.89; 0.78]	3.3%
Arampatzis et al. 2010 [20]	11	970	265	11	1010	199	-0.16	[-1.00; 0.67]	3.3%
Kongsgaard et al. 2007 [65]	12	1360	658	12	1420	727	-0.08	[-0.88; 0.72]	3.4%
McMahon et al. 2013 [67]	11	990	110	11	740	90	2.39	[1.25; 3.54]	2.5%
Quinlan et al. 2021 [121]	10	1510	440	10	1160	290	0.90	[-0.03; 1.83]	3.1%
Quinlan et al. 2021 [121]	9	1460	350	9	1050	270	1.25	[0.22; 2.28]	2.8%
Quinlan et al. 2021 [121]	8	1430	570	8	800	250	1.35	[0.23; 2.47]	2.6%
Quinlan et al. 2021 [121]	8	1280	230	8	710	130	2.88	[1.37; 4.40]	1.9%
Random effects model	80			80			0.95	[0.23; 1.68]	22.9%
Heterogeneity: $I^2 = 77\%$, $p < 0.01$									
**Random effects model**	**370**			**370**			**0.91**	**[0.65; 1.17]**	**100.0%**
Heterogeneity: $I^2 = 63\%$, $p < 0.01$									
Residual heterogeneity: $I^2 = 64\%$, $p < 0.01$									

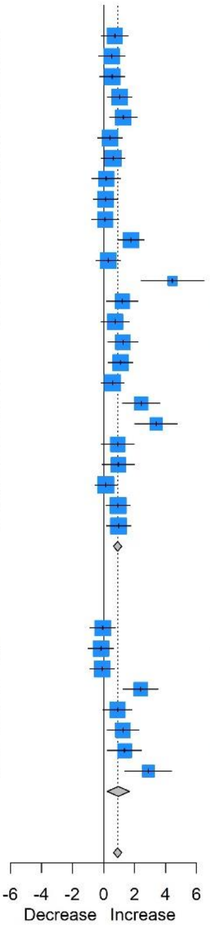

S13. Forest plot for the meta-analysis of cross-sectional area subdivided by protocol intensity (high versus low) showing standardised mean differences (SMD) and 95% confidence intervals (CI) of resistance training studies.

Study	Post n	Post Mean	Post SD	Pre n	Pre Mean	Pre SD	SMD	[95% CI]	Weight
**High Intensity**									
Arampatzis et al. 2007 [19]	11	53	14	11	48	13	0.30	[-0.55; 1.14]	2.0%
Arampatzis et al. 2010 [20]	11	56	36	11	56	38	0.02	[-0.82; 0.85]	2.1%
Bohm et al. 2014 [21]	12	82	12	12	78	11	0.36	[-0.45; 1.16]	2.2%
Bohm et al. 2014 [21]	14	83	14	14	80	13	0.23	[-0.52; 0.97]	2.6%
Bohm et al. 2014 [21]	12	79	10	12	75	9	0.34	[-0.47; 1.14]	2.2%
Carroll et al. 2011 [62]	12	115	33	12	117	35	-0.06	[-0.86; 0.74]	2.2%
Centner et al. 2019 [71]	14	74	17	14	70	18	0.18	[-0.56; 0.92]	2.6%
Eriksen et al. 2018 [63]	9	129	24	9	131	27	-0.07	[-1.00; 0.85]	1.7%
Eriksen et al. 2018 [63]	11	123	33	12	121	28	0.06	[-0.76; 0.88]	2.1%
Eriksen et al. 2019 [64]	10	124	22	10	117	21	0.31	[-0.57; 1.20]	1.8%
Geremia et al. 2018 [72]	15	72	11	15	62	8	1.01	[0.24; 1.77]	2.4%
Kongsgaard et al. 2007 [65]	12	121	14	12	117	14	0.28	[-0.53; 1.08]	2.2%
Kubo et al. 2001 [51]	8	213	19	8	210	16	0.16	[-0.82; 1.14]	1.5%
Kubo et al. 2001 [51]	8	215	21	8	212	18	0.15	[-0.84; 1.13]	1.5%
Kubo et al. 2002 [111]	8	59	8	8	61	9	-0.22	[-1.21; 0.76]	1.5%
Kubo et al. 2006 [112]	8	73	18	8	73	20	0.01	[-0.97; 0.99]	1.5%
Kubo et al. 2006 [113]	9	207	22	9	204	19	0.14	[-0.79; 1.06]	1.7%
Kubo et al. 2006 [113]	9	205	17	9	202	13	0.19	[-0.74; 1.12]	1.7%
Kubo et al. 2006 [96]	9	78	20	9	78	19	-0.02	[-0.95; 0.90]	1.7%
Kubo et al. 2007 [85]	10	58	8	10	59	8	-0.08	[-0.96; 0.79]	1.9%
Kubo et al. 2009 [114]	10	63	8	10	60	7	0.32	[-0.57; 1.20]	1.8%
Kubo et al. 2009 [114]	10	62	8	10	61	7	0.10	[-0.78; 0.98]	1.9%
Kubo et al. 2010 [115]	10	58	8	10	59	8	-0.08	[-0.96; 0.79]	1.9%
Kubo et al. 2010 [115]	10	68	8	10	66	8	0.21	[-0.67; 1.09]	1.9%
Kubo et al. 2010 [88]	8	81	20	8	80	19	0.04	[-0.94; 1.02]	1.5%
Kubo et al. 2012 [89]	9	74	28	9	72	33	0.06	[-0.86; 0.99]	1.7%
Kubo et al. 2017 [116]	9	73	10	9	72	11	0.08	[-0.84; 1.01]	1.7%
Kubo et al. 2017 [116]	9	74	9	9	74	8	0.03	[-0.89; 0.96]	1.7%
Kubo et al. 2017 [86]	11	66	8	11	65	8	0.14	[-0.70; 0.97]	2.0%
Malliaras et al. 2013 [61]	9	118	9	9	112	6	0.73	[-0.23; 1.70]	1.5%
Malliaras et al. 2013 [61]	10	120	19	10	116	8	0.27	[-0.61; 1.15]	1.8%
Malliaras et al. 2013 [61]	10	120	19	10	114	19	0.33	[-0.55; 1.22]	1.8%
Massey et al. 2018 [66]	14	96	8	14	99	10	-0.30	[-1.04; 0.45]	2.6%
Massey et al. 2018 [66]	15	98	13	15	97	13	0.03	[-0.69; 0.75]	2.8%
McMahon et al. 2013 [67]	10	73	14	10	70	13	0.21	[-0.67; 1.09]	1.9%
McMahon et al. 2013 [67]	11	76	16	11	71	13	0.31	[-0.53; 1.16]	2.0%
McMahon et al. 2018 [68]	8	81	13	8	77	13	0.32	[-0.67; 1.30]	1.5%
McMahon et al. 2018 [68]	8	70	22	8	66	11	0.20	[-0.78; 1.18]	1.5%
Mouraux et al. 2000 [118]	10	257	61	10	240	49	0.29	[-0.59; 1.18]	1.8%
Sanz-Lopez et al. 2016 [123]	20	82	23	20	64	11	1.04	[0.38; 1.71]	3.2%
Seynnes et al. 2009 [70]	15	107	4	15	103	4	1.01	[0.24; 1.77]	2.4%
Waugh et al. 2018 [54]	14	61	17	14	62	15	-0.08	[-0.82; 0.67]	2.6%
Waugh et al. 2018 [54]	14	60	14	14	58	13	0.14	[-0.60; 0.88]	2.6%
Random effects model	466			467			0.22	[0.09; 0.35]	85.1%
Heterogeneity: $I^2 = 0\%$, $p = 1.00$									
**Low Intensity**									
Arampatzis et al. 2007 [19]	11	53	15	11	50	15	0.14	[-0.70; 0.98]	2.0%
Arampatzis et al. 2010 [20]	11	60	35	11	58	34	0.05	[-0.79; 0.88]	2.0%
Kongsgaard et al. 2007 [65]	12	118	13	12	116	18	0.10	[-0.70; 0.91]	2.2%
McMahon et al. 2013 [67]	11	77	19	11	72	14	0.26	[-0.58; 1.10]	2.0%
Quinlan et al. 2021 [121]	10	85	5	10	84	6	0.16	[-0.72; 1.04]	1.9%
Quinlan et al. 2021 [121]	9	87	11	9	87	10	-0.03	[-0.95; 0.90]	1.7%
Quinlan et al. 2021 [121]	8	89	12	8	89	11	0.01	[-0.97; 0.99]	1.5%
Quinlan et al. 2021 [121]	8	90	9	8	90	10	0.08	[-0.90; 1.06]	1.5%
Random effects model	80			80			0.10	[-0.21; 0.41]	14.9%
Heterogeneity: $I^2 = 0\%$, $p = 0.99$									
**Random effects model**	**546**			**547**			**0.20**	**[0.08; 0.32]**	**100.0%**
Heterogeneity: $I^2 = 0\%$, $p = 1.00$									
Residual heterogeneity: $I^2 = 0\%$, $p = 1.00$									

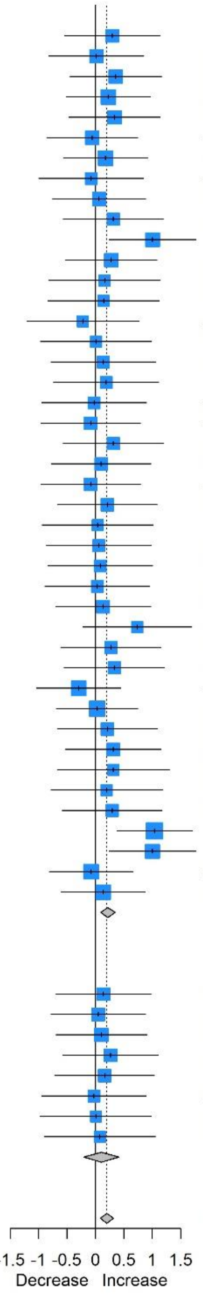

S14. Forest plot for the meta-analysis of stiffness subdivided by protocol strain (high versus low) showing standardised mean differences (SMD) and 95% confidence intervals (CI) of resistance training studies.

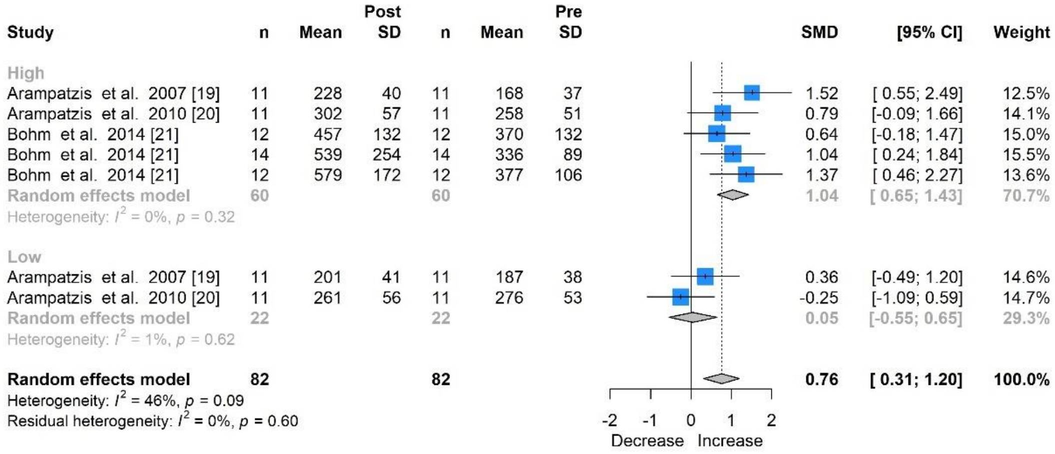

S15. Forest plot for the meta-analysis of modulus subdivided by protocol strain (high versus low) showing standardised mean differences (SMD) and 95% confidence intervals (CI) of resistance training studies.

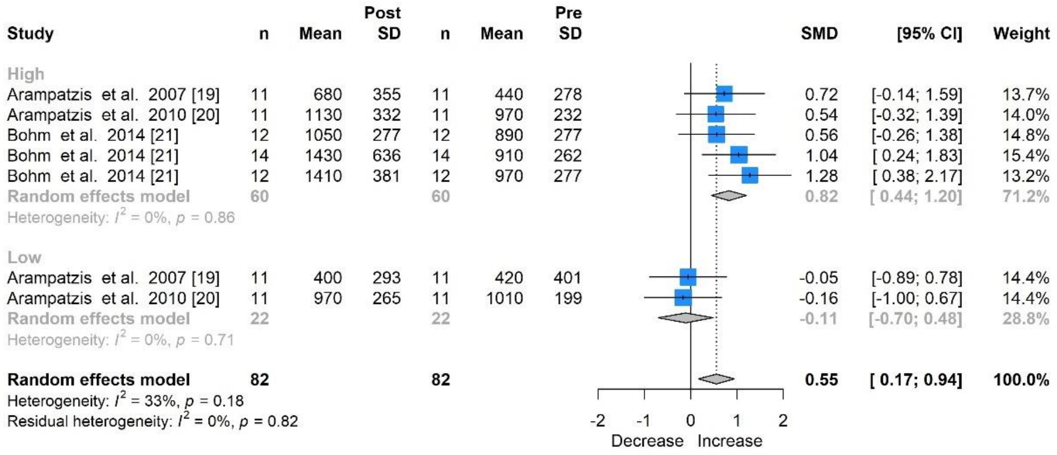

S16. Forest plot for the meta-analysis of cross-sectional area subdivided by protocol strain (high versus low) showing standardised mean differences (SMD) and 95% confidence intervals (CI) of resistance training studies.

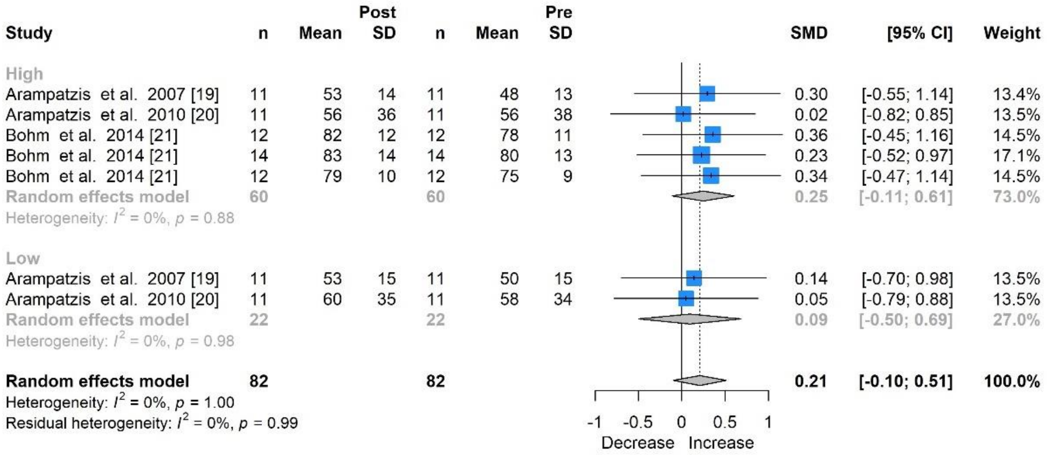

S17. Forest plot for the meta-analysis of stiffness subdivided by training volume (high versus low) showing standardised mean differences (SMD) and 95% confidence intervals (CI) of resistance training studies.

Study	Post n	Post Mean	Post SD	Pre n	Pre Mean	Pre SD	SMD	[95% CI]	Weight
**High volume**									
Arampatzis et al. 2010 [20]	11	261	56	11	276	53	-0.25	[-1.09; 0.59]	2.0%
Arampatzis et al. 2010 [20]	11	302	57	11	258	51	0.79	[-0.09; 1.66]	1.9%
Bohm et al. 2014 [21]	14	539	254	14	336	89	1.04	[0.24; 1.84]	2.1%
Duclay et al. 2009 [105]	10	291	125	10	249	90	0.38	[-0.51; 1.26]	1.9%
Geremia et al. 2018 [72]	15	350	59	15	192	36	3.15	[2.03; 4.26]	1.3%
Kongsgaard et al. 2007 [65]	12	4213	1406	12	3676	1306	0.38	[-0.43; 1.19]	2.1%
Kubo et al. 2006 [113]	9	86	36	9	79	21	0.25	[-0.68; 1.17]	1.7%
Kubo et al. 2006 [113]	9	122	40	9	81	26	1.17	[0.15; 2.18]	1.5%
Kubo et al. 2006 [96]	9	59	23	9	46	19	0.62	[-0.33; 1.57]	1.7%
Kubo et al. 2006 [96]	9	1819	710	9	1676	662	0.20	[-0.73; 1.13]	1.7%
Kubo et al. 2009 [114]	10	96	37	10	72	20	0.75	[-0.16; 1.67]	1.8%
Kubo et al. 2009 [114]	10	1253	410	10	999	426	0.58	[-0.32; 1.48]	1.8%
Kubo et al. 2010 [115]	10	41	13	10	32	5	0.85	[-0.08; 1.77]	1.8%
Kubo et al. 2010 [115]	10	96	37	10	72	20	0.75	[-0.16; 1.67]	1.8%
Kubo et al. 2017 [116]	9	1414	355	9	1004	384	1.06	[0.05; 2.06]	1.6%
Kubo et al. 2017 [116]	9	1457	440	9	1289	544	0.32	[-0.61; 1.26]	1.7%
Malliaras et al. 2013 [61]	10	2536	850	10	1822	898	0.78	[-0.13; 1.70]	1.8%
Massey et al. 2018 [66]	14	595	101	14	592	118	0.03	[-0.71; 0.77]	2.3%
Massey et al. 2018 [66]	14	3122	632	14	2605	446	0.92	[0.13; 1.70]	2.2%
McMahon et al. 2013 [67]	10	1221	594	10	916	441	0.56	[-0.34; 1.46]	1.8%
McMahon et al. 2013 [67]	11	1124	471	11	837	379	0.65	[-0.22; 1.51]	1.9%
McMahon et al. 2013 [67]	11	1167	353	11	765	242	1.28	[0.34; 2.21]	1.7%
Onambélé et al. 2008 [120]	12	40	23	12	26	13	0.72	[-0.11; 1.55]	2.0%
Quinlan et al. 2021 [121]	10	2377	608	10	1769	462	1.08	[0.13; 2.03]	1.7%
Quinlan et al. 2021 [121]	9	2512	635	9	1782	570	1.15	[0.13; 2.17]	1.5%
Quinlan et al. 2021 [121]	8	2326	769	8	1376	428	1.44	[0.31; 2.58]	1.3%
Quinlan et al. 2021 [121]	8	2314	466	8	1267	260	2.62	[1.19; 4.06]	0.9%
Seynnes et al. 2009 [70]	15	2288	2115	15	1864	1813	0.21	[-0.51; 0.93]	2.4%
Tillin et al. 2012 [125]	10	697	103	10	520	86	1.79	[0.71; 2.86]	1.4%
Waugh et al. 2018 [54]	14	456	49	14	390	35	1.51	[0.66; 2.37]	1.9%
Waugh et al. 2018 [54]	14	461	40	14	359	53	2.11	[1.16; 3.06]	1.7%
Werkhausen et al. 2018 [56]	11	459	147	11	397	146	0.41	[-0.44; 1.25]	2.0%
Random effects model	348			348			0.85	[0.62; 1.07]	57.0%
Heterogeneity: $I^2 = 49\%$, $p = 0.65$									
**Low volume**									
Albracht et al. 2013[5]	13	315	53	13	272	48	0.82	[0.02; 1.63]	2.1%
Arampatzis et al. 2007 [19]	11	201	41	11	187	38	0.36	[-0.49; 1.20]	2.0%
Arampatzis et al. 2007 [19]	11	228	40	11	168	37	1.52	[0.55; 2.49]	1.6%
Bohm et al. 2014 [21]	12	457	132	12	370	132	0.64	[-0.18; 1.47]	2.0%
Bohm et al. 2014 [21]	12	579	172	12	377	106	1.37	[0.46; 2.27]	1.8%
Bohm et al. 2021 [17]	13	111	59	13	85	36	0.52	[-0.27; 1.30]	2.2%
Carroll et al. 2011 [62]	12	3335	1334	12	2928	1320	0.30	[-0.51; 1.10]	2.1%
Centner et al. 2019 [71]	14	565	158	14	402	103	1.19	[0.38; 2.01]	2.1%
Eriksen et al. 2018 [63]	9	3890	2430	9	2560	1530	0.62	[-0.33; 1.58]	1.7%
Eriksen et al. 2018 [63]	12	1900	693	12	1800	624	0.15	[-0.65; 0.95]	2.1%
Kubo et al. 2001 [51]	8	106	33	8	68	21	1.31	[0.20; 2.42]	1.3%
Kubo et al. 2006 [112]	8	59	24	8	51	22	0.29	[-0.69; 1.28]	1.6%
Kubo et al. 2006 [112]	8	1786	660	8	1790	559	-0.01	[-0.99; 0.97]	1.6%
Kubo et al. 2009 [114]	10	110	36	10	67	19	1.43	[0.43; 2.44]	1.6%
Kubo et al. 2009 [114]	10	1833	892	10	1071	639	0.94	[0.01; 1.88]	1.7%
Kubo et al. 2010 [88]	8	104	37	8	69	19	1.12	[0.04; 2.20]	1.4%
Kubo et al. 2012 [89]	9	277	111	9	183	45	1.06	[0.05; 2.06]	1.6%
Kubo et al. 2017 [86]	11	32	9	11	24	5	1.17	[0.25; 2.09]	1.8%
Malliaras et al. 2013 [61]	9	2338	638	9	1560	793	1.03	[0.03; 2.03]	1.6%
Malliaras et al. 2013 [61]	10	2508	1066	10	1387	360	1.35	[0.36; 2.34]	1.6%
Massey et al. 2018 [66]	15	687	285	15	560	177	0.52	[-0.21; 1.25]	2.4%
Massey et al. 2018 [66]	15	3239	575	15	2835	444	0.77	[0.02; 1.51]	2.3%
McMahon et al. 2018 [68]	8	1517	390	8	1132	294	1.05	[-0.01; 2.12]	1.4%
McMahon et al. 2018 [68]	8	887	305	8	619	204	0.98	[-0.08; 2.03]	1.5%
Random effects model	256			256			0.81	[0.62; 0.99]	43.0%
Heterogeneity: $I^2 = 0\%$, $p < 0.01$									
**Random effects model**	**604**			**604**			**0.83**	**[0.68; 0.97]**	**100.0%**
Heterogeneity: $I^2 = 32\%$, $p = 0.01$									
Residual heterogeneity: $I^2 = 33\%$, $p = 0.01$									

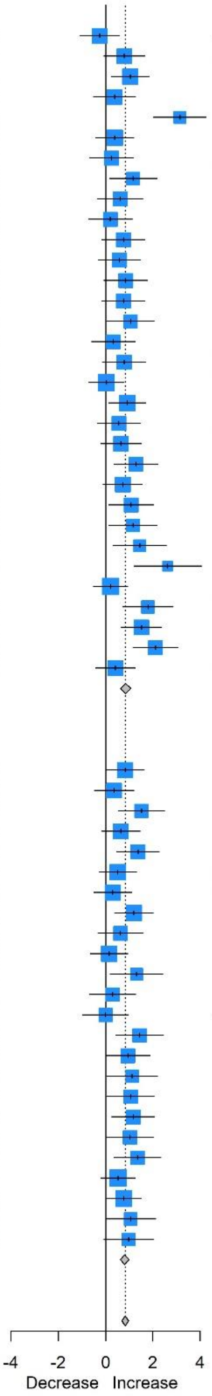

S18. Forest plot for the meta-analysis of modulus subdivided by training volume (high versus low) showing standardised mean differences (SMD) and 95% confidence intervals (CI) of resistance training studies.

Study	Post n	Post Mean	Post SD	Pre n	Pre Mean	Pre SD	SMD	[95% CI]	Weight
**High volume**									
Arampatzis et al. 2010 [20]	11	970	265	11	1010	199	-0.16	[-1.00; 0.67]	3.7%
Arampatzis et al. 2010 [20]	11	1130	332	11	970	232	0.54	[-0.32; 1.39]	3.6%
Bohm et al. 2014 [21]	14	1430	636	14	910	262	1.04	[0.24; 1.83]	3.8%
Geremia et al. 2018 [72]	15	1292	436	15	695	160	1.77	[0.91; 2.63]	3.6%
Kongsgaard et al. 2007 [65]	12	1650	554	12	1470	589	0.30	[-0.50; 1.11]	3.8%
Malliaras et al. 2013 [61]	10	1022	339	10	737	390	0.75	[-0.17; 1.66]	3.4%
Massey et al. 2018 [66]	14	1490	270	14	1230	180	1.10	[0.30; 1.90]	3.8%
McMahon et al. 2013 [67]	10	1100	120	10	830	90	2.44	[1.22; 3.65]	2.7%
McMahon et al. 2013 [67]	11	990	110	11	740	90	2.39	[1.25; 3.54]	2.8%
McMahon et al. 2013 [67]	11	1150	110	11	780	100	3.39	[2.00; 4.77]	2.3%
Quinlan et al. 2021 [121]	10	1510	440	10	1160	290	0.90	[-0.03; 1.83]	3.4%
Quinlan et al. 2021 [121]	9	1460	350	9	1050	270	1.25	[0.22; 2.28]	3.1%
Quinlan et al. 2021 [121]	8	1430	570	8	800	250	1.35	[0.23; 2.47]	2.9%
Quinlan et al. 2021 [121]	8	1280	230	8	710	130	2.88	[1.37; 4.40]	2.1%
Seynnes et al. 2009 [70]	15	1160	1201	15	980	1162	0.15	[-0.57; 0.87]	4.0%
Waugh et al. 2018 [54]	14	1586	611	14	1261	459	0.58	[-0.18; 1.34]	3.9%
Waugh et al. 2018 [54]	14	1529	459	14	1242	420	0.63	[-0.13; 1.39]	3.9%
Random effects model	197			197			1.13	[0.73; 1.53]	56.6%
Heterogeneity: $I^2 = 69\%$, $p = 0.02$									
**Low volume**									
Arampatzis et al. 2007 [19]	11	400	293	11	420	401	-0.05	[-0.89; 0.78]	3.7%
Arampatzis et al. 2007 [19]	11	680	355	11	440	278	0.72	[-0.14; 1.59]	3.6%
Bohm et al. 2014 [21]	12	1050	277	12	890	277	0.56	[-0.26; 1.38]	3.7%
Bohm et al. 2014 [21]	12	1410	381	12	970	277	1.28	[0.38; 2.17]	3.5%
Centner et al. 2019 [71]	14	1848	481	14	1540	492	0.61	[-0.15; 1.38]	3.9%
Eriksen et al. 2018 [63]	9	1330	690	9	1230	390	0.17	[-0.76; 1.10]	3.4%
Eriksen et al. 2018 [63]	12	735	263	12	703	253	0.12	[-0.68; 0.92]	3.8%
Kubo et al. 2001 [52]	8	433	35	8	288	26	4.45	[2.42; 6.47]	1.4%
Malliaras et al. 2013 [61]	9	942	279	9	620	223	1.22	[0.19; 2.24]	3.1%
Malliaras et al. 2013 [61]	10	1011	436	10	570	191	1.25	[0.28; 2.23]	3.3%
Massey et al. 2018 [66]	15	1510	360	15	1320	270	0.58	[-0.15; 1.31]	4.0%
McMahon et al. 2018 [68]	8	990	255	8	780	170	0.92	[-0.13; 1.96]	3.1%
McMahon et al. 2018 [68]	8	600	226	8	420	113	0.95	[-0.10; 2.00]	3.1%
Random effects model	139			139			0.77	[0.40; 1.13]	43.4%
Heterogeneity: $I^2 = 50\%$, $p < 0.01$									
**Random effects model**	**336**			**336**			**0.97**	**[0.70; 1.25]**	**100.0%**
Heterogeneity: $I^2 = 63\%$, $p < 0.01$									
Residual heterogeneity: $I^2 = 63\%$, $p < 0.01$									

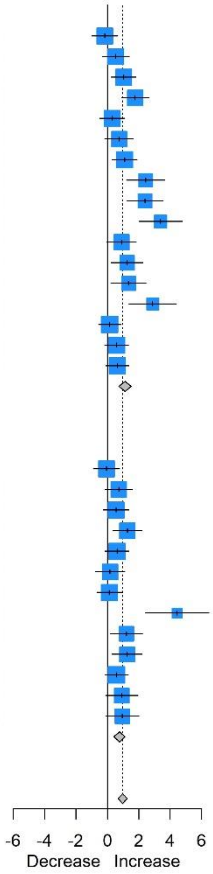

S19. Forest plot for the meta-analysis of cross-sectional area subdivided by training volume (high versus low) showing standardised mean differences (SMD) and 95% confidence intervals (CI) of resistance training studies.

Study	Post n	Post Mean	Post SD	Pre n	Pre Mean	Pre SD	SMD	[95% CI]	Weight
**High volume**									
Arampatzis et al. 2010 [20]	11	60	35	11	58	34	0.05	[-0.79; 0.88]	2.1%
Arampatzis et al. 2010 [20]	11	56	36	11	56	38	0.02	[-0.82; 0.85]	2.1%
Bohm et al. 2014 [21]	14	83	14	14	80	13	0.23	[-0.52; 0.97]	2.7%
Geremia et al. 2018 [72]	15	72	11	15	62	8	1.01	[0.24; 1.77]	2.5%
Kongsgaard et al. 2007 [65]	12	121	14	12	117	14	0.28	[-0.53; 1.08]	2.3%
Kubo et al. 2001 [51]	8	213	19	8	210	16	0.16	[-0.82; 1.14]	1.5%
Kubo et al. 2002 [111]	8	59	8	8	61	9	-0.22	[-1.21; 0.76]	1.5%
Kubo et al. 2006 [113]	9	207	22	9	204	19	0.14	[-0.79; 1.06]	1.7%
Kubo et al. 2006 [113]	9	205	17	9	202	13	0.19	[-0.74; 1.12]	1.7%
Kubo et al. 2006 [96]	9	78	20	9	78	19	-0.02	[-0.95; 0.90]	1.7%
Kubo et al. 2007 [85]	10	58	8	10	59	8	-0.08	[-0.96; 0.79]	1.9%
Kubo et al. 2009 [114]	10	62	8	10	61	7	0.10	[-0.78; 0.98]	1.9%
Kubo et al. 2010 [115]	10	58	8	10	59	8	-0.08	[-0.96; 0.79]	1.9%
Kubo et al. 2010 [115]	10	68	8	10	66	8	0.21	[-0.67; 1.09]	1.9%
Kubo et al. 2017 [116]	9	73	10	9	72	11	0.08	[-0.84; 1.01]	1.7%
Kubo et al. 2017 [116]	9	74	9	9	74	8	0.03	[-0.89; 0.96]	1.7%
Malliaras et al. 2013 [61]	10	120	19	10	116	8	0.27	[-0.61; 1.15]	1.9%
Massey et al. 2018 [66]	14	96	8	14	99	10	-0.30	[-1.04; 0.45]	2.7%
McMahon et al. 2013 [67]	10	73	14	10	70	13	0.21	[-0.67; 1.09]	1.9%
McMahon et al. 2013 [67]	11	77	19	11	72	14	0.26	[-0.58; 1.10]	2.1%
McMahon et al. 2013 [67]	11	76	16	11	71	13	0.31	[-0.53; 1.16]	2.1%
Mouraux et al. 2000 [118]	10	257	61	10	240	49	0.29	[-0.59; 1.18]	1.9%
Quinlan et al. 2021 [121]	10	85	5	10	84	6	0.16	[-0.72; 1.04]	1.9%
Quinlan et al. 2021 [121]	9	87	11	9	87	10	-0.03	[-0.95; 0.90]	1.7%
Quinlan et al. 2021 [121]	8	89	12	8	89	11	0.01	[-0.97; 0.99]	1.6%
Quinlan et al. 2021 [121]	8	90	9	8	90	10	0.08	[-0.90; 1.06]	1.6%
Seynnes et al. 2009 [70]	15	107	4	15	103	4	1.01	[0.24; 1.77]	2.5%
Waugh et al. 2018 [54]	14	61	17	14	62	15	-0.08	[-0.82; 0.67]	2.7%
Waugh et al. 2018 [54]	14	60	14	14	58	13	0.14	[-0.60; 0.88]	2.7%
Random effects model	308			308			0.17	[0.01; 0.33]	58.8%
Heterogeneity: $I^2 = 0\%$, $p = 0.97$									
**Low volume**									
Arampatzis et al. 2007 [19]	11	53	15	11	50	15	0.14	[-0.70; 0.98]	2.1%
Arampatzis et al. 2007 [19]	11	53	14	11	48	13	0.30	[-0.55; 1.14]	2.1%
Bohm et al. 2014 [21]	12	82	12	12	78	11	0.36	[-0.45; 1.16]	2.3%
Bohm et al. 2014 [21]	12	79	10	12	75	9	0.34	[-0.47; 1.14]	2.3%
Carroll et al. 2011 [62]	12	115	33	12	117	35	-0.06	[-0.86; 0.74]	2.3%
Centner et al. 2019 [71]	14	74	17	14	70	18	0.18	[-0.56; 0.92]	2.7%
Eriksen et al. 2018 [63]	9	129	24	9	131	27	-0.07	[-1.00; 0.85]	1.7%
Eriksen et al. 2018 [63]	11	123	33	12	121	28	0.06	[-0.76; 0.88]	2.2%
Kubo et al. 2001 [51]	8	215	21	8	212	18	0.15	[-0.84; 1.13]	1.5%
Kubo et al. 2006 [112]	8	73	18	8	73	20	0.01	[-0.97; 0.99]	1.6%
Kubo et al. 2009 [114]	10	63	8	10	60	7	0.32	[-0.57; 1.20]	1.9%
Kubo et al. 2010 [88]	8	81	20	8	80	19	0.04	[-0.94; 1.02]	1.6%
Kubo et al. 2012 [89]	9	74	28	9	72	33	0.06	[-0.86; 0.99]	1.7%
Kubo et al. 2017 [86]	11	66	8	11	65	8	0.14	[-0.70; 0.97]	2.1%
Malliaras et al. 2013 [61]	9	118	9	9	112	6	0.73	[-0.23; 1.70]	1.6%
Malliaras et al. 2013 [61]	10	120	19	10	114	19	0.33	[-0.55; 1.22]	1.9%
Massey et al. 2018 [66]	15	98	13	15	97	13	0.03	[-0.69; 0.75]	2.9%
McMahon et al. 2018 [68]	8	81	13	8	77	13	0.32	[-0.67; 1.30]	1.5%
McMahon et al. 2018 [68]	8	70	22	8	66	11	0.20	[-0.78; 1.18]	1.5%
Sanz-Lopez et al. 2016 [123]	20	82	23	20	64	11	1.04	[0.38; 1.71]	3.4%
Random effects model	216			217			0.25	[0.06; 0.44]	41.2%
Heterogeneity: $I^2 = 0\%$, $p = 0.99$									
**Random effects model**	**524**			**525**			**0.20**	**[0.08; 0.33]**	**100.0%**
Heterogeneity: $I^2 = 0\%$, $p = 1.00$									
Residual heterogeneity: $I^2 = 0\%$, $p = 1.00$									

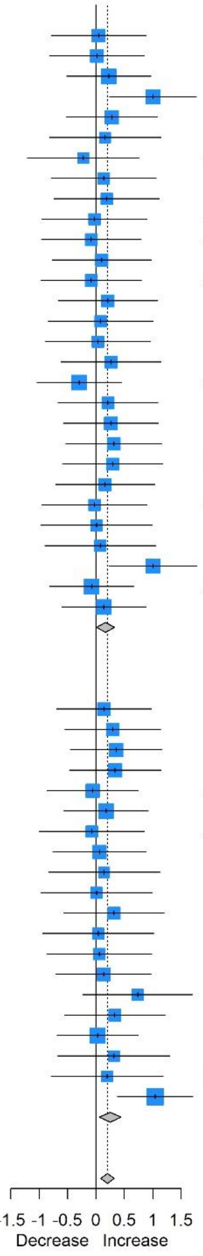

S20. Forest plot for the meta-analysis of stiffness subdivided by protocol duration showing standardised mean differences (SMD) and 95% confidence intervals (CI) of high intensity, resistance training studies.

Study	Post n	Post Mean	Post SD	Pre n	Pre Mean	Pre SD	SMD	[95% CI]	Weight
**<12 weeks**									
Duclay et al. 2009 [105]	10	291	125	10	249	90	0.38	[-0.51; 1.26]	2.0%
Kay et al. 2016 [110]	13	13	3	13	10	2	1.10	[0.27; 1.94]	2.1%
Kubo et al. 2002 [111]	8	34	10	8	26	9	0.80	[-0.23; 1.83]	1.6%
McMahon et al. 2013 [67]	10	1221	594	10	916	441	0.56	[-0.34; 1.46]	1.9%
McMahon et al. 2013 [67]	11	1167	353	11	765	242	1.28	[0.34; 2.21]	1.8%
McMahon et al. 2018 [68]	8	1517	390	8	1132	294	1.05	[-0.01; 2.12]	1.6%
McMahon et al. 2018 [68]	8	887	305	8	619	204	0.98	[-0.08; 2.03]	1.6%
Seynnes et al. 2009 [70]	15	2288	2115	15	1864	1813	0.21	[-0.51; 0.93]	2.4%
Tillin et al. 2012 [125]	10	697	103	10	520	86	1.79	[0.71; 2.86]	1.5%
Werkhausen et al. 2018 [56]	11	459	147	11	397	146	0.41	[-0.44; 1.25]	2.1%
Random effects model	104			104			0.78	[0.48; 1.08]	18.6%
Heterogeneity: I^2 = 7%, p < 0.01									
**12 weeks+**									
Albracht et al. 2013 [5]	13	315	53	13	272	48	0.82	[0.02; 1.63]	2.2%
Arampatzis et al. 2007 [19]	11	228	40	11	168	37	1.52	[0.55; 2.49]	1.8%
Arampatzis et al. 2010 [20]	11	302	57	11	258	51	0.79	[-0.09; 1.66]	2.0%
Bohm et al. 2014 [21]	12	457	132	12	370	132	0.64	[-0.18; 1.47]	2.1%
Bohm et al. 2014 [21]	14	539	254	14	336	89	1.04	[0.24; 1.84]	2.2%
Bohm et al. 2014 [21]	12	579	172	12	377	106	1.37	[0.46; 2.27]	1.9%
Bohm et al. 2021 [17]	13	111	59	13	85	36	0.52	[-0.27; 1.30]	2.2%
Carroll et al. 2011 [62]	12	3335	1334	12	2928	1320	0.30	[-0.51; 1.10]	2.2%
Centner et al. 2019 [71]	14	565	158	14	402	103	1.19	[0.38; 2.01]	2.1%
Eriksen et al. 2018 [63]	9	3890	2430	9	2560	1530	0.62	[-0.33; 1.58]	1.8%
Eriksen et al. 2018 [63]	12	1900	693	12	1800	624	0.15	[-0.65; 0.95]	2.2%
Eriksen et al. 2019 [64]	10	4420	1075	10	4060	1360	0.28	[-0.60; 1.16]	2.0%
Geremia et al. 2018 [72]	15	350	59	15	192	36	3.15	[2.03; 4.26]	1.5%
Kongsgaard et al. 2007 [65]	12	4213	1406	12	3676	1306	0.38	[-0.43; 1.19]	2.2%
Kubo et al. 2001 [51]	8	106	33	8	68	21	1.31	[0.20; 2.42]	1.5%
Kubo et al. 2006 [112]	8	59	24	8	51	22	0.29	[-0.69; 1.28]	1.7%
Kubo et al. 2006 [112]	8	1786	660	8	1790	559	-0.01	[-0.99; 0.97]	1.7%
Kubo et al. 2006 [113]	9	86	36	9	79	21	0.25	[-0.68; 1.17]	1.9%
Kubo et al. 2006 [113]	9	122	40	9	81	26	1.17	[0.15; 2.18]	1.7%
Kubo et al. 2006 [96]	9	59	23	9	46	19	0.62	[-0.33; 1.57]	1.8%
Kubo et al. 2006 [96]	9	1819	710	9	1676	662	0.20	[-0.73; 1.13]	1.9%
Kubo et al. 2007 [85]	10	166	44	10	128	26	1.01	[0.07; 1.96]	1.8%
Kubo et al. 2009 [114]	10	96	37	10	72	20	0.75	[-0.16; 1.67]	1.9%
Kubo et al. 2009 [114]	10	1253	410	10	999	426	0.58	[-0.32; 1.48]	1.9%
Kubo et al. 2009 [114]	10	110	36	10	67	19	1.43	[0.43; 2.44]	1.7%
Kubo et al. 2009 [114]	10	1833	892	10	1071	639	0.94	[0.01; 1.88]	1.8%
Kubo et al. 2010 [115]	10	96	37	10	72	20	0.75	[-0.16; 1.67]	1.9%
Kubo et al. 2010 [115]	10	41	13	10	32	5	0.85	[-0.08; 1.77]	1.9%
Kubo et al. 2010 [88]	8	104	37	8	69	19	1.12	[0.04; 2.20]	1.5%
Kubo et al. 2012 [89]	9	277	111	9	183	45	1.06	[0.05; 2.06]	1.7%
Kubo et al. 2017 [116]	9	1414	355	9	1004	384	1.06	[0.05; 2.06]	1.7%
Kubo et al. 2017 [116]	9	1457	440	9	1289	544	0.32	[-0.61; 1.26]	1.8%
Kubo et al. 2017 [86]	11	32	9	11	24	5	1.17	[0.25; 2.09]	1.9%
Malliaras et al. 2013 [61]	9	2338	638	9	1560	793	1.03	[0.03; 2.03]	1.7%
Malliaras et al. 2013 [61]	10	2536	850	10	1822	898	0.78	[-0.13; 1.70]	1.9%
Malliaras et al. 2013 [61]	10	2508	1066	10	1387	360	1.35	[0.36; 2.34]	1.7%
Massey et al. 2018 [66]	15	687	285	15	560	177	0.52	[-0.21; 1.25]	2.4%
Massey et al. 2018 [66]	15	3239	575	15	2835	444	0.77	[0.02; 1.51]	2.3%
Massey et al. 2018 [66]	14	595	101	14	592	118	0.03	[-0.71; 0.77]	2.4%
Massey et al. 2018 [66]	14	3122	632	14	2605	446	0.92	[0.13; 1.70]	2.2%
Onambélé et al. 2008 [120]	12	40	23	12	26	13	0.72	[-0.11; 1.55]	2.1%
Waugh et al. 2018 [54]	14	541	49	14	390	35	3.43	[2.21; 4.65]	1.3%
Waugh et al. 2018 [54]	14	523	53	14	359	53	2.99	[1.87; 4.12]	1.5%
Random effects model	473			473			0.88	[0.69; 1.07]	81.4%
Heterogeneity: I^2 = 48%, p = 0.38									
**Random effects model**	**577**			**577**			**0.86**	**[0.70; 1.02]**	**100.0%**
Heterogeneity: I^2 = 42%, p < 0.01									
Residual heterogeneity: I^2 = 43%, p < 0.01									

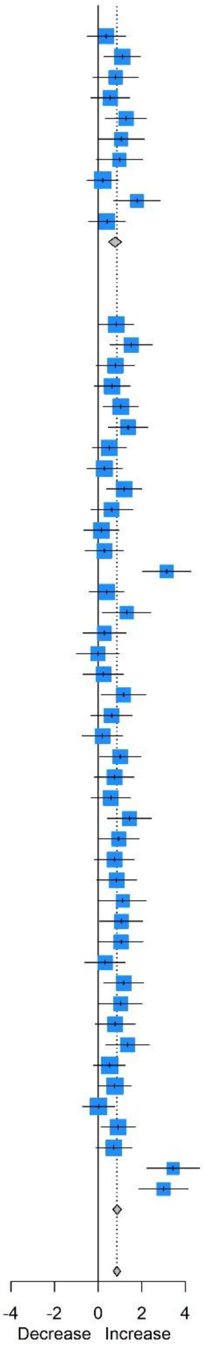

S21. Forest plot for the meta-analysis of modulus subdivided by protocol duration showing standardised mean differences (SMD) and 95% confidence intervals (CI) of high intensity, resistance training studies.

Study	Post n	Post Mean	Post SD	Pre n	Pre Mean	Pre SD	SMD	[95% CI]	Weight
<12 weeks									
McMahon et al. 2013 [67]	10	1100	120	10	830	90	2.44	[1.22; 3.65]	3.0%
McMahon et al. 2013 [67]	11	1150	110	11	780	100	3.39	[2.00; 4.77]	2.5%
McMahon et al. 2018 [68]	8	990	255	8	780	170	0.92	[-0.13; 1.96]	3.5%
McMahon et al. 2018 [68]	8	600	226	8	420	113	0.95	[-0.10; 2.00]	3.5%
Seynnes et al. 2009 [70]	15	1160	1201	15	980	1162	0.15	[-0.57; 0.87]	4.8%
Random effects model	52			52			1.48	[0.38; 2.58]	17.3%
Heterogeneity: I^2 = 82%, p = 0.04									
12 weeks+									
Arampatzis et al. 2007 [19]	11	680	355	11	440	278	0.72	[-0.14; 1.59]	4.2%
Arampatzis et al. 2010 [20]	11	1130	332	11	970	232	0.54	[-0.32; 1.39]	4.2%
Bohm et al. 2014 [21]	12	1050	277	12	890	277	0.56	[-0.26; 1.38]	4.4%
Bohm et al. 2014 [21]	14	1430	636	14	910	262	1.04	[0.24; 1.83]	4.5%
Bohm et al. 2014 [21]	12	1410	381	12	970	277	1.28	[0.38; 2.17]	4.1%
Carroll et al. 2011 [62]	12	1220	450	12	1030	416	0.42	[-0.39; 1.23]	4.4%
Centner et al. 2019 [71]	14	1848	481	14	1540	492	0.61	[-0.15; 1.38]	4.6%
Eriksen et al. 2018 [63]	9	1330	690	9	1230	390	0.17	[-0.76; 1.10]	3.9%
Eriksen et al. 2018 [63]	12	735	263	12	703	253	0.12	[-0.68; 0.92]	4.5%
Eriksen et al. 2019 [64]	10	1560	443	10	1510	474	0.10	[-0.77; 0.98]	4.1%
Geremia et al. 2018 [72]	15	1292	436	15	695	160	1.77	[0.91; 2.63]	4.2%
Kongsgaard et al. 2007 [65]	12	1650	554	12	1470	589	0.30	[-0.50; 1.11]	4.4%
Kubo et al. 2001 [52]	8	433	35	8	288	26	4.45	[2.42; 6.47]	1.4%
Malliaras et al. 2013 [61]	9	942	279	9	620	223	1.22	[0.19; 2.24]	3.6%
Malliaras et al. 2013 [61]	10	1022	339	10	737	390	0.75	[-0.17; 1.66]	4.0%
Malliaras et al. 2013 [61]	10	1011	436	10	570	191	1.25	[0.28; 2.23]	3.8%
Massey et al. 2018 [66]	15	1510	360	15	1320	270	0.58	[-0.15; 1.31]	4.8%
Massey et al. 2018 [66]	14	1490	270	14	1230	180	1.10	[0.30; 1.90]	4.5%
Waugh et al. 2018 [54]	14	1892	803	14	1261	459	0.94	[0.15; 1.72]	4.5%
Waugh et al. 2018 [54]	14	1720	535	14	1242	420	0.96	[0.17; 1.75]	4.5%
Random effects model	238			238			0.80	[0.55; 1.05]	82.7%
Heterogeneity: I^2 = 40%, p < 0.01									
**Random effects model**	**290**			**290**			**0.91**	**[0.63; 1.18]**	**100.0%**
Heterogeneity: I^2 = 57%, p < 0.01									
Residual heterogeneity: I^2 = 57%, p < 0.01									

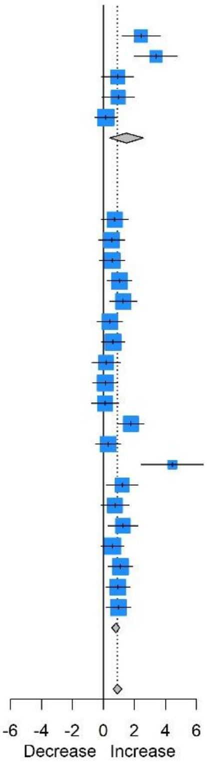

S22. Forest plot for the meta-analysis of cross-sectional area subdivided by protocol duration showing standardised mean differences (SMD) and 95% confidence intervals (CI) of high intensity, resistance training studies.

Study	Post n	Post Mean	Post SD	Pre n	Pre Mean	Pre SD	SMD	[95% CI]	Weight
<12 weeks									
Kubo et al. 2002 [111]	8	59	8	8	61	9	-0.22	[-1.21; 0.76]	1.7%
McMahon et al. 2013 [67]	10	73	14	10	70	13	0.21	[-0.67; 1.09]	2.2%
McMahon et al. 2013 [67]	11	76	16	11	71	13	0.31	[-0.53; 1.16]	2.4%
McMahon et al. 2018 [68]	8	81	13	8	77	13	0.32	[-0.67; 1.30]	1.7%
McMahon et al. 2018 [68]	8	70	22	8	66	11	0.20	[-0.78; 1.18]	1.7%
Mouraux et al. 2000 [118]	10	257	61	10	240	49	0.29	[-0.59; 1.18]	2.2%
Sanz-Lopez et al. 2016 [123]	20	82	23	20	64	11	1.04	[0.38; 1.71]	3.8%
Seynnes et al. 2009 [70]	15	107	4	15	103	4	1.01	[0.24; 1.77]	2.9%
Random effects model	90			90			0.49	[0.17; 0.80]	18.6%
Heterogeneity: $I^2 = 8\%$, $p = 1.00$									
12 weeks+									
Arampatzis et al. 2007 [19]	11	53	14	11	48	13	0.30	[-0.55; 1.14]	2.4%
Arampatzis et al. 2010 [20]	11	56	36	11	56	38	0.02	[-0.82; 0.85]	2.4%
Bohm et al. 2014 [21]	12	82	12	12	78	11	0.36	[-0.45; 1.16]	2.6%
Bohm et al. 2014 [21]	14	83	14	14	80	13	0.23	[-0.52; 0.97]	3.0%
Bohm et al. 2014 [21]	12	79	10	12	75	9	0.34	[-0.47; 1.14]	2.6%
Carroll et al. 2011 [62]	12	115	33	12	117	35	-0.06	[-0.86; 0.74]	2.6%
Centner et al. 2019 [71]	14	74	17	14	70	18	0.18	[-0.56; 0.92]	3.1%
Eriksen et al. 2018 [63]	9	129	24	9	131	27	-0.07	[-1.00; 0.85]	2.0%
Eriksen et al. 2018 [63]	11	123	33	12	121	28	0.06	[-0.76; 0.88]	2.5%
Eriksen et al. 2019 [64]	10	124	22	10	117	21	0.31	[-0.57; 1.20]	2.2%
Geremia et al. 2018 [72]	15	72	11	15	62	8	1.01	[0.24; 1.77]	2.9%
Kongsgaard et al. 2007 [65]	12	121	14	12	117	14	0.28	[-0.53; 1.08]	2.6%
Kubo et al. 2001 [51]	8	213	19	8	210	16	0.16	[-0.82; 1.14]	1.7%
Kubo et al. 2001 [51]	8	215	21	8	212	18	0.15	[-0.84; 1.13]	1.7%
Kubo et al. 2006 [112]	8	73	18	8	73	20	0.01	[-0.97; 0.99]	1.8%
Kubo et al. 2006 [113]	9	207	22	9	204	19	0.14	[-0.79; 1.06]	2.0%
Kubo et al. 2006 [113]	9	205	17	9	202	13	0.19	[-0.74; 1.12]	2.0%
Kubo et al. 2006 [96]	9	78	20	9	78	19	-0.02	[-0.95; 0.90]	2.0%
Kubo et al. 2007 [85]	10	58	8	10	59	8	-0.08	[-0.96; 0.79]	2.2%
Kubo et al. 2009 [114]	10	62	8	10	61	7	0.10	[-0.78; 0.98]	2.2%
Kubo et al. 2009 [114]	10	63	8	10	60	7	0.32	[-0.57; 1.20]	2.2%
Kubo et al. 2010 [115]	10	58	8	10	59	8	-0.08	[-0.96; 0.79]	2.2%
Kubo et al. 2010 [115]	10	68	8	10	66	8	0.21	[-0.67; 1.09]	2.2%
Kubo et al. 2010 [88]	8	81	20	8	80	19	0.04	[-0.94; 1.02]	1.8%
Kubo et al. 2012 [89]	9	74	28	9	72	33	0.06	[-0.86; 0.99]	2.0%
Kubo et al. 2017 [116]	9	73	10	9	72	11	0.08	[-0.84; 1.01]	2.0%
Kubo et al. 2017 [116]	9	74	9	9	74	8	0.03	[-0.89; 0.96]	2.0%
Kubo et al. 2017 [86]	11	66	8	11	65	8	0.14	[-0.70; 0.97]	2.4%
Malliaras et al. 2013 [61]	9	118	9	9	112	6	0.73	[-0.23; 1.70]	1.8%
Malliaras et al. 2013 [61]	10	120	19	10	116	8	0.27	[-0.61; 1.15]	2.2%
Malliaras et al. 2013 [61]	10	120	19	10	114	19	0.33	[-0.55; 1.22]	2.2%
Massey et al. 2018 [66]	14	96	8	14	99	10	-0.30	[-1.04; 0.45]	3.0%
Massey et al. 2018 [66]	15	98	13	15	97	13	0.03	[-0.69; 0.75]	3.3%
Waugh et al. 2018 [54]	14	61	17	14	62	15	-0.08	[-0.82; 0.67]	3.1%
Waugh et al. 2018 [54]	14	60	14	14	58	13	0.14	[-0.60; 0.88]	3.1%
Random effects model	376			377			0.16	[0.01; 0.30]	81.4%
Heterogeneity: $I^2 = 0\%$, $p = 0.37$									
**Random effects model**	**466**			**467**			**0.22**	**[0.09; 0.35]**	**100.0%**
Heterogeneity: $I^2 = 0\%$, $p = 0.99$									
Residual heterogeneity: $I^2 = 0\%$, $p = 1.00$									

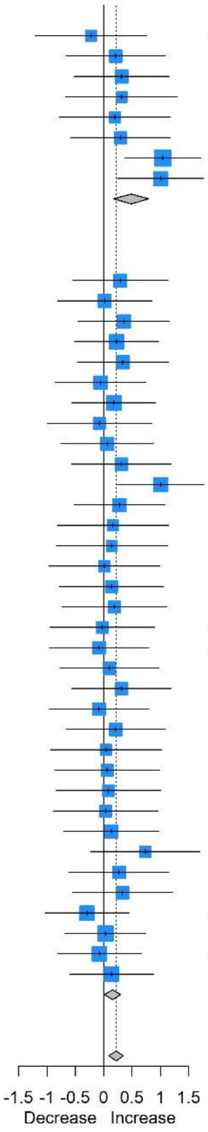

S23. Forest plot for the meta-analysis of stiffness subdivided by contraction mode showing standardised mean differences (SMD) and 95% confidence intervals (CI) of high intensity, resistance training studies.

Study	Post n	Post Mean	Post SD	Pre n	Pre Mean	Pre SD	SMD	[95% CI]	Weight
**Con:Ecc**									
Carroll et al. 2011 [62]	12	3335	1334	12	2928	1320	0.30	[-0.51; 1.10]	2.2%
Centner et al. 2019 [71]	14	565	158	14	402	103	1.19	[0.38; 2.01]	2.1%
Eriksen et al. 2018 [63]	9	3890	2430	9	2560	1530	0.62	[-0.33; 1.58]	1.8%
Eriksen et al. 2018 [63]	12	1900	693	12	1800	624	0.15	[-0.65; 0.95]	2.2%
Eriksen et al. 2019 [64]	10	4420	1075	10	4060	1360	0.28	[-0.60; 1.16]	2.0%
Kongsgaard et al. 2007 [65]	12	4213	1406	12	3676	1306	0.38	[-0.43; 1.19]	2.2%
Kubo et al. 2002 [111]	8	34	10	8	26	9	0.80	[-0.23; 1.83]	1.6%
Kubo et al. 2006 [96]	9	59	23	9	46	19	0.62	[-0.33; 1.57]	1.8%
Kubo et al. 2006 [96]	9	1819	710	9	1676	662	0.20	[-0.73; 1.13]	1.9%
Kubo et al. 2007 [85]	10	166	44	10	128	26	1.01	[0.07; 1.96]	1.8%
Kubo et al. 2009 [114]	10	96	37	10	72	20	0.75	[-0.16; 1.67]	1.9%
Kubo et al. 2009 [114]	10	1253	410	10	999	426	0.58	[-0.32; 1.48]	1.9%
Kubo et al. 2010 [115]	10	96	37	10	72	20	0.75	[-0.16; 1.67]	1.9%
McMahon et al. 2013 [67]	10	1221	594	10	916	441	0.56	[-0.34; 1.46]	1.9%
McMahon et al. 2013 [67]	11	1167	353	11	765	242	1.28	[0.34; 2.21]	1.8%
McMahon et al. 2018 [68]	8	1517	390	8	1132	294	1.05	[-0.01; 2.12]	1.6%
McMahon et al. 2018 [68]	8	887	305	8	619	204	0.98	[-0.08; 2.03]	1.6%
Onambélé et al. 2008 [120]	12	40	23	12	26	13	0.72	[-0.11; 1.55]	2.1%
Seynnes et al. 2009 [70]	15	2288	2115	15	1864	1813	0.21	[-0.51; 0.93]	2.4%
Random effects model	199			199			0.62	[0.41; 0.82]	36.7%
Heterogeneity: $I^2 = 0\%$, $p < 0.01$									
**Concentric**									
Kubo et al. 2017 [116]	9	1414	355	9	1004	384	1.06	[0.05; 2.06]	1.7%
Malliaras et al. 2013 [61]	9	2338	638	9	1560	793	1.03	[0.03; 2.03]	1.7%
Random effects model	18			18			1.04	[0.34; 1.75]	3.4%
Heterogeneity: $I^2 = 0\%$, $p = 0.90$									
**Eccentric**									
Duclay et al. 2009 [105]	10	291	125	10	249	90	0.38	[-0.51; 1.26]	2.0%
Geremia et al. 2018 [72]	15	350	59	15	192	36	3.15	[2.03; 4.26]	1.5%
Kay et al. 2016 [110]	13	13	3	13	10	2	1.10	[0.27; 1.94]	2.1%
Kubo et al. 2017 [116]	9	1457	440	9	1289	544	0.32	[-0.61; 1.26]	1.8%
Malliaras et al. 2013 [61]	10	2536	850	10	1822	898	0.78	[-0.13; 1.70]	1.9%
Malliaras et al. 2013 [61]	10	2508	1066	10	1387	360	1.35	[0.36; 2.34]	1.7%
Random effects model	67			67			1.14	[0.40; 1.89]	11.0%
Heterogeneity: $I^2 = 73\%$, $p < 0.01$									
**Isometric**									
Albracht et al. 2013 [5]	13	315	53	13	272	48	0.82	[0.02; 1.63]	2.2%
Arampatzis et al. 2007 [19]	11	228	40	11	168	37	1.52	[0.55; 2.49]	1.8%
Arampatzis et al. 2010 [20]	11	302	57	11	258	51	0.79	[-0.09; 1.66]	2.0%
Bohm et al. 2014 [21]	12	457	132	12	370	132	0.64	[-0.18; 1.47]	2.1%
Bohm et al. 2014 [21]	14	539	254	14	336	89	1.04	[0.24; 1.84]	2.2%
Bohm et al. 2014 [21]	12	579	172	12	377	106	1.37	[0.46; 2.27]	1.9%
Bohm et al. 2021 [17]	13	111	59	13	85	36	0.52	[-0.27; 1.30]	2.2%
Kubo et al. 2001 [51]	8	106	33	8	68	21	1.31	[0.20; 2.42]	1.5%
Kubo et al. 2006 [112]	8	59	24	8	51	22	0.29	[-0.69; 1.28]	1.7%
Kubo et al. 2006 [112]	8	1786	660	8	1790	559	-0.01	[-0.99; 0.97]	1.7%
Kubo et al. 2006 [113]	9	86	36	9	79	21	0.25	[-0.68; 1.17]	1.9%
Kubo et al. 2006 [113]	9	122	40	9	81	26	1.17	[0.15; 2.18]	1.7%
Kubo et al. 2009 [114]	10	110	36	10	67	19	1.43	[0.43; 2.44]	1.7%
Kubo et al. 2009 [114]	10	1833	892	10	1071	639	0.94	[0.01; 1.88]	1.8%
Kubo et al. 2010 [115]	10	41	13	10	32	5	0.85	[-0.08; 1.77]	1.9%
Kubo et al. 2010 [88]	8	104	37	8	69	19	1.12	[0.04; 2.20]	1.5%
Kubo et al. 2012 [89]	9	277	111	9	183	45	1.06	[0.05; 2.06]	1.7%
Kubo et al. 2017 [86]	11	32	9	11	24	5	1.17	[0.25; 2.09]	1.9%
Massey et al. 2018 [66]	15	687	285	15	560	177	0.52	[-0.21; 1.25]	2.4%
Massey et al. 2018 [66]	15	3239	575	15	2835	444	0.77	[0.02; 1.51]	2.3%
Massey et al. 2018 [66]	14	595	101	14	592	118	0.03	[-0.71; 0.77]	2.4%
Massey et al. 2018 [66]	14	3122	632	14	2605	446	0.92	[0.13; 1.70]	2.2%
Tillin et al. 2012 [125]	10	697	103	10	520	86	1.79	[0.71; 2.86]	1.5%
Waugh et al. 2018 [54]	14	541	49	14	390	35	3.43	[2.21; 4.65]	1.3%
Waugh et al. 2018 [54]	14	523	53	14	359	53	2.99	[1.87; 4.12]	1.5%
Werkhausen et al. 2018 [56]	11	459	147	11	397	146	0.41	[-0.44; 1.25]	2.1%
Random effects model	293			293			0.98	[0.72; 1.24]	49.0%
Heterogeneity: $I^2 = 53\%$, $p = 0.97$									
**Random effects model**	**577**			**577**			**0.86**	**[0.70; 1.02]**	**100.0%**
Heterogeneity: $I^2 = 42\%$, $p < 0.01$									
Residual heterogeneity: $I^2 = 41\%$, $p < 0.01$									

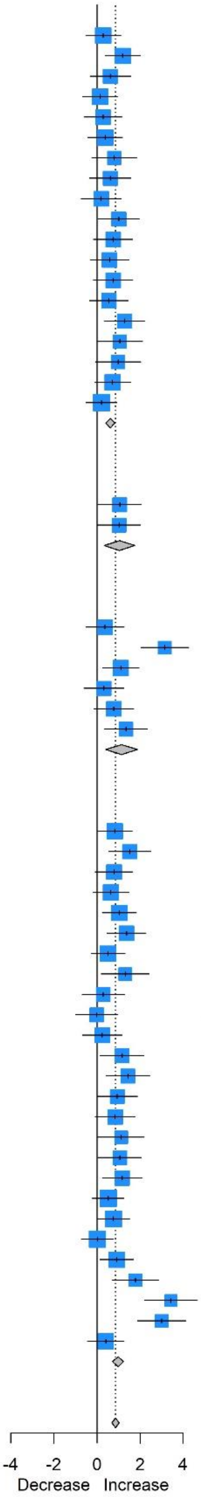

S24. Forest plot for the meta-analysis of modulus subdivided by contraction mode showing standardised mean differences (SMD) and 95% confidence intervals (CI) of high intensity, resistance training studies.

Study	Post n	Post Mean	Post SD	Pre n	Pre Mean	Pre SD	SMD	[95% CI]	Weight
**Con:Ecc**									
Carroll et al. 2011 [62]	12	1220	450	12	1030	416	0.42	[-0.39; 1.23]	4.4%
Centner et al. 2019 [71]	14	1848	481	14	1540	492	0.61	[-0.15; 1.38]	4.6%
Eriksen et al. 2018 [63]	9	1330	690	9	1230	390	0.17	[-0.76; 1.10]	3.9%
Eriksen et al. 2018 [63]	12	735	263	12	703	253	0.12	[-0.68; 0.92]	4.5%
Eriksen et al. 2019 [64]	10	1560	443	10	1510	474	0.10	[-0.77; 0.98]	4.1%
Kongsgaard et al. 2007 [65]	12	1650	554	12	1470	589	0.30	[-0.50; 1.11]	4.4%
McMahon et al. 2013 [67]	10	1100	120	10	830	90	2.44	[1.22; 3.65]	3.0%
McMahon et al. 2013 [67]	11	1150	110	11	780	100	3.39	[2.00; 4.77]	2.5%
McMahon et al. 2018 [68]	8	990	255	8	780	170	0.92	[-0.13; 1.96]	3.5%
McMahon et al. 2018 [68]	8	600	226	8	420	113	0.95	[-0.10; 2.00]	3.5%
Seynnes et al. 2009 [70]	15	1160	1201	15	980	1162	0.15	[-0.57; 0.87]	4.8%
Random effects model	121			121			0.74	[0.26; 1.23]	43.4%
Heterogeneity: $I^2 = 67\%$, $p = 0.09$									
**Concentric**									
Malliaras et al. 2013 [61]	9	942	279	9	620	223	1.22	[0.19; 2.24]	3.6%
Random effects model	9			9			1.22	[0.19; 2.24]	3.6%
Heterogeneity: not applicable									
**Eccentric**									
Geremia et al. 2018 [72]	15	1292	436	15	695	160	1.77	[0.91; 2.63]	4.2%
Malliaras et al. 2013 [61]	10	1022	339	10	737	390	0.75	[-0.17; 1.66]	4.0%
Malliaras et al. 2013 [61]	10	1011	436	10	570	191	1.25	[0.28; 2.23]	3.8%
Random effects model	35			35			1.27	[0.67; 1.87]	12.0%
Heterogeneity: $I^2 = 22\%$, $p = 0.28$									
**Isometric**									
Arampatzis et al. 2007 [19]	11	680	355	11	440	278	0.72	[-0.14; 1.59]	4.2%
Arampatzis et al. 2010 [20]	11	1130	332	11	970	232	0.54	[-0.32; 1.39]	4.2%
Bohm et al. 2014 [21]	12	1050	277	12	890	277	0.56	[-0.26; 1.38]	4.4%
Bohm et al. 2014 [21]	14	1430	636	14	910	262	1.04	[0.24; 1.83]	4.5%
Bohm et al. 2014 [21]	12	1410	381	12	970	277	1.28	[0.38; 2.17]	4.1%
Kubo et al. 2001 [52]	8	433	35	8	288	26	4.45	[2.42; 6.47]	1.4%
Massey et al. 2018 [66]	15	1510	360	15	1320	270	0.58	[-0.15; 1.31]	4.8%
Massey et al. 2018 [66]	14	1490	270	14	1230	180	1.10	[0.30; 1.90]	4.5%
Waugh et al. 2018 [54]	14	1892	803	14	1261	459	0.94	[0.15; 1.72]	4.5%
Waugh et al. 2018 [54]	14	1720	535	14	1242	420	0.96	[0.17; 1.75]	4.5%
Random effects model	125			125			0.95	[0.60; 1.30]	41.1%
Heterogeneity: $I^2 = 40\%$, p = NA									
**Random effects model**	**290**			**290**			**0.91**	**[0.63; 1.18]**	**100.0%**
Heterogeneity: $I^2 = 57\%$, $p < 0.01$									
Residual heterogeneity: $I^2 = 57\%$, $p < 0.01$									

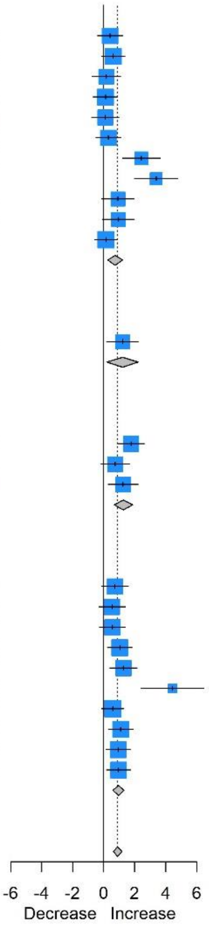

S25. Forest plot for the meta-analysis of cross-sectional area subdivided by contraction mode showing standardised mean differences (SMD) and 95% confidence intervals (CI) of high intensity, resistance training studies.

Study	Post n	Post Mean	Post SD	Pre n	Pre Mean	Pre SD	SMD	[95% CI]	Weight
**Con:Ecc**									
Carroll et al. 2011 [62]	12	115	33	12	117	35	-0.06	[-0.86; 0.74]	2.6%
Centner et al. 2019 [71]	14	74	17	14	70	18	0.18	[-0.56; 0.92]	3.1%
Eriksen et al. 2018 [63]	9	129	24	9	131	27	-0.07	[-1.00; 0.85]	2.0%
Eriksen et al. 2018 [63]	11	123	33	12	121	28	0.06	[-0.76; 0.88]	2.5%
Eriksen et al. 2019 [64]	10	124	22	10	117	21	0.31	[-0.57; 1.20]	2.2%
Kongsgaard et al. 2007 [65]	12	121	14	12	117	14	0.28	[-0.53; 1.08]	2.6%
Kubo et al. 2002 [111]	8	59	8	8	61	9	-0.22	[-1.21; 0.76]	1.7%
Kubo et al. 2006 [96]	9	78	20	9	78	19	-0.02	[-0.95; 0.90]	2.0%
Kubo et al. 2007 [85]	10	58	8	10	59	8	-0.08	[-0.96; 0.79]	2.2%
Kubo et al. 2009 [114]	10	62	8	10	61	7	0.10	[-0.78; 0.98]	2.2%
Kubo et al. 2010 [115]	10	58	8	10	59	8	-0.08	[-0.96; 0.79]	2.2%
Kubo et al. 2010 [115]	10	68	8	10	66	8	0.21	[-0.67; 1.09]	2.2%
McMahon et al. 2013 [67]	10	73	14	10	70	13	0.21	[-0.67; 1.09]	2.2%
McMahon et al. 2013 [67]	11	76	16	11	71	13	0.31	[-0.53; 1.16]	2.4%
McMahon et al. 2018 [68]	8	81	13	8	77	13	0.32	[-0.67; 1.30]	1.7%
McMahon et al. 2018 [68]	8	70	22	8	66	11	0.20	[-0.78; 1.18]	1.7%
Seynnes et al. 2009 [70]	15	107	4	15	103	4	1.01	[0.24; 1.77]	2.9%
Random effects model	177			178			0.17	[-0.04; 0.38]	38.2%
Heterogeneity: $I^2 = 0\%$, $p = 1.00$									
**Concentric**									
Kubo et al. 2017 [116]	9	73	10	9	72	11	0.08	[-0.84; 1.01]	2.0%
Malliaras et al. 2013 [61]	9	118	9	9	112	6	0.73	[-0.23; 1.70]	1.8%
Random effects model	18			18			0.40	[-0.27; 1.06]	3.8%
Heterogeneity: $I^2 = 0\%$, $p = 0.97$									
**Eccentric**									
Geremia et al. 2018 [72]	15	72	11	15	62	8	1.01	[0.24; 1.77]	2.9%
Kubo et al. 2017 [116]	9	74	9	9	74	8	0.03	[-0.89; 0.96]	2.0%
Malliaras et al. 2013 [61]	10	120	19	10	116	8	0.27	[-0.61; 1.15]	2.2%
Malliaras et al. 2013 [61]	10	120	19	10	114	19	0.33	[-0.55; 1.22]	2.2%
Mouraux et al. 2000 [118]	10	257	61	10	240	49	0.29	[-0.59; 1.18]	2.2%
Sanz-Lopez et al. 2016 [123]	20	82	23	20	64	11	1.04	[0.38; 1.71]	3.8%
Random effects model	74			74			0.58	[0.22; 0.93]	15.1%
Heterogeneity: $I^2 = 10\%$, $p = 0.35$									
**Isometric**									
Arampatzis et al. 2007 [19]	11	53	14	11	48	13	0.30	[-0.55; 1.14]	2.4%
Arampatzis et al. 2010 [20]	11	56	36	11	56	38	0.02	[-0.82; 0.85]	2.4%
Bohm et al. 2014 [21]	12	82	12	12	78	11	0.36	[-0.45; 1.16]	2.6%
Bohm et al. 2014 [21]	14	83	14	14	80	13	0.23	[-0.52; 0.97]	3.0%
Bohm et al. 2014 [21]	12	79	10	12	75	9	0.34	[-0.47; 1.14]	2.6%
Kubo et al. 2001 [51]	8	213	19	8	210	16	0.16	[-0.82; 1.14]	1.7%
Kubo et al. 2001 [51]	8	215	21	8	212	18	0.15	[-0.84; 1.13]	1.7%
Kubo et al. 2006 [112]	8	73	18	8	73	20	0.01	[-0.97; 0.99]	1.8%
Kubo et al. 2006 [113]	9	207	22	9	204	19	0.14	[-0.79; 1.06]	2.0%
Kubo et al. 2006 [113]	9	205	17	9	202	13	0.19	[-0.74; 1.12]	2.0%
Kubo et al. 2009 [114]	10	63	8	10	60	7	0.32	[-0.57; 1.20]	2.2%
Kubo et al. 2010 [88]	8	81	20	8	80	19	0.04	[-0.94; 1.02]	1.8%
Kubo et al. 2012 [89]	9	74	28	9	72	33	0.06	[-0.86; 0.99]	2.0%
Kubo et al. 2017 [86]	11	66	8	11	65	8	0.14	[-0.70; 0.97]	2.4%
Massey et al. 2018 [66]	14	96	8	14	99	10	-0.30	[-1.04; 0.45]	3.0%
Massey et al. 2018 [66]	15	98	13	15	97	13	0.03	[-0.69; 0.75]	3.3%
Waugh et al. 2018 [54]	14	61	17	14	62	15	-0.08	[-0.82; 0.67]	3.1%
Waugh et al. 2018 [54]	14	60	14	14	58	13	0.14	[-0.60; 0.88]	3.1%
Random effects model	197			197			0.12	[-0.08; 0.32]	42.9%
Heterogeneity: $I^2 = 0\%$, $p = 0.34$									
**Random effects model**	**466**			**467**			**0.22**	**[0.09; 0.35]**	**100.0%**
Heterogeneity: $I^2 = 0\%$, $p = 0.99$									
Residual heterogeneity: $I^2 = 0\%$, $p = 1.00$									

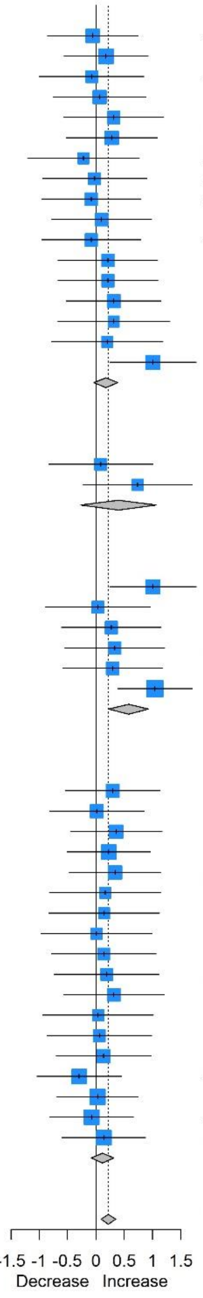

S26. Forest plot for the meta-analysis of stiffness subdivided by contraction mode showing standardised mean differences (SMD) and 95% confidence intervals (CI) of matched high intensity, resistance training studies.

Study	Post n	Post Mean	Post SD	Pre n	Pre Mean	Pre SD	SMD	[95% CI]	Weight
**Con:Ecc**									
Kubo et al. 2009 [114]	10	96	37	10	72	20	0.75	[-0.16; 1.67]	12.1%
Kubo et al. 2009 [114]	10	1253	410	10	999	426	0.58	[-0.32; 1.48]	12.5%
Random effects model	20			20			0.67	[0.02; 1.31]	24.5%
Heterogeneity: $I^2 = 0\%$, $p = 0.80$									
**Concentric**									
Kubo et al. 2017 [116]	9	1414	355	9	1004	384	1.06	[0.05; 2.06]	10.0%
Malliaras et al. 2013 [61]	9	2338	638	9	1560	793	1.03	[0.03; 2.03]	10.1%
Random effects model	18			18			1.04	[0.34; 1.75]	20.1%
Heterogeneity: $I^2 = 0\%$, $p = 0.48$									
**Eccentric**									
Kubo et al. 2017 [116]	9	1457	440	9	1289	544	0.32	[-0.61; 1.26]	11.6%
Malliaras et al. 2013 [61]	10	2536	850	10	1822	898	0.78	[-0.13; 1.70]	12.0%
Malliaras et al. 2013 [61]	10	2508	1066	10	1387	360	1.35	[0.36; 2.34]	10.2%
Random effects model	29			29			0.80	[0.23; 1.37]	33.8%
Heterogeneity: $I^2 = 8\%$, $p = 0.97$									
**Isometric**									
Kubo et al. 2009 [114]	10	110	36	10	67	19	1.43	[0.43; 2.44]	9.9%
Kubo et al. 2009 [114]	10	1833	892	10	1071	639	0.94	[0.01; 1.88]	11.5%
Random effects model	20			20			1.17	[0.48; 1.85]	21.5%
Heterogeneity: $I^2 = 0\%$, $p = 0.34$									
**Random effects model**	**87**			**87**			**0.89**	**[0.58; 1.21]**	**100.0%**
Heterogeneity: $I^2 = 0\%$, $p = 0.84$									
Residual heterogeneity: $I^2 = 0\%$, $p = 0.74$									

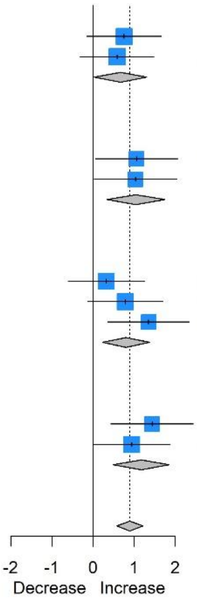

S27. Forest plot for the meta-analysis of modulus subdivided by contraction mode showing standardised mean differences (SMD) and 95% confidence intervals (CI) of matched high intensity, resistance training studies.

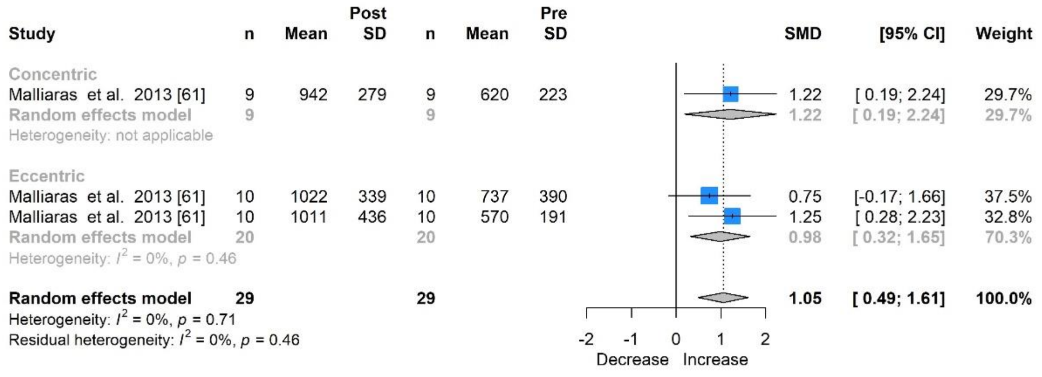

S28. Forest plot for the meta-analysis of cross-sectional area subdivided by contraction mode showing standardised mean differences (SMD) and 95% confidence intervals (CI) of matched high intensity, resistance training studies.

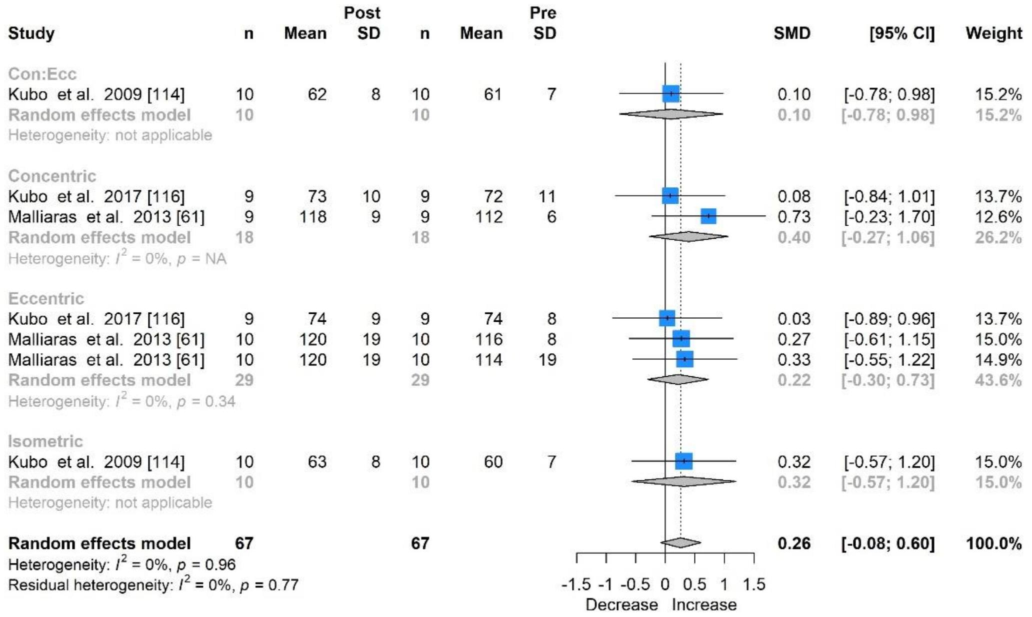

S29. Forest plot for the meta-analysis of stiffness subdivided by age group showing standardised mean differences (SMD) and 95% confidence intervals (CI) of high intensity resistance training studies.

Study	n	Mean	Post SD	n	Mean	Pre SD	SMD	[95% CI]	Weight
**Adult**									
Albracht et al. 2013 [5]	13	315	53	13	272	48	0.82	[0.02; 1.63]	2.2%
Arampatzis et al. 2007 [19]	11	228	40	11	168	37	1.52	[0.55; 2.49]	1.8%
Arampatzis et al. 2010 [20]	11	302	57	11	258	51	0.79	[-0.09; 1.66]	2.0%
Bohm et al. 2014 [21]	12	457	132	12	370	132	0.64	[-0.18; 1.47]	2.1%
Bohm et al. 2014 [21]	14	539	254	14	336	89	1.04	[0.24; 1.84]	2.2%
Bohm et al. 2014 [21]	12	579	172	12	377	106	1.37	[0.46; 2.27]	1.9%
Bohm et al. 2021 [17]	13	111	59	13	85	36	0.52	[-0.27; 1.30]	2.2%
Centner et al. 2019 [71]	14	565	158	14	402	103	1.19	[0.38; 2.01]	2.1%
Duclay et al. 2009 [105]	10	291	125	10	249	90	0.38	[-0.51; 1.26]	2.0%
Geremia et al. 2018 [72]	15	350	59	15	192	36	3.15	[2.03; 4.26]	1.5%
Kay et al. 2016 [110]	13	13	3	13	10	2	1.10	[0.27; 1.94]	2.1%
Kongsgaard et al. 2007 [65]	12	4213	1406	12	3676	1306	0.38	[-0.43; 1.19]	2.2%
Kubo et al. 2001 [51]	8	106	33	8	68	21	1.31	[0.20; 2.42]	1.5%
Kubo et al. 2002 [111]	8	34	10	8	26	9	0.80	[-0.23; 1.83]	1.6%
Kubo et al. 2006 [112]	8	59	24	8	51	22	0.29	[-0.69; 1.28]	1.7%
Kubo et al. 2006 [112]	8	1786	660	8	1790	559	-0.01	[-0.99; 0.97]	1.7%
Kubo et al. 2006 [113]	9	86	36	9	79	21	0.25	[-0.68; 1.17]	1.9%
Kubo et al. 2006 [113]	9	122	40	9	81	26	1.17	[0.15; 2.18]	1.7%
Kubo et al. 2006 [96]	9	59	23	9	46	19	0.62	[-0.33; 1.57]	1.8%
Kubo et al. 2006 [96]	9	1819	710	9	1676	662	0.20	[-0.73; 1.13]	1.9%
Kubo et al. 2007 [85]	10	166	44	10	128	26	1.01	[0.07; 1.96]	1.8%
Kubo et al. 2009 [114]	10	96	37	10	72	20	0.75	[-0.16; 1.67]	1.9%
Kubo et al. 2009 [114]	10	1253	410	10	999	426	0.58	[-0.32; 1.48]	1.9%
Kubo et al. 2009 [114]	10	110	36	10	67	19	1.43	[0.43; 2.44]	1.7%
Kubo et al. 2009 [114]	10	1833	892	10	1071	639	0.94	[0.01; 1.88]	1.8%
Kubo et al. 2010 [115]	10	96	37	10	72	20	0.75	[-0.16; 1.67]	1.9%
Kubo et al. 2010 [115]	10	41	13	10	32	5	0.85	[-0.08; 1.77]	1.9%
Kubo et al. 2010 [88]	8	104	37	8	69	19	1.12	[0.04; 2.20]	1.5%
Kubo et al. 2012 [89]	9	277	111	9	183	45	1.06	[0.05; 2.06]	1.7%
Kubo et al. 2017 [116]	9	1414	355	9	1004	384	1.06	[0.05; 2.06]	1.7%
Kubo et al. 2017 [116]	9	1457	440	9	1289	544	0.32	[-0.61; 1.26]	1.8%
Kubo et al. 2017 [86]	11	32	9	11	24	5	1.17	[0.25; 2.09]	1.9%
Malliaras et al. 2013 [61]	9	2338	638	9	1560	793	1.03	[0.03; 2.03]	1.7%
Malliaras et al. 2013 [61]	10	2536	850	10	1822	898	0.78	[-0.13; 1.70]	1.9%
Malliaras et al. 2013 [61]	10	2508	1066	10	1387	360	1.35	[0.36; 2.34]	1.7%
Massey et al. 2018 [66]	15	687	285	15	560	177	0.52	[-0.21; 1.25]	2.4%
Massey et al. 2018 [66]	15	3239	575	15	2835	444	0.77	[0.02; 1.51]	2.3%
Massey et al. 2018 [66]	14	595	101	14	592	118	0.03	[-0.71; 0.77]	2.4%
Massey et al. 2018 [66]	14	3122	632	14	2605	446	0.92	[0.13; 1.70]	2.2%
McMahon et al. 2013 [67]	10	1221	594	10	916	441	0.56	[-0.34; 1.46]	1.9%
McMahon et al. 2013 [67]	11	1167	353	11	765	242	1.28	[0.34; 2.21]	1.8%
McMahon et al. 2018 [68]	8	1517	390	8	1132	294	1.05	[-0.01; 2.12]	1.6%
McMahon et al. 2018 [68]	8	887	305	8	619	204	0.98	[-0.08; 2.03]	1.6%
Seynnes et al. 2009 [70]	15	2288	2115	15	1864	1813	0.21	[-0.51; 0.93]	2.4%
Tillin et al. 2012 [125]	10	697	103	10	520	86	1.79	[0.71; 2.86]	1.5%
Waugh et al. 2018 [54]	14	541	49	14	390	35	3.43	[2.21; 4.65]	1.3%
Waugh et al. 2018 [54]	14	523	53	14	359	53	2.99	[1.87; 4.12]	1.5%
Werkhausen et al. 2018 [56]	11	459	147	11	397	146	0.41	[-0.44; 1.25]	2.1%
Random effects model	522			522			0.91	[0.74; 1.09]	89.8%
Heterogeneity: $I^2 = 44\%$, $p < 0.01$									
**Elderly**									
Carroll et al. 2011 [62]	12	3335	1334	12	2928	1320	0.30	[-0.51; 1.10]	2.2%
Eriksen et al. 2018 [63]	9	3890	2430	9	2560	1530	0.62	[-0.33; 1.58]	1.8%
Eriksen et al. 2018 [63]	12	1900	693	12	1800	624	0.15	[-0.65; 0.95]	2.2%
Eriksen et al. 2019 [64]	10	4420	1075	10	4060	1360	0.28	[-0.60; 1.16]	2.0%
Onambélé et al. 2008 [120]	12	40	23	12	26	13	0.72	[-0.11; 1.55]	2.1%
Random effects model	55			55			0.40	[0.02; 0.78]	10.2%
Heterogeneity: $I^2 = 0\%$, $p = 0.86$									
**Random effects model**	**577**			**577**			**0.86**	**[0.70; 1.02]**	**100.0%**
Heterogeneity: $I^2 = 42\%$, $p < 0.01$									
Residual heterogeneity: $I^2 = 40\%$, $p < 0.01$									

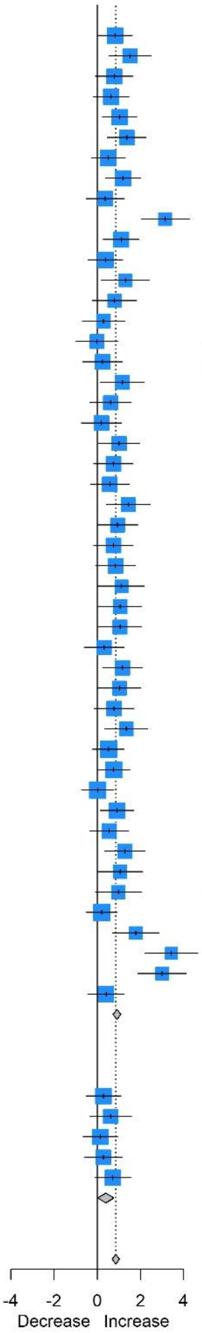

<u>S30.</u> Forest plot for the meta-analysis of modulus subdivided by age group showing standardised mean differences (SMD) and 95% confidence intervals (CI) of high intensity resistance training studies.

Study	Post n	Post Mean	Post SD	Pre n	Pre Mean	Pre SD	SMD	[95% CI]	Weight
**Adult**									
Arampatzis et al. 2007 [19]	11	680	355	11	440	278	0.72	[-0.14; 1.59]	4.2%
Arampatzis et al. 2010 [20]	11	1130	332	11	970	232	0.54	[-0.32; 1.39]	4.2%
Bohm et al. 2014 [21]	12	1050	277	12	890	277	0.56	[-0.26; 1.38]	4.4%
Bohm et al. 2014 [21]	14	1430	636	14	910	262	1.04	[0.24; 1.83]	4.5%
Bohm et al. 2014 [21]	12	1410	381	12	970	277	1.28	[0.38; 2.17]	4.1%
Centner et al. 2019 [71]	14	1848	481	14	1540	492	0.61	[-0.15; 1.38]	4.6%
Geremia et al. 2018 [72]	15	1292	436	15	695	160	1.77	[0.91; 2.63]	4.2%
Kongsgaard et al. 2007 [65]	12	1650	554	12	1470	589	0.30	[-0.50; 1.11]	4.4%
Kubo et al. 2001 [52]	8	433	35	8	288	26	4.45	[2.42; 6.47]	1.4%
Malliaras et al. 2013 [61]	9	942	279	9	620	223	1.22	[0.19; 2.24]	3.6%
Malliaras et al. 2013 [61]	10	1022	339	10	737	390	0.75	[-0.17; 1.66]	4.0%
Malliaras et al. 2013 [61]	10	1011	436	10	570	191	1.25	[0.28; 2.23]	3.8%
Massey et al. 2018 [66]	15	1510	360	15	1320	270	0.58	[-0.15; 1.31]	4.8%
Massey et al. 2018 [66]	14	1490	270	14	1230	180	1.10	[0.30; 1.90]	4.5%
McMahon et al. 2013 [67]	10	1100	120	10	830	90	2.44	[1.22; 3.65]	3.0%
McMahon et al. 2013 [67]	11	1150	110	11	780	100	3.39	[2.00; 4.77]	2.5%
McMahon et al. 2018 [68]	8	990	255	8	780	170	0.92	[-0.13; 1.96]	3.5%
McMahon et al. 2018 [68]	8	600	226	8	420	113	0.95	[-0.10; 2.00]	3.5%
Seynnes et al. 2009 [70]	15	1160	1201	15	980	1162	0.15	[-0.57; 0.87]	4.8%
Waugh et al. 2018 [54]	14	1892	803	14	1261	459	0.94	[0.15; 1.72]	4.5%
Waugh et al. 2018 [54]	14	1720	535	14	1242	420	0.96	[0.17; 1.75]	4.5%
Random effects model	247			247			1.05	[0.75; 1.34]	83.0%
Heterogeneity: $I^2 = 56\%$, $p < 0.01$									
**Elderly**									
Carroll et al. 2011 [62]	12	1220	450	12	1030	416	0.42	[-0.39; 1.23]	4.4%
Eriksen et al. 2018 [63]	9	1330	690	9	1230	390	0.17	[-0.76; 1.10]	3.9%
Eriksen et al. 2018 [63]	12	735	263	12	703	253	0.12	[-0.68; 0.92]	4.5%
Eriksen et al. 2019 [64]	10	1560	443	10	1510	474	0.10	[-0.77; 0.98]	4.1%
Random effects model	43			43			0.21	[-0.21; 0.63]	17.0%
Heterogeneity: $I^2 = 0\%$, $p = 0.94$									
**Random effects model**	**290**			**290**			**0.91**	**[0.63; 1.18]**	**100.0%**
Heterogeneity: $I^2 = 57\%$, $p < 0.01$									
Residual heterogeneity: $I^2 = 49\%$, $p < 0.01$									

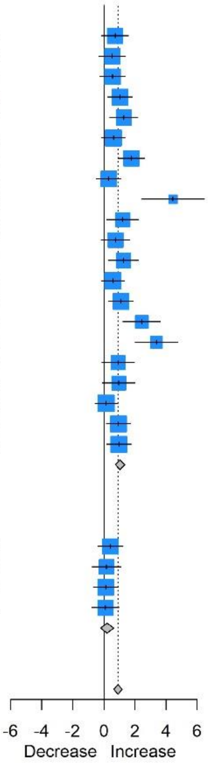

S31. Forest plot for the meta-analysis of cross-sectional area subdivided by age group showing standardised mean differences (SMD) and 95% confidence intervals (CI) of high intensity resistance training studies.

Study	Post n	Post Mean	Post SD	Pre n	Pre Mean	Pre SD	SMD	[95% CI]	Weight
**Adult**									
Arampatzis et al. 2007 [19]	11	53	14	11	48	13	0.30	[-0.55; 1.14]	2.4%
Arampatzis et al. 2010 [20]	11	56	36	11	56	38	0.02	[-0.82; 0.85]	2.4%
Bohm et al. 2014 [21]	12	82	12	12	78	11	0.36	[-0.45; 1.16]	2.6%
Bohm et al. 2014 [21]	14	83	14	14	80	13	0.23	[-0.52; 0.97]	3.0%
Bohm et al. 2014 [21]	12	79	10	12	75	9	0.34	[-0.47; 1.14]	2.6%
Centner et al. 2019 [71]	14	74	17	14	70	18	0.18	[-0.56; 0.92]	3.1%
Geremia et al. 2018 [72]	15	72	11	15	62	8	1.01	[0.24; 1.77]	2.9%
Kongsgaard et al. 2007 [65]	12	121	14	12	117	14	0.28	[-0.53; 1.08]	2.6%
Kubo et al. 2001 [51]	8	213	19	8	210	16	0.16	[-0.82; 1.14]	1.7%
Kubo et al. 2001 [51]	8	215	21	8	212	18	0.15	[-0.84; 1.13]	1.7%
Kubo et al. 2002 [111]	8	59	8	8	61	9	-0.22	[-1.21; 0.76]	1.7%
Kubo et al. 2006 [112]	8	73	18	8	73	20	0.01	[-0.97; 0.99]	1.8%
Kubo et al. 2006 [113]	9	207	22	9	204	19	0.14	[-0.79; 1.06]	2.0%
Kubo et al. 2006 [113]	9	205	17	9	202	13	0.19	[-0.74; 1.12]	2.0%
Kubo et al. 2006 [96]	9	78	20	9	78	19	-0.02	[-0.95; 0.90]	2.0%
Kubo et al. 2007 [85]	10	58	8	10	59	8	-0.08	[-0.96; 0.79]	2.2%
Kubo et al. 2009 [114]	10	62	8	10	61	7	0.10	[-0.78; 0.98]	2.2%
Kubo et al. 2009 [114]	10	63	8	10	60	7	0.32	[-0.57; 1.20]	2.2%
Kubo et al. 2010 [115]	10	58	8	10	59	8	-0.08	[-0.96; 0.79]	2.2%
Kubo et al. 2010 [115]	10	68	8	10	66	8	0.21	[-0.67; 1.09]	2.2%
Kubo et al. 2010 [88]	8	81	20	8	80	19	0.04	[-0.94; 1.02]	1.8%
Kubo et al. 2012 [89]	9	74	28	9	72	33	0.06	[-0.86; 0.99]	2.0%
Kubo et al. 2017 [116]	9	73	10	9	72	11	0.08	[-0.84; 1.01]	2.0%
Kubo et al. 2017 [116]	9	74	9	9	74	8	0.03	[-0.89; 0.96]	2.0%
Kubo et al. 2017 [86]	11	66	8	11	65	8	0.14	[-0.70; 0.97]	2.4%
Malliaras et al. 2013 [61]	9	118	9	9	112	6	0.73	[-0.23; 1.70]	1.8%
Malliaras et al. 2013 [61]	10	120	19	10	116	8	0.27	[-0.61; 1.15]	2.2%
Malliaras et al. 2013 [61]	10	120	19	10	114	19	0.33	[-0.55; 1.22]	2.2%
Massey et al. 2018 [66]	14	96	8	14	99	10	-0.30	[-1.04; 0.45]	3.0%
Massey et al. 2018 [66]	15	98	13	15	97	13	0.03	[-0.69; 0.75]	3.3%
McMahon et al. 2013 [67]	10	73	14	10	70	13	0.21	[-0.67; 1.09]	2.2%
McMahon et al. 2013 [67]	11	76	16	11	71	13	0.31	[-0.53; 1.16]	2.4%
McMahon et al. 2018 [68]	8	81	13	8	77	13	0.32	[-0.67; 1.30]	1.7%
McMahon et al. 2018 [68]	8	70	22	8	66	11	0.20	[-0.78; 1.18]	1.7%
Mouraux et al. 2000 [118]	10	257	61	10	240	49	0.29	[-0.59; 1.18]	2.2%
Sanz-Lopez et al. 2016 [123]	20	82	23	20	64	11	1.04	[0.38; 1.71]	3.8%
Seynnes et al. 2009 [70]	15	107	4	15	103	4	1.01	[0.24; 1.77]	2.9%
Waugh et al. 2018 [54]	14	61	17	14	62	15	-0.08	[-0.82; 0.67]	3.1%
Waugh et al. 2018 [54]	14	60	14	14	58	13	0.14	[-0.60; 0.88]	3.1%
Random effects model	424			424			0.24	[0.10; 0.37]	90.7%
Heterogeneity: $I^2 = 0\%$, $p = 0.99$									
**Elderly**									
Carroll et al. 2011 [62]	12	115	33	12	117	35	-0.06	[-0.86; 0.74]	2.6%
Eriksen et al. 2018 [63]	9	129	24	9	131	27	-0.07	[-1.00; 0.85]	2.0%
Eriksen et al. 2018 [63]	11	123	33	12	121	28	0.06	[-0.76; 0.88]	2.5%
Eriksen et al. 2019 [64]	10	124	22	10	117	21	0.31	[-0.57; 1.20]	2.2%
Random effects model	42			43			0.06	[-0.37; 0.48]	9.3%
Heterogeneity: $I^2 = 0\%$, $p = 0.92$									
**Random effects model**	**466**			**467**			**0.22**	**[0.09; 0.35]**	**100.0%**
Heterogeneity: $I^2 = 0\%$, $p = 0.99$									
Residual heterogeneity: $I^2 = 0\%$, $p = 0.99$									

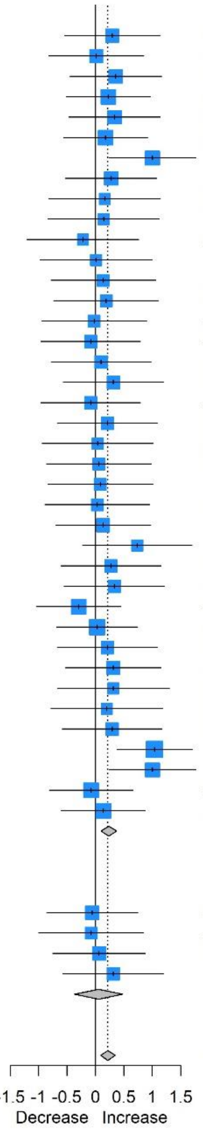

Supplement: Supplementary file 1 — Supplementary file1 (PDF 7231 kb) [file 40279_2022_1695_MOESM1_ESM.pdf]
